# Dimerisation, rhodium complex formation and rearrangements of N-heterocyclic carbenes of indazoles

**DOI:** 10.3762/bjoc.10.79

**Published:** 2014-04-10

**Authors:** Zong Guan, Jan C Namyslo, Martin H H Drafz, Martin Nieger, Andreas Schmidt

**Affiliations:** 1 Clausthal University of Technology, Institute of Organic Chemistry, Leibnizstrasse 6, D-38678 Clausthal-Zellerfeld, Germany; 2 University of Helsinki, Laboratory of Inorganic Chemistry, Department of Chemistry, P.O. Box 55 (A.I. Virtasen aukio 1), FIN-00014 University of Helsinki, Finland

**Keywords:** *E*/*Z* isomerism, indazol-3-ylidene, mesomeric betaine, pyrazole, quinazoline, Rh complex

## Abstract

Deprotonation of indazolium salts at low temperatures gives N-heterocyclic carbenes of indazoles (indazol-3-ylidenes) which can be trapped as rhodium complexes (X-ray analysis). In the absence of Rh, the indazol-3-ylidenes spontaneously dimerize under ring cleavage of one of the N,N-bonds and ring closure to an indazole–indole spiro compound which possesses an exocyclic imine group. The *E*/*Z* isomers of the imines can be separated by column chromatography when methanol is used as eluent. We present results of a single crystal X-ray analysis of one of the *E*-isomers, which equilibrate in solution as well as in the solid state. Heating of the indazole–indole spiro compounds results in the formation of quinazolines by a ring-cleavage/ring-closure sequence (X-ray analysis). Results of DFT calculations are presented.

## Introduction

**Scheme 1:**
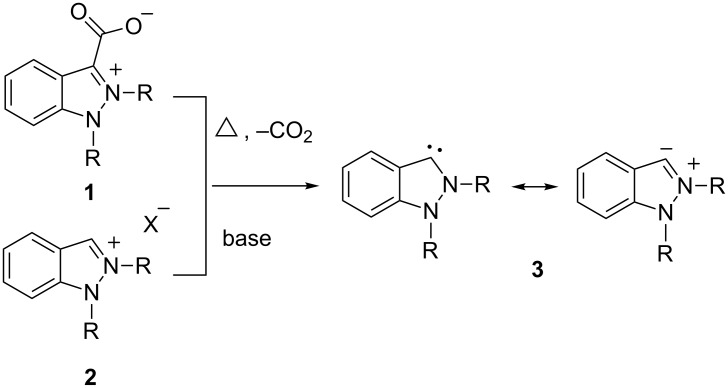
Generation of indazol-3-ylidenes.

As a result of their biochemical and pharmacological significance, there has been a considerably growing interest in indazoles in recent years, which is reflected in several book chapters and review articles dealing with syntheses [1–4], synthetic potentials [4], and biological activities [4–5] of this ring system. In view of the rapid development of the class of N-heterocyclic carbenes (NHC) [6–12] it is not unexpected, that attention was also directed towards the NHCs of indazole which have been generated and applied in heterocyclic synthesis (vide infra) as well as in complex chemistry [13]. Undoubtedly the N-heterocyclic carbenes of imidazole, imidazoline and the triazoles play the most important roles as ligands in metal-organic chemistry [14] or as organocatalysts [15–16]. The N-heterocyclic carbenes of indazole (and pyrazole [17–18]), however, have a chemistry of their own which set them apart from the NHCs of the aforementioned ring systems. Portions of that field have been covered in recent review articles [18–19]. The N-heterocyclic carbene of indazole **3** has been generated by thermal decarboxylation of indazolium-3-carboxylates **1** [20] which belong to the class of pseudo-cross-conjugated heterocyclic mesomeric betaines (Scheme 1). Its properties have been calculated [20–21] and examined by means of vibrational spectroscopy [21]. It was shown that pseudo-cross-conjugated mesomeric betaines decarboxylate readily in the absence of stabilizing effects such as hydrogen bonds to protic solvents or water of crystallization [18–19]. Thus, the Gibbs free energy difference for the decarboxylation of 1,2-dimethylindazolium-3-carboxylate under standard conditions (25 °C, 1 atm) was found to be 3.4 kcal/mol [20]. Alternatively, indazolium salts **2** can be deprotonated by various bases to give indazol-3-ylidenes **3** [22].

**Scheme 2:**
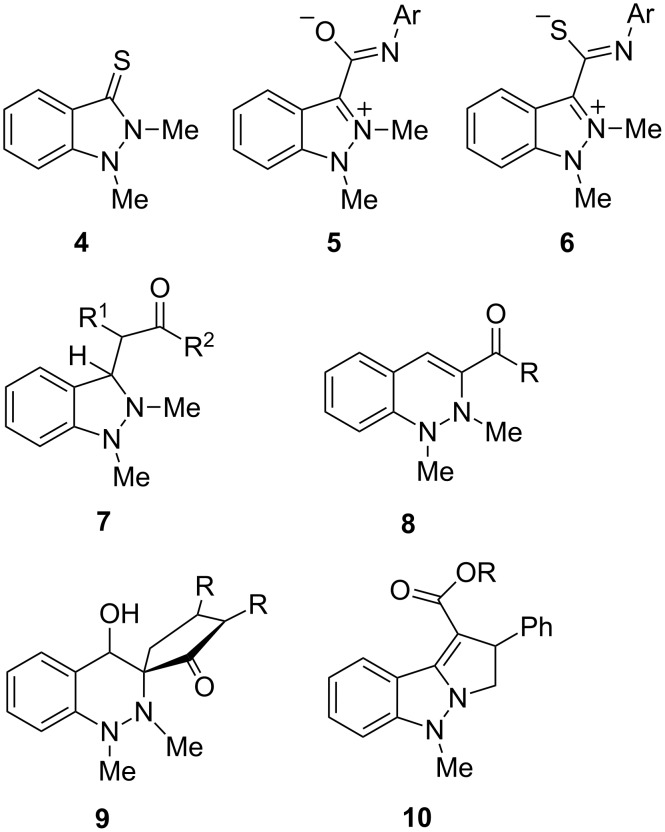
Reaction products of indazol-3-ylidenes in heterocycle synthesis.

The chemistry of indazol-3-ylidene, which is a singlet carbene, is due to the considerable donor strength of the carbene atom which has a calculated Mulliken charge of 0.009 [20]. Moreover, the ability to cleave the N–N single bond, which was calculated to have a bond length of 144.6 pm in 1,2-dimethylindazol-3-ylidene [20] (N–N_1_*_H_*_-indazole_ = 138.4 pm [3]) and a stretching force constant of 4.23 mdyn Å^−1^ [21], opens the access to several heterocyclic transformation products (vide infra). Finally, the synthetic potential is governed by the electrophilic properties of the iminium group of indazolium salts which result from protonation of the carbene. As a consequence, the synthetic potential of indazol-3-ylidene not only strongly depends on the choice of potential reaction partners, but also on its substitution pattern and the reaction conditions. As examples, when N1 is substituted with a methyl group, the carbene can be trapped by elemental sulfur, isocyanates, and isothiocyanates which form indazolethione **4**, indazolium-3-amidate **5**, and indazolium-3-thioamidate **6**, respectively [23] (Scheme 2). Aliphatic ketones surprisingly give stable 1:1 adducts **7** [20,24]. α-Bromo acetophenones induce an unexpected ring enlargement to cinnolines **8** [25]. New ring systems such as **9** [25] and **10** [26] were prepared on treatment of indazol-3-ylidene with acetylenes.

**Scheme 3:**
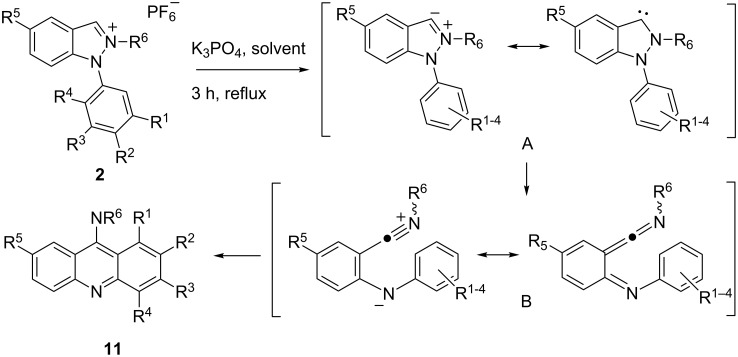
Syntheses of acridines from indazol-3-ylidenes.

Indazol-3-ylidenes which possess an aryl ring at N1 rearrange to give substituted acridines by a ring-cleavage/pericyclic ring-closure reaction sequence (**2**→**A**→**B**→**11**) (Scheme 3). It proved to be advantageous to start these rearrangements from indazolium salts which are readily available by copper-catalyzed aryl couplings or Buchwald–Hartwig reactions [22]. Pyrazol-3-ylidenes rearrange similarly to quinolines [17].

We report here on two unexpected rearrangements of indazol-3-ylidene, and trapping reactions of the N-heterocyclic carbene with rhodium.

## Results and Discussion

**Scheme 4:**
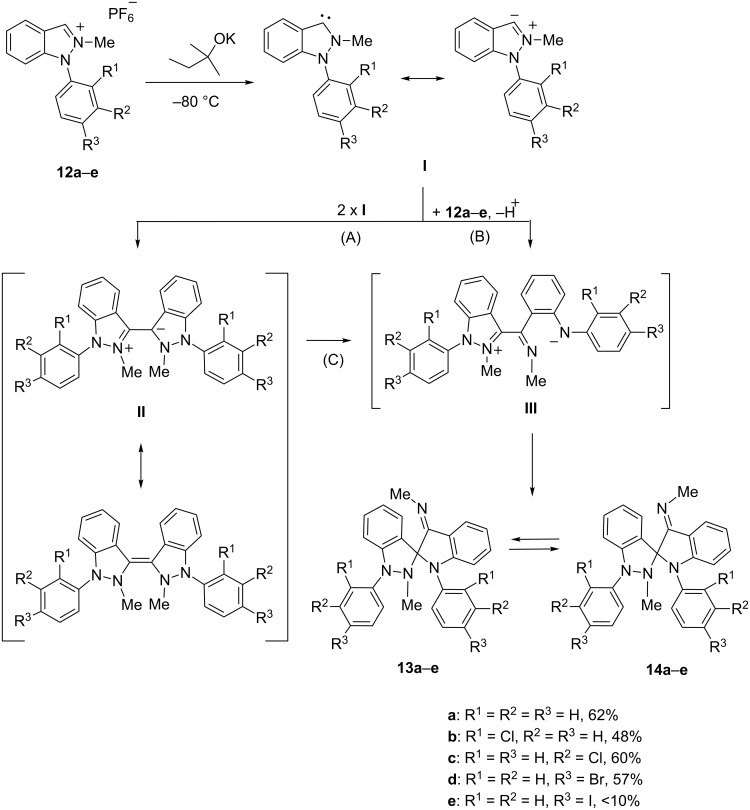
Dimerisation of indazol-3-ylidenes to spiro compounds.

On trying to deprotonate the indazolium salts **12a–e** with potassium 2-methylbutan-2-olate in anhydrous dichloromethane at −80 °C to the N-heterocyclic carbene **I** in the absence of trapping reagents we unexpectedly obtained a mixture of two compounds which are in equilibrium, when the reaction was allowed to warm to room temperature (Scheme 4). From the NMR spectra of the mixture it was apparent that neither the 3,3'-biindazolylidene **II** nor its *trans* isomer had formed, as signals between δ = 91.7 ppm and 94.0 ppm were detected by ^13^C NMR spectroscopy which are neither in agreement with structure **II** nor with its *trans* isomer. Mass spectrometric examinations, however, clearly showed the peaks of dimerized species of the carbene **I**, because the molecular peaks correspond to twice the mass of the salts **12a**–**e** minus two hydrogen atoms, respectively. We were able to separate the mixtures of the reactions of **12a**,**c**,**d** by column chromatography employing methanol as eluent, and to characterize the separated species. The species in equilibrium proved to be spiro[indazole-3,2'-indolines], the exocyclic imine groups of which form *Z*- and *E*-isomers **13a**–**e** and **14a**–**e**, respectively. The equilibration of **14b** is too fast so that a separation of a pure sample failed. The iodo derivative **13e** gave low yields and despite of intense efforts we were not able to obtain the compounds in pure form. Solutions of pure samples of the *E*/*Z*-isomers **13a–d** and **14a**,**c**,**d**, respectively, equilibrated slowly in chloroform solutions to give stable ratios after several days at rt. These results are summarized in Table 1.

**Table 1:** Equilibrium between **13a–d** and **14a–d** in chloroform.

Compounds	Ratio^a^	Time (d)

**13a/14a**	10 : 7	12
**13b/14b**	5 : 1	2
**13c/14c**	1 : 1	18
**13d/14d**	10 : 9	9

^a^Ratios determined by ^1^H NMR spectroscopy in CDCl_3_ at rt.

**Figure 1:**
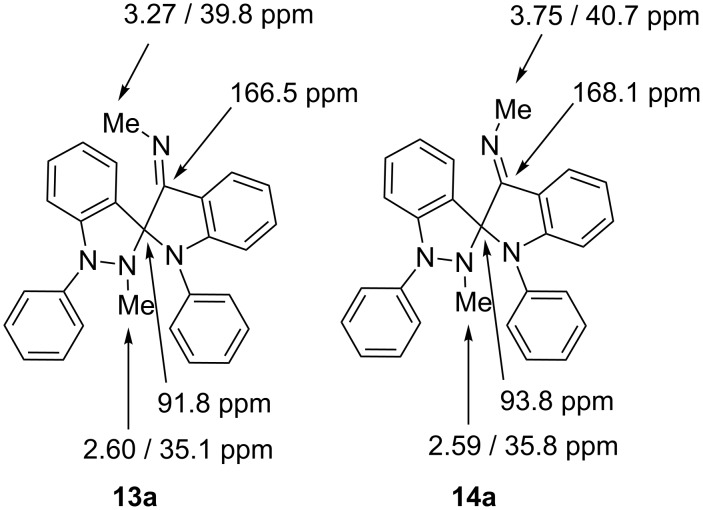
Diagnostic ^1^H and ^13^C NMR chemical shifts of the *Z* and *E* configuration isomer in CDCl_3_.

The isomers **13a–e** and **14a–e** gave identical IR spectra, but the NMR spectra differ considerably (Figure 1). In the NMR spectra the *Z*-isomers **13a–e** show two distinct methyl groups at δ = 3.27 ppm and δ = 2.60 ppm in CDCl_3_, respectively, as well as two different phenyl rings. The methyl groups of the isomeric spiro compounds **14a**,**c**,**d** appear at δ = 3.74 ppm and – as overlapped signal – at δ = 2.60 ppm in the ^1^H NMR spectra measured in CDCl_3_. On conversion of the exocyclic imine groups from the *Z*- into the *E*-configuration (**13** → **14**), the methyl groups move from a position above the plane of the indazole ring into a position in the plane of the indole ring. This change from vertical to horizontal interactions of aromatics on the methyl groups explains the considerable downfield shift of their ^1^H NMR resonance frequencies on *Z*→*E* isomerisation. The following scheme presents diagnostic peak assignments of the ^1^H and ^13^C NMR spectra, taken at 400 and 100 MHz in CDCl_3_, respectively.

According to DFT calculations the conversion of **13a** to **14a** requires an activation energy of Δ*G*^#^ = +79 kJ/mol (Δ*E* = +89 kJ/mol) while the two species do not differ in energy (Δ*E* < 1 kJ/mol). In comparison the inversions of the two nitrogen atoms within the indazole ring (vide infra) require less than 50 kJ/mol. Transition with such low activation energies are not inhibited at room temperature, so the last mentioned inversions are not observable in NMR spectra at standard temperature conditions.

**Figure 2:**
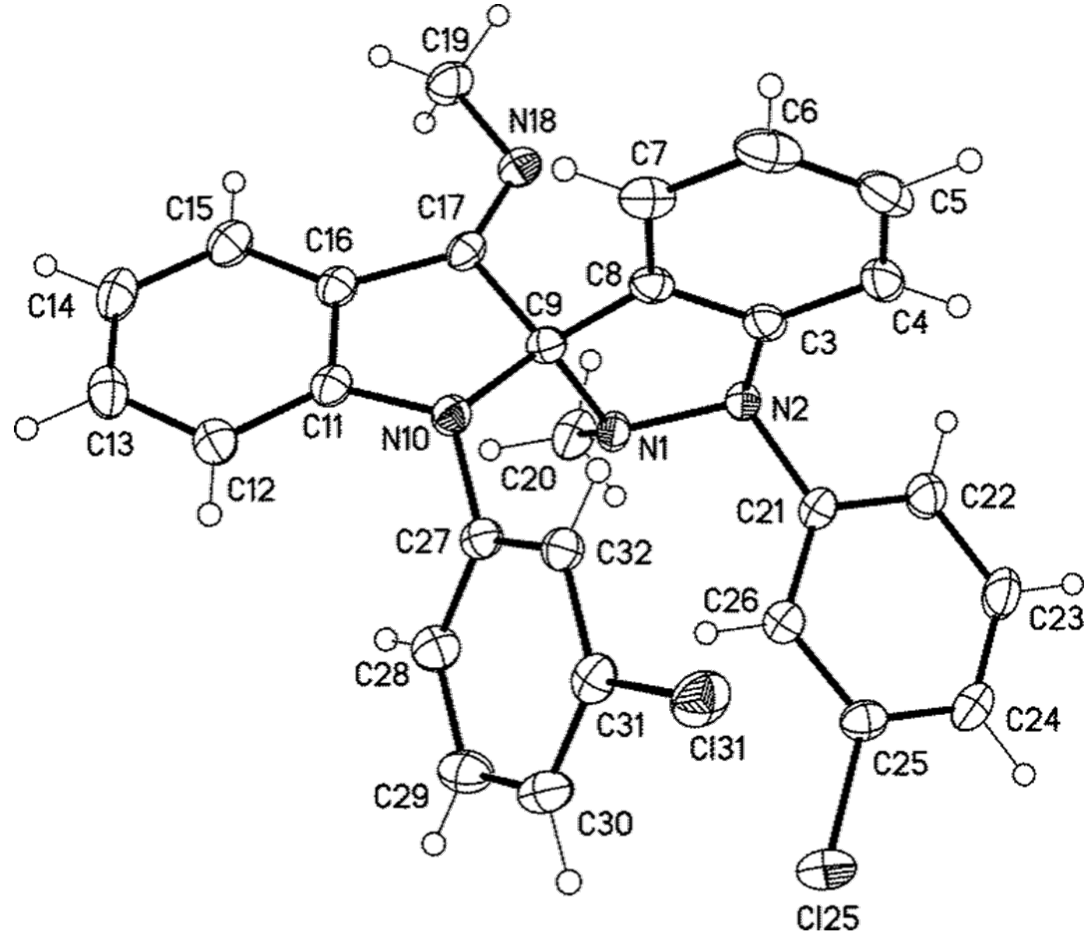
Molecular structure of **14c**, displacement parameters are drawn at 50% probability level.

The spiro compound **14c** crystallized from a saturated solution in *n*-hexane so that we were able to perform a single crystal X-ray analysis. The compound crystallized monoclinic. As expected, neither the pyrrole ring nor the pyrazole ring is planar as evidenced by the dihedral angles C9–N10–C11–C16 = −159.47(12)° and C9–N1–N2–C3 = 15.22(13)° (crystallographic numbering; Figure 2). The pyramidalization of the nitrogen atoms N1 and N10 cause an *anti* conformation of the methyl group attached to N1 and the 3-chlorophenyl ring attached to N10. The latter is twisted with respect to the indole moiety [C11–N10–C27–C28 = 41.85(18)°]. As N2 is also pyramidalized, the methyl group at N1 and the 3-chlorophenyl ring at N2 adopt an *anti* conformation as well. The exocyclic imine has a bond length of 127.13(17) pm and this value corresponds to a typical C_sp_^2^=N imine bond. Correspondingly, the dihedral angle C16–C17–N18–C19 is only −1.3(2)°. The dihedral angle C15–C16–C17–N18, however, is 6.3(3)° so that the imine group is slightly twisted out of the plane of the indole´s phenyl ring. In the single crystal of **14c**, the imine adopts an *E* configuration as already predicted by the NMR investigations.

The mechanism of this new rearrangement can be either postulated by dimerization of the N-heterocyclic carbene **I** to give **II** followed by ring-cleavage of one of the indazole rings to **III** (pathways **A** and **C**, Scheme 4), or as nucleophilic attack of the carbene **I** to the iminium group of the salts **12a–e** followed by ring-cleavage and deprotonation to give betaine **III** (pathway **B**). As a matter of fact, cross-experiments between **13a** and the chlorophenyl derivative **13b** failed in refluxing THF for 4 h as well as in refluxing chloroform under irradiation for 8 h, as no monochloro compound was detectable. Obviously, once the dimerized species is formed under these conditions, there is no equilibrium between the free N-heterocyclic carbene **I** and its dimers **II** or **III**. A closer inspection of the structures reveals that the intermediary betaines **III** are representatives of the aforementioned class of pseudo-cross-conjugated heterocyclic mesomeric betaines. Thus the intermediates **III** are related to indazolium-3-carboxylates, -amidates, and -thioamidates shown in Scheme 1 and Scheme 2. The neutralization of the charges in cross-conjugated as well as in pseudo-cross-conjugated mesomeric betaines by inter- or intramolecular cycloadditions are typical reactions [18–19].

**Scheme 5:**
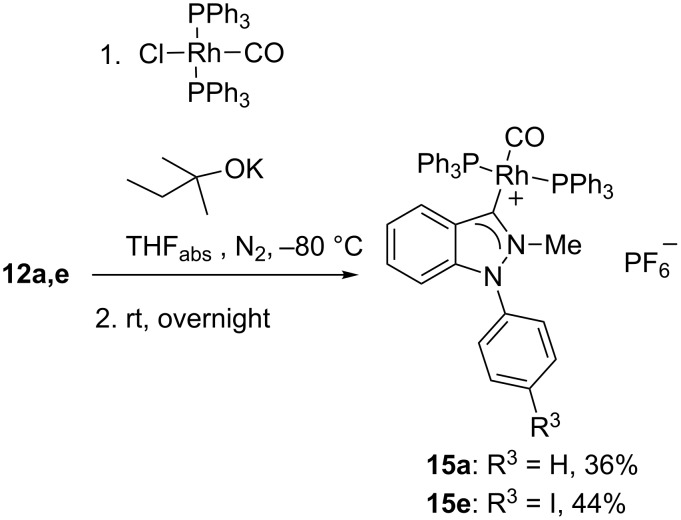
Rhodium complex formation.

To prove the initial formation of an N-heterocyclic carbene in this reaction we tried trapping reactions starting from **12a** and **12e** with carbonylbis(triphenylphosphine)rhodium(I) chloride under otherwise unchanged reaction conditions. Indeed, stable complexes were formed as yellow crystals in either case which were fully characterized (Scheme 5). The carbene atom of **15e** was detected at 191.4 ppm by ^13^C NMR spectroscopy. A comparison of the stretching frequencies of the CO ligands (**15a**: 2009 cm^−1^, **15e**: 1994 cm^−1^) indicated very strong donor strengths of these indazol-3-ylidenes as already postulated earlier on comparing different stretching frequencies of selected 5-membered NHCs [27] or ^13^C NMR resonance frequencies of several palladium carbene complexes [28].

**Figure 3:**
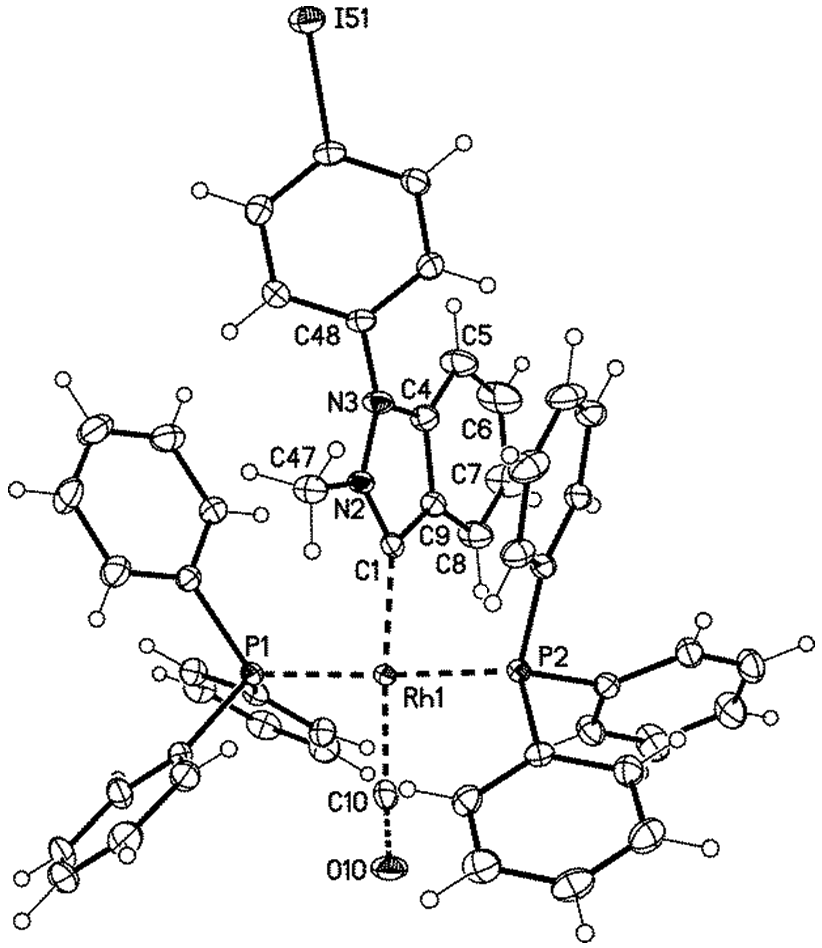
Structure of the cation of **15e**, displacement parameters are drawn at 50% probability level.

We were able to obtain single crystals of **15e** to perform an X-ray analysis (Figure 3). Suitable single crystals were obtained by slow evaporation of a concentrated solution in dichloromethane/iPrOH. The complex crystallized monoclinic. The Rh–C_carbene_ and Rh–C_CO_ bond lengths [Rh1–C1 and Rh1–C10; crystallographic numbering] were determined to be 206.0(2) pm and 186.3(3) pm, respectively. The *p*-iodophenyl substituent is twisted by −50.7(4)° from the plane of the indazole ring [C4–N2–C1–C53].

**Scheme 6:**
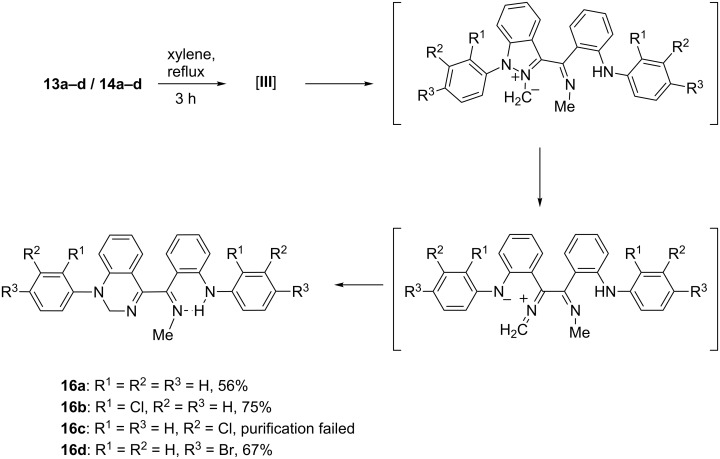
Rearrangement of the spiro compounds on heating.

Another type of rearrangement occurred on heating the dimerized carbenes **13a–d**/**14a–d** in xylene, as the substituted quinazolines **16a–d** were isolated in reasonable yields (Scheme 6). The mechanism can be rationalized by formation of an ylide by 1,7-*H*-shift from the mesomeric betaine **III**, followed by ring cleavage of the indazole ring and subsequent ring-closure of the resulting 1,6-dipole to give the quinazolines **16a–d**.

**Figure 4:**
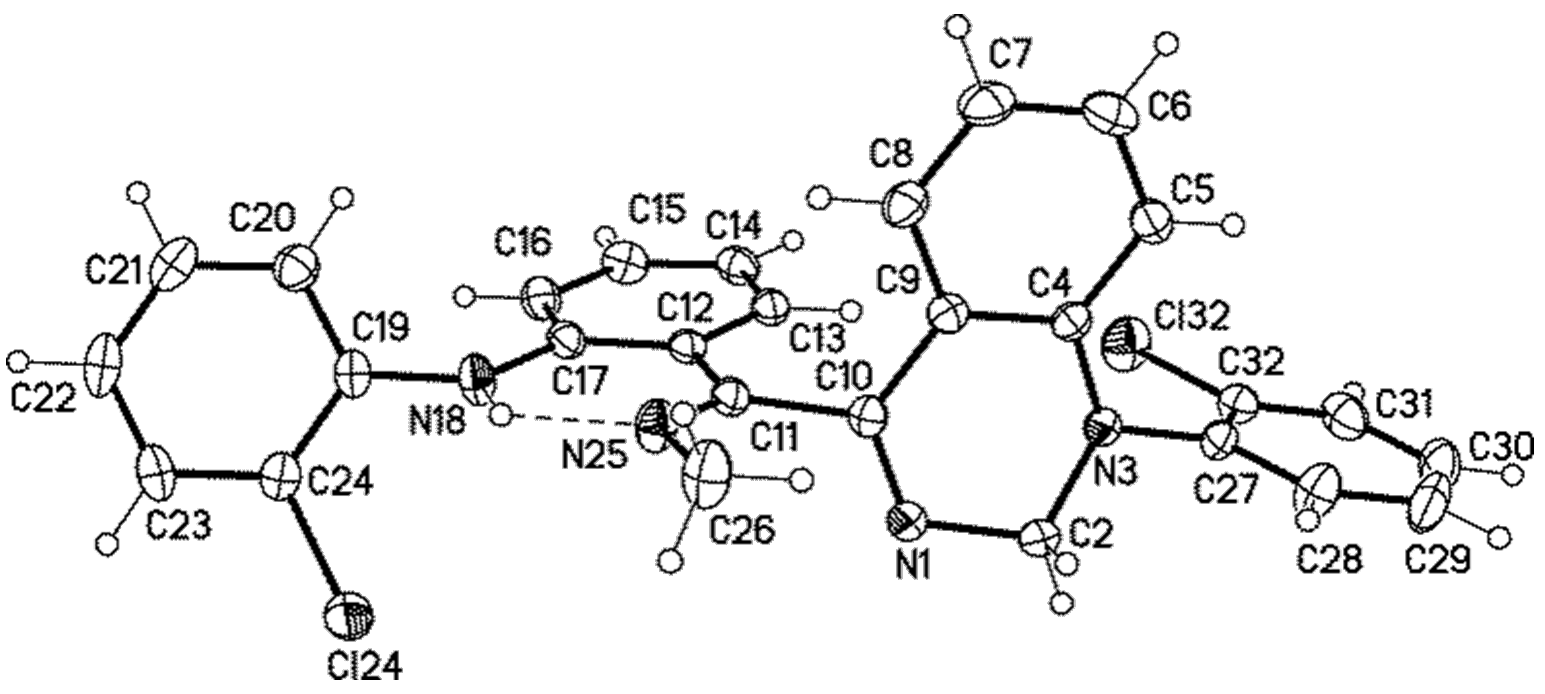
Molecular structure of **16b**, displacement parameters are drawn at 50% probability level.

Single crystals of **16b** suitable for an X-ray analysis were obtained by slow evaporation of a saturated solution in methanol. This compound crystallized monoclinic. A molecular structure is shown in Figure 4. In the single crystal the NH group of the aniline (N18–H) forms a hydrogen bond to the imine group (N25) so that an almost planar six-membered ring is formed. The dihedral angle C11–C12–C17–N18 (crystallographic numbering) was determined to be 0.5(2)°. This six-membered ring is almost perpendicularly twisted in relation to the quinazoline ring [N1–C10–C11–C12 = −92.65(17)°].

## Conclusion

The N-heterocyclic carbene of indazole, indazol-3-ylidene, displays a chemistry of its own which differs from the chemistry of N-heterocyclic carbenes of other ring systems. At low temperatures it can be trapped as a rhodium complex. Without trapping reagents it dimerizes under ring-cleavage to form two isomeric spiro compounds possessing *E*- and *Z*-configurated methylimine groups. Heating of the carbene dimer results in the formation of novel quinazolines.

## Experimental

General considerations: All reactions for the dimerisation and the rearrangement were carried out under an atmosphere of nitrogen in oven-dried glassware. Flash-chromatography was performed with silica gel 60 (0.040–0.063 mm). Nuclear magnetic resonance (NMR) spectra were obtained with a Bruker Avance 400 and Bruker Avance III 600 MHz. ^1^H NMR spectra were recorded at 400 MHz or 600 MHz. ^13^C NMR spectra were recorded at 100 MHz or 150 MHz, with the solvent peak or tetramethylsilane used as the internal reference. Multiplicities are described by using the following abbreviations: s = singlet, d = doublet, t = triplet, q = quartet, and m = multiplet. FTIR spectra were obtained on a Bruker Vector 22 in the range of 400 to 4000 cm^−1^. The mass spectra were measured with a Varian 320 MS Triple Quad GC–MS/MS with a Varian 450-GC. The electrospray ionization mass spectra (ESIMS) were measured with an Agilent LCMSD series HP 1100 with APIES. Melting points are uncorrected and were determined in an apparatus according to Dr. Tottoli (Büchi). The HRMS spectra were measured on a Bruker Daltonik Tesla-Fourier transform-ion cyclotron resonance mass spectrometer with electrospray ionisation. Yields are not optimized. Compounds **12a**, **12b**, **12c** and **12e** were described in an earlier publication [22]. 1-(4-Bromophenyl)-1*H*-indazole was prepared by the method B which we described earlier [22] and was isolated in better yield (85%) than in the literature (40%) [29]. All density-functional theory (DFT)-calculations were carried out by using the Jaguar 7.7.107 software running on Linux 2.6.18-238.el5 SMP (x86_64) on two AMD Phenom II X6 1090T processor workstations (Beowulf-cluster) parallelized with OpenMPI 1.3.4. MM2 optimized structures were used as starting geometries. Complete geometry optimizations were carried out on the implemented LACVP* (Hay–Wadt effective core potential (ECP) basis on heavy atoms, N31G6* for all other atoms) basis set and with the B3LYP density functional. All calculated structures were proven to be true minima by the absence of imaginary frequencies or transition states by the occurrence of one negative frequency. Plots were obtained using Maestro 9.1.207, the graphical interface of Jaguar. Inversion barriers have been calculated fully relaxed, fixating one torsion angle around the inverted center, and optimizing all remaining degrees of freedom. Torsion angles were modified in steps of 10°.

Thermodynamic corrections were estimated from unscaled frequencies, using standard formulae in the ideal gas harmonic oscillator approximation as implemented in Jaguar, and refer to a standard state of 298.15 K and 1 mol/dm^3^ concentration.

### Crystal structure determinations of **14c**, **15e**, **16b**

The single-crystal X-ray diffraction study was carried out on a Bruker-Nonius Kappa-CCD at 123(2) K using MoKα radiation (λ = 0.71073 Å). Direct Methods (SHELXS-97) [30] were used for structure solution and refinement was carried out using SHELXL-2013 [30] (full-matrix least-squares on *F^2^*). Hydrogen atoms were localized by difference electron density determination and refined using a riding model (H(N) free). Semi-empirical absorption corrections were applied. In **15e** one of the 3 solvent molecules CH_2_Cl_2_ is disordered. For more information see the Supporting Information File 1.

**14c**: yellow, C_25_H_22_Cl_2_N_4_, *M* = 485.39, crystal size 0.45 × 0.25 × 0.15 mm, monoclinic, space group *P*2_1_/*c* (no. 14): *a* = 13.522(1) Å, *b* = 14.131(1) Å, *c* = 12.306(1) Å, β = 94.16(1)°, *V* = 2345.2(3) Å^3^, *Z* = 4, ρ(calc) = 1.375 Mg m^−3^, *F*(000) = 1008, *µ* = 0.302 mm^−1^, 38867 reflections (2θ_max_ = 55°), 5367 unique [R_int_ = 0.028], 309 parameters, *R*1 (for 4546 *I* > 2σ(*I*)) = 0.032, *wR2 (all data)* = 0.086, GOOF = 1.05, largest diff. peak and hole 0.339 / −0.294 e Å^−3^.

**15e**: yellow, C_51_H_41_IN_2_OP_2_Rh^+^ –PF_6_^−^ · 3CH_2_Cl_2_, *M* = 1389.36, crystal size 0.35 × 0.25 × 0.15 mm, monoclinic, space group *P*2_1_/*n* (no. 14): *a* = 14.314(1) Å, *b* = 26.691(2) Å, *c* = 15.045(1) Å, β = 99.43(1)°, *V* = 5670.3(7) Å^3^, *Z* = 4, ρ(calc) = 1.627 Mg m^−3^, *F*(000) = 2768, µ = 1.273 mm^−1^, 91339 reflections (2θ_max_ = 55°), 12976 unique [R_int_ = 0.021], 685 parameters, 70 restraints, *R*1 (for 11838 *I* > 2σ(*I*)) = 0.032, *wR2 (all data)* = 0.086, GOOF = 1.09, largest diff. peak and hole 1.473/−1.377 e Å^−3^.

**16b**: colourless, C_28_H_22_Cl_2_N_4_, *M* = 485.39, crystal size 0.30 × 0.24 × 0.06 mm, monoclinic, space group *P*2_1_/*n* (no. 14): *a* = 14.690(1) Å, *b* = 7.488(1) Å, *c* = 21.484(2) Å, β = 90.75(1)°, *V* = 2363.0(4) Å^3^, *Z* = 4, ρ(calc) = 1.364 Mg m^−3^, *F*(000) = 1008, *µ* = 0.300 mm^−1^, 36379 reflections (2θ_max_ = 55°), 5408 unique [R_int_ = 0.034], 311 parameters, 1 restraints, *R*1 (for 4414 *I* > 2σ(*I*)) = 0.040, *wR2 (all data)* = 0.093, GOOF = 1.05, largest diff. peak and hole 0.325/−0.278 e Å^−3^.

Crystallographic data (excluding structure factors) for the structures reported in this work have been deposited with the Cambridge Crystallographic Data Centre as supplementary publication no. CCDC-972482 (**14c**), CCDC-972481 (**15e**), and CCDC-972483 (**16b**). Copies of the data can be obtained free of charge on application to The Director, CCDC, 12 Union Road, Cambridge DB2 1EZ, UK (Fax: int.code+(1223)336-033; e-mail: deposit@ccdc.cam.ac.uk).

### General procedure for the preparation of the indazole carbene dimers **13**/**14**

A solution of 2.0 mmol of the indazolium salts **12a–e** in 20 mL of dichloromethane was cooled to −80 °C. Then 1.2 mL of a 2 M solution of potassium 2-methylbutan-2-olate in THF was added dropwise within 30 minutes. The reaction mixture was then evaporated to dryness and extracted twice with 20 mL of petroleum ether, respectively. After evaporation of the solvent in vacuo the crude reaction product was purified by flash column chromatography (silica gel; methanol) and dried in vacuo. The isomers **13** and **14** have *R*_f_ values of approximately 0.4 and 0.2 on silica gel in MeOH, respectively.

### (*Z*)-*N*-(2-Methyl-1,1'-diphenyl-1,2-dihydrospiro[indazole-3,2'-indolin]-3'-ylidene)methanamine (**13a**)

Yield: 152 mg (37%) of a yellow solid; mp 74–75 °C; ^1^H NMR (400 MHz, CDCl_3_) δ 7.68 (dd, *J* = 7.5, 0.6 Hz, Ar-H, 1H), 7.29–7.25 (m, 2H, Ar-H), 7.23–7.06 (m, 9H, Ar-H), 6.96 (dd, *J* = 8.6, 1.2 Hz, 2H, Ar-H), 6.89–6.83 (m, 2H, Ar-H), 6.71 (d, *J* = 8.1 Hz, 1H, Ar-H), 6.58 (d, *J* = 8.1 Hz, 1H, Ar-H), 3.27 (s, 3H, CH_3_), 2.60 (s, 3H, CH_3_) ppm; ^13^C NMR (100 MHz, CDCl_3_) δ 166.5, 153.5, 149.0, 145.8, 140.7, 133.0, 129.4, 129.1, 129.0, 127.6, 125.8, 125.7, 124.9, 124.5, 123.7, 123.5, 122.5, 121.9, 119.2, 110.1, 109.5, 91.8, 39.8, 35.1 ppm; IR (ATR): 3051, 2952, 2891, 2855, 1655, 1605, 1590, 1450, 1354, 1312, 1271, 1191, 1151, 1000, 922, 856, 697, 681, 480 cm^−1^; ESIMS: *m*/*z* (%) = 417 [M + H^+^]; HRESIMS: C_28_H_25_N_4_ calcd for 417.2079; found: 417.2075.

### (*E*)-*N*-(2-Methyl-1,1'-diphenyl-1,2-dihydrospiro[indazole-3,2'-indolin]-3'-ylidene)methanamine **14a**

Yield: 106 mg (25%) of a yellow solid; mp 79–80 °C; ^1^H NMR (400 MHz, CDCl_3_) δ 7.81 (d, *J* = 7.5 Hz, 1H, Ar-H), 7.30–7.27 (m, 1H, Ar-H), 7.25–7.21 (m, 2H, Ar-H), 7.19–7.13 (m, 6H, Ar-H), 7.10–7.06 (m, 2H, Ar-H), 6.97–6.95 (m, 2H, Ar-H), 6.91 (ddd, J = 7.4, 7.4, 0.8 Hz, 1H, Ar-H), 6.83–6.77 (m, 2H, Ar-H), 6.62 (d, *J* = 8.0 Hz, 1H, Ar-H), 3.75 (s, 3H, CH_3_), 2.59 (s, 3H, CH_3_) ppm; ^13^C NMR (100 MHz, CDCl_3_) δ 168.1, 155.3, 149.1, 147.0, 140.2, 133.2, 129.4, 129.0, 128.9, 128.8, 128.1, 127.7, 126.1, 125.3, 124.3, 123.8, 121.8, 118.3, 118.1, 111.1, 109.6, 93.8, 40.7, 35.8 ppm; IR (ATR): 3034, 2950, 2888, 2858, 1655, 1605, 1590, 1450, 1355, 1312, 1272, 1192, 1152, 999, 921, 857, 697, 681, 480 cm^−1^; ESIMS: *m*/*z* (%) = 417 [M + H^+^]; HRESIMS: C_28_H_25_N_4_ calcd for 417.2079; found: 417.2075.

### General procedure for the preparation of the rhodium complexes **15**

A solution of 0.2 mmol of the indazolium salts **12a** and **12e**, respectively, and 0.2 mmol of carbonylbis(triphenylphosphine)rhodium(I) chloride in 20 mL of THF was cooled to −80 °C. Then, 0.1 mL of a 2 M solution of potassium 2-methylbutan-2-olate in THF was added dropwise. The reaction mixture was then stirred overnight at room temperature. Yellow solids formed which were filtered off, washed with 2 mL of ethylacetate, and dried in vacuo.

### Carbonyl-bis(triphenylphosphine)(2-methyl-1-phenyl-1*H*-indazole-3-ylidene)rhodium(I) hexafluorophosphate (**15a**)

Yield: 73 mg (36%) of yellow crystals; dec. 240 °C; ^1^H NMR (400 MHz, DMSO-*d*_6_) δ 7.67 (d, *J* = 8.3 Hz, 1H), 7.62–7.60 (m, 3H), 7.56–7.39 (m, 31H), 7.04–6.97 (m, 3H), 6.77 (d, *J* = 8.3 Hz, 1H), 3.23 (s, 3H) ppm; ^13^C NMR (150 MHz, DMSO-*d*_6_) δ 139.2, 133.5 (t, *J* = 6.0 Hz), 132.4 (t, *J* = 23.0 Hz), 132.3, 132.1, 131.0, 130.9, 130.3, 128.9 (t, *J* = 4.3 Hz), 128.7, 127.7, 126.2, 122.2, 109.6, 40.2 ppm; IR (ATR): 2009, 1498, 1479, 1436, 1308, 1095, 861, 833, 741, 641, 557, 498 cm^−1^; ESIMS: *m*/*z* (%) = 863 [M ^+^]; HRESIMS: C_51_H_42_N_2_OP_2_Rh calcd for 863.1827; found: 863.1827.

### General procedure for the rearrangements of the indazole carbene dimers to **16**

A solution of 1.0 mmol of the dimers of the indazole carbenes **13a/13b/13d** in 20 mL of xylene was stirred at reflux temperature for 3 hours. After the solvent was distilled off in vacuo, the crude product was purified by flash column chromatography (petroleum ether: ethyl acetate = 3:1) and dried in vacuo.

### 2-((Methylimino)(1-phenyl-1,2-dihydroquinazolin-4-yl)methyl)-*N*-phenylaniline **16a**

Yield 233 mg (56%) of yellow crystals; mp 146–147 °C. ^1^H NMR (400 MHz, CDCl_3_) δ 11.74 (bs, 1H, NH), 7.46–7.42 (m, 2H, Ar-H), 7.38–7.28 (m, 8H, Ar-H), 7.24–7.16 (m, 3H, Ar-H), 7.11–7.05 (m, 2H, Ar-H), 6.97 (d, *J* = 8.3 Hz, 1H, Ar-H), 6.77 (t, *J* = 7.6 Hz, 1H, Ar-H), 6.65 (t, *J* = 7.6 Hz, 1H, Ar-H), 5.51 (s, 2H, CH_2_), 3.41 (s, 3H, CH_3_) ppm; ^13^C NMR (100 MHz, CDCl_3_) δ 169.4, 164.6, 146.7, 145.1, 144.1, 141.8, 133.3, 132.1, 129.7, 129.4, 127.3, 124.9, 123.5, 122.8, 122.2, 120.1, 119.2, 118.6, 116.9, 116.0, 114.0, 66.6, 40.1 ppm; IR (ATR): 3025, 2968, 2856, 1714, 1589, 1524, 1482, 1448, 1319, 1174, 740, 693, 638, 518 cm^−1^. ESIMS: *m*/*z* (%) = 417 [M + H^+^]. HRESIMS: C_28_H_25_N_4_ calcd for 417.2079; found: 417.2080.

## Supporting Information

File 1 Synthetic procedures, characterization data, X-ray data and NMR spectra.
