# Supplementary material for: Dimerisation, rhodium complex formation and rearrangements of N-heterocyclic carbenes of indazoles
Source: Beilstein J Org Chem. 2014 Apr 10;10:832–40. doi: 10.3762/bjoc.10.79 (PMC3999838; doi:10.3762/bjoc.10.79)
Supplement: File 1 — Synthetic procedures, characterization data, X-ray data and NMR spectra. [file Beilstein_J_Org_Chem-10-832-s001.pdf]

Supporting Information

for

Dimerisation, rhodium complex formation and rearrangements of N-heterocyclic carbenes of indazoles

Zong Guan[1], Jan C. Namyslo[1], Martin H. H. Drafz[1], Martin Nieger[2] and Andreas Schmidt*[1]

Address: [1]Clausthal University of Technology, Institute of Organic Chemistry, Leibnizstrasse 6, D-38678 Clausthal-Zellerfeld, Germany and [2]University of Helsinki, Laboratory of Inorganic Chemistry, Department of Chemistry, P.O. Box 55 (A.I. Virtasen aukio 1), FIN-00014 University of Helsinki, Finland

Email: Andreas Schmidt - schmidt@ioc.tu-clausthal.de

*Corresponding author

Synthetic procedures, characterization data, X-ray data and NMR spectra.

**Experimental procedures**	**s2–s4**
**Crystal structure determinations**	**s5–s31**
**NMR spectra**	**s31–s44**
**DFT calculations**	**s45–s50**

Experimental procedures

Preparation of 1-(4-bromophenyl)-2-methyl-1*H*-indazolium hexafluorophosphate (12d):

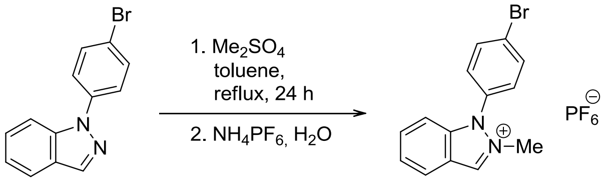

A solution of 1.0 mmol of 1-(4-bromophenyl)-1*H*-indazole[1] in 20 mL of toluene was treated with 4.0 mmol of dimethylsulfate and stirred at reflux temperature for 24 hours, during which time a dark oil formed. After cooling to room temperature the oil was separated from the solvent and dissolved in 20 mL of water and filtered. Then, a solution of 163 mg (1.0 mmol) of ammonium hexafluorophosphate in 1 mL of water was added. Colorless solids formed which were filtered off, recrystallized from water, and dried in vacuo. Yield: 349 mg (81%) of colorless crystals, mp: 117-118 °C. ^{1}H NMR (400 MHz, DMSO-d_6): δ = 9.53 (s, 1H, Ar-H), 8.25 (dt, *J* = 8.6 / 0.8 Hz, 1H, Ar-H), 8.04 - 8.01 (m, 2H, Ar-H), 7.87 (ddd, *J* = 8.6 / 7.0 / 1.0 Hz, 1H, Ar-H), 7.82 - 7.79 (m, 2H, Ar-H), 7.61 (ddd, *J* = 8.4 / 7.0 / 0.8 Hz, 1H, Ar-H), 7.45 (dd, *J* = 8.4 / 1.0Hz, 1H, Ar-H), 4.16 (s, 3H, CH_3) ppm. ^{13}C NMR (100 MHz, DMSO-d_6): δ = 140.7, 135.2, 134.1, 133.8, 131.3, 130.0, 126.2, 125.5, 123.4, 119.3, 111.0, 38.6 ppm. IR (KBr): 3135, 3098, 1629, 1536, 1488, 1213, 1213, 1010, 842, 750, 557 cm^{-1}. ESI-MS (0V): 287 [M^{+}]. HRESIMS: $C_{14}H_{12}N_2Br$ required 287.0184. Found: 287.0180.

(*Z*)-*N*-(1,1'-Bis(2-chlorophenyl)-2-methyl-1,2-dihydrospiro[indazole-3,2'-indolin]-3'-ylidene)methanamine (13b):

Yield: 232 mg (48%) of yellow solid, mp: 96-97 °C. ^{1}H NMR (400 MHz, CDCl3): δ = 7.70 (dd, *J* = 7.5 / 0.6 Hz, 1H, Ar-H), 7.56 (dd, *J* = 8.1 / 1.4 Hz, 1H, Ar-H), 7.40 (dd, *J* = 8.1 / 1.3 Hz, 1H, Ar-H), 7.35 (d, *J* = 7.3 Hz, 1H, Ar-H), 7.29 7.23 (m, 2H, Ar-H), 7.17 (ddd, *J* = 7.9 / 7.2 / 1.3 Hz, 1H, Ar-H), 7.08 (ddd, *J* = 7.4 / 7.6 / 1.6 Hz, 1H, Ar-H), 7.01 (d, *J* = 7.4 Hz, 1H, Ar-H) 6.92 (ddd, *J* = 7.7 / 7.6 / 1.4 Hz, 1H, Ar-H), 6.83 (ddd, *J* = 7.8 / 7.4 / 0.8 Hz, 1H, Ar-H), 6.73 (dd, *J* = 8.1 / 1.5 Hz, 1H, Ar-H), 6.38 (d, *J* = 8.1 Hz, 1H, Ar-H), 6.27 (d, *J* = 8.0 Hz, 1H, Ar-H), 6.22 (d, *J* = 8.0 Hz, 1H, Ar-H), 6.15 (dd, *J* = 8.1 / 1.5 Hz, 1H, Ar-H), 3.23 (s, 3H, Me-H), 2.64 (s, 3H, Me-H) ppm. ^{13}C NMR (100 MHz, $CDCl_3$): δ = 165.9, 152.2, 147.9, 143.9, 136.4, 136.0, 132.9, 132.8, 132.1, 130.5, 129.7, 129.6, 128.3, 128.0, 127.5, 126.9, 126.1, 125.2, 123.8, 122.7, 122.1, 121.3, 118.7, 111.4, 108.2, 92.8, 40.0, 37.3 ppm. IR (ATR): 2954, 2922, 1656, 1604, 1583, 1483, 1462, 1442, 1368, 1313, 1245, 1062, 1028, 931, 770, 739, 724, 665, 603, 446 cm^{-1}. ESIMS: *m/z* (%) = 485 [M+H^{+}]. HRESIMS: $C_{28}H_{23}N_4Cl_2$ required 485.1300. Found: 485.1302.

(*Z*)-*N*-(1,1'-Bis(3-chlorophenyl)-2-methyl-1,2-dihydrospiro[indazole-3,2'-indolin]-3'-ylidene)methanamine (13c):

Yield: 146 mg (30%) of yellow solid, mp: 76-77 °C**.** ^{1}H NMR (400 MHz, $CDCl_3$): δ = 7.70 (d, *J* = 7.4 Hz, 1H, Ar-H), 7.33-7.29 (m, 1H, Ar-H), 7.26 (d, *J* = 8.0 Hz, 1H, Ar-H), 7.17-7.10 (m, 6H, Ar-H), 7.04-7.02 (m, 1H, Ar-H), 6.93-6.89 (m, 4H, Ar-H), 6.77 (d, *J* = 8.0 Hz, 1H, Ar-H), 6.66 (d, *J* = 8.3 Hz, 1H, Ar-H), 3.25 (s, 3H, Me-H), 2.59 (s, 3H, Me-H) ppm. ^{13}C NMR (100 MHz, $CDCl_3$): δ = 165.6, 152.5, 148.3, 146.9, 142.1, 135.0, 134.5,

133.1, 130.1, 129.9, 129.8, 127.1, 126.1, 125.9, 124.9, 123.9 (2 carbons), 123.7, 122.7 (2 carbons), 122.5, 120.0, 110.2, 109.8, 91.7, 39.8, 35.3 ppm. IR (ATR): 3060, 2956, 2891, 2858, 1656, 1585, 1460, 1426, 1389, 1311, 1289, 997, 743, 716, 703, 687, 424 cm^{-1}. ESIMS: *m/z* (%) = 485 [$M+H^+$]. HRESIMS: $C_{28}H_{23}N_4Cl_2$ required 485.1300. Found: 485.1308.

(*Z*)-*N*-(1,1'-Bis(4-bromophenyl)-2-methyl-1,2-dihydrospiro[indazole-3,2'-indolin]-3'-ylidene)methanamine (13d):

Yield: 173 mg (30%) of yellow solid, mp: 101-102 °C. ^1H NMR (400 MHz, $CDCl_3$): δ = 7.68 (dd, *J* = 7.6 / 0.9 Hz, 1H, Ar-H), 7.44-7.40 (m, 2H, Ar-H), 7.34-7.31 (m, 2H, Ar-H), 7.30-7.26 (m, 1H, Ar-H), 7.15-7.10 (m, 2H, Ar-H), 6.95-6.88 (m, 4H, Ar-H), 6.85-6.82 (m, 2H, Ar-H), 6.69 (d, *J* = 8.1 Hz, 1H, Ar-H), 6.57 (d, *J* = 8.1 Hz, 1H, Ar-H), 3.23 (s, 3H, Me-H), 2.55 (s, 3H, Me-H) ppm. ^{13}C NMR (100 MHz, $CDCl_3$): δ = 165.7, 152.9, 148.5, 144.9, 139.9, 133.1, 132.3, 132.2, 129.7, 129.0, 126.0, 124.9, 123.7, 123.6, 122.7, 122.6, 119.8, 119.1, 119.0, 110.3, 109.5, 91.7, 39.8, 35.2 ppm. IR (ATR): 3016, 2951, 2891, 1656, 1604, 1583, 1482, 1463, 1351, 1311, 1193, 1153, 1069, 812, 716, 496 cm^{-1}. ESIMS: *m/z* (%) = 573 [$M+H^+$]. HRESIMS: $C_{28}H_{23}N_4Br_2$ required 573.0289. Found: 573.0292.

(*E*)-*N*-(1,1'-Bis(3-chlorophenyl)-2-methyl-1,2-dihydrospiro[indazole-3,2'-indolin]-3'-ylidene)methanamine (14c):

Yield: 146 mg (30%) of yellow solid, mp: 134-135 °C**.** ^1H NMR (400 MHz, $CDCl_3$): δ = 7.82 (d, *J* = 7.4 Hz, 1H, Ar-H), 7.32 (ddd, *J* = 7.4 / 7.3 / 1.2 Hz, 1H, Ar-H), 7.19-7.11 (m, 6H, Ar-H), 7.06-7.00 (m, 2H, Ar-H), 6.97 (ddd, *J* = 7.3 / 7.2 / 0.8 Hz, 1H, Ar-H), 6.91-6.82 (m, 4H, Ar-H), 6.73 (d, *J* = 8.1 Hz, 1H, Ar-H), 3.75 (s, 3H, Me-H), 2.59 (s, 3H, Me-H) ppm. ^{13}C NMR (100 MHz, $CDCl_3$): δ = 167.2, 154.3, 148.0, 147.9, 141.3, 134.7, 134.5, 133.3, 130.0, 129.7, 129.3, 129.2, 127.7, 127.6, 126.5, 125.7, 125.0, 124.1, 122.6, 122.5, 121.5, 118.9, 118.7, 111.4, 109.9, 94.0, 40.7, 36.8 ppm. IR (ATR): 3052, 2954, 2990, 1655, 1585, 1461, 1311, 1289, 998, 781, 743, 716, 703, 688, 445 cm^{-1}. ESIMS: *m/z* (%) = 485 [$M+H^+$]. HRESIMS: $C_{28}H_{23}N_4Cl_2$ required 485.1300. Found: 485.1308.

(*E*)-*N*-(1,1'-Bis(4-bromophenyl)-2-methyl-1,2-dihydrospiro[indazole-3,2'-indolin]-3'-ylidene)methanamine (14d):

Yield: 155 mg (27%) of yellow solid, mp: 89-90 °C**.** ^1H NMR (400 MHz, $CDCl_3$): δ = 7.81 (d, *J* = 7.7 Hz, 1H, Ar-H), 7.35-7.27 (m, 5H, Ar-H), 7.14-7.11 (m, 2H, Ar-H), 6.96-6.92 (m, 3H, Ar-H), 6.86-6.81 (m, 3H, Ar-H), 6.76 (d, *J* = 8.2 Hz, 1H, Ar-H), 6.64 (d, *J* = 8.1 Hz, 1H, Ar-H), 3.74 (s, 3H, Me-H), 2.56 (s, 3H, Me-H) ppm. ^{13}C NMR (100 MHz, $CDCl_3$): δ = 167.4, 154.6, 148.3, 145.9, 139.2, 133.3, 132.2, 131.9, 129.5, 129.3, 129.2, 127.8, 125.1, 124.1, 122.4, 119.5, 118.8, 118.6, 118.1, 111.4, 109.7, 94.0, 40.8, 36.5 ppm. IR (ATR): 3053, 2951, 2922, 2857, 1896, 1655, 1601, 1461, 1351, 1311, 1068, 1008, 745, 495 cm^{-1}. ESIMS: *m/z* (%) = 573 [$M+H^+$]. HRESIMS: $C_{28}H_{23}N_4Br_2$ required 573.0289. Found: 573.0292.

Carbonyl-bis(triphenylphosphine)(2-methyl-1-(4-iodophenyl)-1*H*-indazole-3-ylidene)rhodium(I) hexafluorophosphate (15e):

Yield: 100 mg (44%) of yellow crystals. mp: 197-198 °C, decomposition. ^1H NMR (400 MHz, $CDCl_3$): δ = 7.97 (d, *J* = 8.6 Hz, 2H), 7.66 (d, *J* = 8.2 Hz, 1H), 7.55-7.39 (m, 31H), 7.02 (dd, *J* = 8.6 / 7.7 Hz, 1H), 6.82-6.77 (m, 3H), 3.25 (s, 3H) ppm. ^{13}C NMR (100 MHz, $CDCl_3$): δ = 191.4, 185.9, 139.2, 139.0, 133.4 (t, *J* = 6.6 Hz), 132.3

(t, J = 23.0 Hz), 132.1, 131.9, 131.0, 129.5, 128.8 (t, J = 4.9 Hz), 128.4, 126.2, 122.3, 109.6, 97.8, 40.2 ppm. IR (ATR): 3053, 1994, 1615, 1571, 1479, 1434, 1305, 1152, 1092, 831, 692, 599 cm^{-1}. ESIMS: *m/z* (%) = 989 [M^+]. HRESIMS: $C_{51}H_{41}N_2OP_2RhI$ required 989.0794. Found: 989.0797.

2-Chloro-*N*-(2-((1-(2-chlorophenyl)-1,2-dihydroquinazolin-4-yl)(methylimino)methyl)phenyl)aniline (16b):

Yield: 364 mg (75%) of yellow crystals, mp: 178-179 °C. ^{1}H NMR (400 MHz, $CDCl_3$): δ = 12.01 (bs, 1H, NH-H), 7.60 (dd, J = 8.2 / 1.6 Hz, 1H, Ar-H), 7.54 (dd, J = 7.8 / 1.2 Hz, 1H, Ar-H), 7.45-7.41 (m, 3H, Ar-H), 7.39-7.35 (m, 2H, Ar-H), 7.31-7.27 (m, 1H, Ar-H), 7.23-7.18 (m, 3H, Ar-H), 7.08 (dd, J = 7.8 / 1.3 Hz, 1H, Ar-H), 6.92 (ddd, J = 7.6 / 7.6 / 1.5 Hz, 1H, Ar-H), 6.75-6.70 (m, 2H, Ar-H), 6.37 (d, J = 8.2 Hz, 1H, Ar-H), 5.46 (s, 2H, CH_2), 3.45 (s, 3H, CH_3) ppm. ^{13}C NMR (100 MHz, $CDCl_3$): δ = 168.6, 164.7, 145.0, 144.8, 140.4, 139.3, 133.4, 132.6, 132.0, 131.1, 130.6, 130.2, 128.9, 128.4, 128.0, 127.3, 127.1, 125.6, 122.3, 120.4, 120.0, 119.6, 118.0, 117.9, 115.0, 114.9, 66.6, 39.9 ppm. IR (ATR): 3058, 2914, 1607, 1583, 1566, 1524, 1486, 1478, 1447, 1320, 1296, 1220, 1157, 1034, 740 cm-1. ESIMS (0V): *m/z* = 485 [$M+H^+$]. HRESIMS: $C_{28}H_{23}N_4Cl_2$ required 485.1300. Found: 485.1295.

***N*-(4-Bromophenyl)-2-((1-(4-bromophenyl)-1,2-dihydroquinazolin-4-yl)(methylimino)methyl)aniline** (**16d**): Yield: 385 mg (67%) of yellow crystals, mp: 209-210 °C. ^{1}H NMR (400 MHz, $CDCl_3$): δ = 11.71 (bs, 1H, NH-H), 7.55-7.51 (m, 2H, Ar-H), 7.45-7.41 (m, 2H, Ar-H), 7.33-7.29 (m, 2H, Ar-H), 7.26 (ddd, J = 8.2 / 6.9 / 1.3 Hz, 1H, Ar-H), 7.20-7.13 (m, 5H, Ar-H), 7.08 (dd, J = 7.8 / 1.3 Hz, 1H, Ar-H), 6.96 (d, J = 7.8 Hz, 1H, Ar-H), 6.80 (ddd, J = 7.6 / 7.6 / 1.0 Hz, 1H, Ar-H), 6.67 (ddd, J = 8.0 / 7.0 / 1.0 Hz, 1H, Ar-H), 5.46 (d, J = 1.3 Hz, 2H, CH_2), 3.38 (s, 3H, CH_3) ppm. ^{13}C NMR (100 MHz, $CDCl_3$): δ = 169.2, 164.5, 146.1, 144.5, 143.2, 140.9, 133.5, 132.8, 132.3, 132.1, 131.1, 127.3, 124.8, 123.5, 120.8, 119.5, 118.9, 117.5, 117.4, 116.3, 114.9, 114.1, 66.5, 40.1 ppm. IR (ATR): 2918, 2852, 1630, 1580, 1483, 1451, 1353, 1260, 1148, 1069, 943, 839, 715, 648, 543, 465 cm^{-1}. ESIMS: *m/z* (%) = 573 [$M+H^+$]. HRESIMS: $C_{28}H_{23}N_4Br_2$ required 573.0289. Found: 573.0288.

Crystal structure determinations of *N*-(1,1'-bis(3-chlorophenyl)-2-methyl-1,2-dihydrospiro[indazole,3,2'-indolin]-3'-ylidene)methanamine 14c

Crystal data for **14c**

$C_{28}H_{22}Cl_2N_4$	$F(000) = 1008$
$M_r = 485.39$	$D_x = 1.375$ Mg m^{-3}
Monoclinic, $P2_1/c$ (no.14)	Mo $K\alpha$ radiation, $\lambda = 0.71073$ Å
$a = 13.522$ (1) Å	Cell parameters from 1000 reflections
$b = 14.131$ (1) Å	$\theta = 2.5–25.0°$
$c = 12.306$ (1) Å	$\mu = 0.30$ mm^{-1}
$\beta = 94.16$ (1)°	$T = 123$ K
$V = 2345.2$ (3) Å^3	Blocks, yellow
$Z = 4$	0.45 × 0.25 × 0.15 mm

Data collection for **14c**

Bruker-Nonius KappaCCD diffractometer	4546 reflections with $I > 2\sigma(I)$
Radiation source: fine-focus sealed tube	$R_{int} = 0.028$
rotation in ϕ and ω, 2° scans	$\theta_{max} = 27.5°$, $\theta_{min} = 2.7°$
Absorption correction: multi-scan *SADABS* (Sheldrick, 2008)	$h = -17\rightarrow17$
$T_{min} = 0.806$, $T_{max} = 0.956$	$k = -18\rightarrow18$
38867 measured reflections	$l = -15\rightarrow15$
5367 independent reflections	

Refinement for **14c**

Refinement on F^2	Primary atom site location: structure-invariant direct methods
Least-squares matrix: full	Secondary atom site location: difference Fourier map
$R[F^2 > 2\sigma(F^2)] = 0.032$	Hydrogen site location: difference Fourier map
$wR(F^2) = 0.086$	H-atom parameters constrained
$S = 1.05$	$w = 1/[\sigma^2(F_o^2) + (0.0364P)^2 + 1.320P]$ where $P = (F_o^2 + 2F_c^2)/3$
5367 reflections	$(\Delta/\sigma)_{max} = 0.001$
309 parameters	$\Delta\rangle_{max} = 0.34$ e Å^{-3}
0 restraints	$\Delta\rangle_{min} = -0.29$ e Å^{-3}

Fractional atomic coordinates and isotropic or equivalent isotropic displacement parameters (Å^2) for **14c**

	x	*y*	*z*	U_{iso}*/U_{eq}
N1	0.66066 (8)	0.46525 (8)	0.73485 (9)	0.0185 (2)
N2	0.63131 (8)	0.46042 (8)	0.61818 (9)	0.0191 (2)
C3	0.71193 (10)	0.49760 (9)	0.56510 (11)	0.0203 (3)
C4	0.72575 (12)	0.49633 (10)	0.45401 (11)	0.0269 (3)
H4	0.6784	0.4682	0.4031	0.032*
C5	0.81154 (13)	0.53783 (10)	0.42065 (12)	0.0322 (4)
H5	0.8229	0.5377	0.3454	0.039*
C6	0.88125 (12)	0.57951 (11)	0.49421 (13)	0.0320 (3)
H6	0.9393	0.6073	0.4689	0.038*
C7	0.86620 (11)	0.58057 (10)	0.60488 (12)	0.0258 (3)
H7	0.9131	0.6093	0.6558	0.031*
C8	0.78113 (10)	0.53857 (9)	0.63884 (11)	0.0197 (3)
C9	0.74755 (9)	0.52908 (9)	0.75188 (10)	0.0177 (3)
N10	0.82183 (8)	0.48631 (8)	0.83171 (9)	0.0192 (2)
C11	0.82570 (9)	0.53719 (9)	0.92919 (11)	0.0193 (3)
C12	0.87972 (10)	0.51371 (10)	1.02610 (11)	0.0222 (3)
H12	0.9156	0.4560	1.0332	0.027*
C13	0.87938 (10)	0.57735 (11)	1.11180 (11)	0.0258 (3)
H13	0.9165	0.5631	1.1783	0.031*
C14	0.82613 (11)	0.66148 (11)	1.10308 (12)	0.0281 (3)
H14	0.8272	0.7036	1.1633	0.034*
C15	0.77137 (11)	0.68427 (10)	1.00676 (12)	0.0254 (3)
H15	0.7343	0.7414	1.0010	0.031*
C16	0.77157 (9)	0.62204 (9)	0.91834 (11)	0.0199 (3)
C17	0.72599 (9)	0.62540 (9)	0.80596 (10)	0.0185 (3)
N18	0.67796 (8)	0.68720 (8)	0.74859 (10)	0.0218 (2)
C19	0.65687 (12)	0.77887 (10)	0.79703 (13)	0.0280 (3)
H19A	0.6028	0.7718	0.8454	0.042*
H19B	0.6373	0.8242	0.7392	0.042*
H19C	0.7164	0.8020	0.8390	0.042*
C20	0.57368 (10)	0.48925 (11)	0.79293 (12)	0.0277 (3)
H20A	0.5454	0.5490	0.7646	0.042*
H20B	0.5931	0.4960	0.8708	0.042*
H20C	0.5241	0.4388	0.7823	0.042*
C21	0.59942 (10)	0.36651 (9)	0.58486 (11)	0.0205 (3)
C22	0.52904 (11)	0.35940 (10)	0.49696 (12)	0.0264 (3)
H22	0.5026	0.4149	0.4624	0.032*

C23	0.49766 (12)	0.27069 (11)	0.46009 (12)	0.0314 (3)
H23	0.4501	0.2658	0.3996	0.038*
C24	0.53489 (11)	0.18922 (11)	0.51058 (12)	0.0281 (3)
H24	0.5127	0.1286	0.4861	0.034*
C25	0.60525 (10)	0.19826 (10)	0.59780 (12)	0.0225 (3)
Cl25	0.65692 (3)	0.09567 (2)	0.65755 (3)	0.03012 (10)
C26	0.63787 (10)	0.28559 (9)	0.63677 (11)	0.0206 (3)
H26	0.6853	0.2902	0.6974	0.025*
C27	0.84207 (9)	0.38760 (9)	0.82831 (11)	0.0191 (3)
C28	0.80599 (10)	0.32595 (10)	0.90469 (12)	0.0240 (3)
H28	0.7669	0.3496	0.9597	0.029*
C29	0.82729 (11)	0.23009 (10)	0.90015 (12)	0.0274 (3)
H29	0.8029	0.1884	0.9526	0.033*
C30	0.88394 (11)	0.19428 (10)	0.81979 (12)	0.0259 (3)
H30	0.8981	0.1286	0.8163	0.031*
C31	0.91926 (10)	0.25654 (10)	0.74488 (11)	0.0216 (3)
Cl31	0.99549 (3)	0.21403 (3)	0.64726 (3)	0.02991 (10)
C32	0.89876 (9)	0.35274 (9)	0.74724 (11)	0.0196 (3)
H32	0.9230	0.3941	0.6944	0.023*

Atomic displacement parameters (Å^2) for **14c**

	U^{11}	U^{22}	U^{33}	U^{12}	U^{13}	U^{23}
N1	0.0202 (5)	0.0202 (5)	0.0152 (5)	-0.0032 (4)	0.0007 (4)	-0.0020 (4)
N2	0.0231 (5)	0.0180 (5)	0.0160 (5)	0.0007 (4)	-0.0009 (4)	-0.0011 (4)
C3	0.0258 (6)	0.0148 (6)	0.0204 (6)	0.0051 (5)	0.0028 (5)	0.0016 (5)
C4	0.0413 (8)	0.0199 (7)	0.0195 (6)	0.0062 (6)	0.0031 (6)	0.0010 (5)
C5	0.0516 (10)	0.0230 (7)	0.0242 (7)	0.0113 (7)	0.0168 (7)	0.0071 (6)
C6	0.0373 (8)	0.0227 (7)	0.0386 (8)	0.0044 (6)	0.0202 (7)	0.0080 (6)
C7	0.0268 (7)	0.0198 (7)	0.0317 (8)	0.0007 (5)	0.0083 (6)	0.0031 (6)
C8	0.0233 (6)	0.0150 (6)	0.0212 (6)	0.0029 (5)	0.0050 (5)	0.0013 (5)
C9	0.0170 (6)	0.0163 (6)	0.0199 (6)	-0.0005 (5)	0.0009 (5)	0.0006 (5)
N10	0.0199 (5)	0.0178 (5)	0.0196 (5)	0.0011 (4)	-0.0007 (4)	-0.0012 (4)
C11	0.0166 (6)	0.0210 (6)	0.0207 (6)	-0.0044 (5)	0.0035 (5)	-0.0017 (5)
C12	0.0183 (6)	0.0256 (7)	0.0226 (7)	-0.0019 (5)	0.0016 (5)	0.0009 (5)
C13	0.0220 (7)	0.0359 (8)	0.0195 (6)	-0.0063 (6)	0.0007 (5)	-0.0008 (6)
C14	0.0290 (7)	0.0331 (8)	0.0223 (7)	-0.0060 (6)	0.0036 (6)	-0.0086 (6)
C15	0.0247 (7)	0.0243 (7)	0.0277 (7)	-0.0009 (5)	0.0044 (6)	-0.0054 (6)
C16	0.0174 (6)	0.0208 (6)	0.0217 (6)	-0.0032 (5)	0.0027 (5)	-0.0014 (5)

C17	0.0171 (6)	0.0180 (6)	0.0208 (6)	-0.0032 (5)	0.0037 (5)	-0.0032 (5)
N18	0.0219 (5)	0.0182 (5)	0.0253 (6)	0.0005 (4)	0.0020 (4)	-0.0016 (4)
C19	0.0324 (8)	0.0196 (7)	0.0319 (8)	0.0037 (6)	0.0014 (6)	-0.0037 (6)
C20	0.0235 (7)	0.0332 (8)	0.0273 (7)	-0.0086 (6)	0.0085 (6)	-0.0080 (6)
C21	0.0214 (6)	0.0194 (6)	0.0207 (6)	0.0002 (5)	0.0014 (5)	-0.0039 (5)
C22	0.0299 (7)	0.0248 (7)	0.0235 (7)	0.0031 (6)	-0.0045 (6)	-0.0018 (6)
C23	0.0358 (8)	0.0318 (8)	0.0252 (7)	-0.0012 (6)	-0.0078 (6)	-0.0079 (6)
C24	0.0317 (7)	0.0241 (7)	0.0284 (7)	-0.0032 (6)	0.0018 (6)	-0.0091 (6)
C25	0.0216 (6)	0.0193 (6)	0.0272 (7)	0.0023 (5)	0.0054 (5)	-0.0003 (5)
Cl25	0.02898 (18)	0.01933 (17)	0.0422 (2)	0.00167 (13)	0.00402 (15)	0.00354 (14)
C26	0.0183 (6)	0.0214 (6)	0.0221 (6)	0.0002 (5)	0.0012 (5)	-0.0011 (5)
C27	0.0162 (6)	0.0178 (6)	0.0228 (6)	-0.0003 (5)	-0.0019 (5)	-0.0002 (5)
C28	0.0219 (6)	0.0243 (7)	0.0262 (7)	-0.0018 (5)	0.0044 (5)	0.0004 (5)
C29	0.0274 (7)	0.0228 (7)	0.0323 (8)	-0.0044 (6)	0.0043 (6)	0.0060 (6)
C30	0.0240 (7)	0.0176 (6)	0.0357 (8)	-0.0001 (5)	-0.0004 (6)	0.0009 (6)
C31	0.0173 (6)	0.0229 (7)	0.0242 (7)	0.0013 (5)	-0.0007 (5)	-0.0039 (5)
Cl31	0.03333 (19)	0.02462 (18)	0.03258 (19)	0.00530 (14)	0.00790 (14)	-0.00552 (14)
C32	0.0181 (6)	0.0198 (6)	0.0204 (6)	-0.0015 (5)	-0.0007 (5)	0.0002 (5)

Geometric parameters (Å, º) for **14c**

N1—C20	1.4598 (17)	C17—N18	1.2713 (17)
N1—N2	1.4632 (14)	N18—C19	1.4626 (18)
N1—C9	1.4840 (16)	C19—H19A	0.9800
N2—C3	1.4122 (18)	C19—H19B	0.9800
N2—C21	1.4455 (17)	C19—H19C	0.9800
C3—C8	1.3824 (19)	C20—H20A	0.9800
C3—C4	1.3932 (19)	C20—H20B	0.9800
C4—C5	1.388 (2)	C20—H20C	0.9800
C4—H4	0.9500	C21—C22	1.3915 (18)
C5—C6	1.390 (2)	C21—C26	1.3920 (18)
C5—H5	0.9500	C22—C23	1.389 (2)
C6—C7	1.392 (2)	C22—H22	0.9500
C6—H6	0.9500	C23—C24	1.385 (2)
C7—C8	1.3855 (19)	C23—H23	0.9500
C7—H7	0.9500	C24—C25	1.388 (2)
C8—C9	1.5004 (18)	C24—H24	0.9500
C9—N10	1.4819 (16)	C25—C26	1.3845 (19)

C9—C17	1.5520 (18)	C25—Cl25	1.7482 (14)
N10—C11	1.3963 (17)	C26—H26	0.9500
N10—C27	1.4227 (17)	C27—C32	1.3916 (19)
C11—C12	1.3927 (18)	C27—C28	1.3954 (19)
C11—C16	1.4061 (19)	C28—C29	1.387 (2)
C12—C13	1.386 (2)	C28—H28	0.9500
C12—H12	0.9500	C29—C30	1.390 (2)
C13—C14	1.390 (2)	C29—H29	0.9500
C13—H13	0.9500	C30—C31	1.384 (2)
C14—C15	1.389 (2)	C30—H30	0.9500
C14—H14	0.9500	C31—C32	1.3881 (19)
C15—C16	1.3992 (19)	C31—Cl31	1.7453 (14)
C15—H15	0.9500	C32—H32	0.9500
C16—C17	1.4734 (18)		
C20—N1—N2	108.69 (10)	N18—C17—C9	117.95 (11)
C20—N1—C9	116.58 (10)	C16—C17—C9	107.18 (11)
N2—N1—C9	108.82 (10)	C17—N18—C19	119.27 (12)
C3—N2—C21	115.77 (11)	N18—C19—H19A	109.5
C3—N2—N1	105.84 (10)	N18—C19—H19B	109.5
C21—N2—N1	112.04 (10)	H19A—C19—H19B	109.5
C8—C3—C4	121.12 (13)	N18—C19—H19C	109.5
C8—C3—N2	111.14 (11)	H19A—C19—H19C	109.5
C4—C3—N2	127.73 (13)	H19B—C19—H19C	109.5
C5—C4—C3	117.33 (14)	N1—C20—H20A	109.5
C5—C4—H4	121.3	N1—C20—H20B	109.5
C3—C4—H4	121.3	H20A—C20—H20B	109.5
C4—C5—C6	121.89 (14)	N1—C20—H20C	109.5
C4—C5—H5	119.1	H20A—C20—H20C	109.5
C6—C5—H5	119.1	H20B—C20—H20C	109.5
C5—C6—C7	120.11 (14)	C22—C21—C26	120.60 (12)
C5—C6—H6	119.9	C22—C21—N2	117.38 (12)
C7—C6—H6	119.9	C26—C21—N2	122.02 (11)
C8—C7—C6	118.30 (14)	C23—C22—C21	119.64 (13)
C8—C7—H7	120.8	C23—C22—H22	120.2
C6—C7—H7	120.8	C21—C22—H22	120.2
C3—C8—C7	121.24 (13)	C24—C23—C22	120.73 (13)
C3—C8—C9	109.58 (11)	C24—C23—H23	119.6
C7—C8—C9	129.17 (13)	C22—C23—H23	119.6
N10—C9—N1	109.72 (10)	C23—C24—C25	118.48 (13)
N10—C9—C8	114.51 (11)	C23—C24—H24	120.8

N1—C9—C8	102.31 (10)	C25—C24—H24	120.8
N10—C9—C17	102.29 (10)	C26—C25—C24	122.24 (13)
N1—C9—C17	114.97 (10)	C26—C25—Cl25	119.07 (11)
C8—C9—C17	113.45 (10)	C24—C25—Cl25	118.66 (11)
C11—N10—C27	122.33 (11)	C25—C26—C21	118.31 (12)
C11—N10—C9	109.93 (10)	C25—C26—H26	120.8
C27—N10—C9	120.20 (10)	C21—C26—H26	120.8
C12—C11—N10	126.70 (12)	C32—C27—C28	119.98 (12)
C12—C11—C16	121.44 (12)	C32—C27—N10	119.03 (12)
N10—C11—C16	111.76 (11)	C28—C27—N10	121.00 (12)
C13—C12—C11	117.80 (13)	C29—C28—C27	119.93 (13)
C13—C12—H12	121.1	C29—C28—H28	120.0
C11—C12—H12	121.1	C27—C28—H28	120.0
C12—C13—C14	121.76 (13)	C28—C29—C30	120.74 (13)
C12—C13—H13	119.1	C28—C29—H29	119.6
C14—C13—H13	119.1	C30—C29—H29	119.6
C15—C14—C13	120.34 (13)	C31—C30—C29	118.50 (13)
C15—C14—H14	119.8	C31—C30—H30	120.7
C13—C14—H14	119.8	C29—C30—H30	120.7
C14—C15—C16	119.14 (14)	C30—C31—C32	121.97 (13)
C14—C15—H15	120.4	C30—C31—Cl31	119.29 (11)
C16—C15—H15	120.4	C32—C31—Cl31	118.70 (11)
C15—C16—C11	119.50 (12)	C31—C32—C27	118.87 (12)
C15—C16—C17	133.27 (13)	C31—C32—H32	120.6
C11—C16—C17	107.22 (11)	C27—C32—H32	120.6
N18—C17—C16	134.86 (12)		
C20—N1—N2—C3	143.11 (11)	C14—C15—C16—C11	1.0 (2)
C9—N1—N2—C3	15.22 (13)	C14—C15—C16—C17	-177.51 (14)
C20—N1—N2—C21	-89.84 (13)	C12—C11—C16—C15	-0.3 (2)
C9—N1—N2—C21	142.26 (11)	N10—C11—C16—C15	-176.88 (12)
C21—N2—C3—C8	-134.40 (12)	C12—C11—C16—C17	178.59 (12)
N1—N2—C3—C8	-9.64 (14)	N10—C11—C16—C17	1.99 (15)
C21—N2—C3—C4	45.66 (18)	C15—C16—C17—N18	6.3 (3)
N1—N2—C3—C4	170.42 (13)	C11—C16—C17—N18	-172.39 (15)
C8—C3—C4—C5	-0.1 (2)	C15—C16—C17—C9	-175.18 (14)
N2—C3—C4—C5	179.88 (13)	C11—C16—C17—C9	6.17 (14)
C3—C4—C5—C6	-0.2 (2)	N10—C9—C17—N18	167.56 (11)
C4—C5—C6—C7	0.0 (2)	N1—C9—C17—N18	-73.60 (15)
C5—C6—C7—C8	0.6 (2)	C8—C9—C17—N18	43.68 (16)
C4—C3—C8—C7	0.6 (2)	N10—C9—C17—C16	-11.29 (13)

N2—C3—C8—C7	-179.35 (12)	N1—C9—C17—C16	107.56 (12)
C4—C3—C8—C9	-179.60 (12)	C8—C9—C17—C16	-135.16 (11)
N2—C3—C8—C9	0.46 (15)	C16—C17—N18—C19	-1.3 (2)
C6—C7—C8—C3	-0.8 (2)	C9—C17—N18—C19	-179.71 (12)
C6—C7—C8—C9	179.40 (13)	C3—N2—C21—C22	-88.19 (15)
C20—N1—C9—N10	100.31 (13)	N1—N2—C21—C22	150.32 (12)
N2—N1—C9—N10	-136.39 (10)	C3—N2—C21—C26	90.92 (15)
C20—N1—C9—C8	-137.73 (12)	N1—N2—C21—C26	-30.58 (17)
N2—N1—C9—C8	-14.43 (12)	C26—C21—C22—C23	-0.6 (2)
C20—N1—C9—C17	-14.29 (16)	N2—C21—C22—C23	178.48 (13)
N2—N1—C9—C17	109.01 (12)	C21—C22—C23—C24	0.7 (2)
C3—C8—C9—N10	127.28 (12)	C22—C23—C24—C25	-0.9 (2)
C7—C8—C9—N10	-52.93 (18)	C23—C24—C25—C26	1.1 (2)
C3—C8—C9—N1	8.66 (13)	C23—C24—C25—Cl25	-176.91 (12)
C7—C8—C9—N1	-171.56 (13)	C24—C25—C26—C21	-1.1 (2)
C3—C8—C9—C17	-115.79 (12)	Cl25—C25—C26—C21	176.95 (10)
C7—C8—C9—C17	63.99 (18)	C22—C21—C26—C25	0.8 (2)
N1—C9—N10—C11	-109.76 (12)	N2—C21—C26—C25	-178.25 (12)
C8—C9—N10—C11	135.89 (11)	C11—N10—C27—C32	-138.16 (13)
C17—C9—N10—C11	12.72 (13)	C9—N10—C27—C32	75.22 (16)
N1—C9—N10—C27	40.60 (15)	C11—N10—C27—C28	41.85 (18)
C8—C9—N10—C27	-73.75 (15)	C9—N10—C27—C28	-104.77 (15)
C17—C9—N10—C27	163.08 (11)	C32—C27—C28—C29	0.5 (2)
C27—N10—C11—C12	24.2 (2)	N10—C27—C28—C29	-179.56 (12)
C9—N10—C11—C12	173.77 (12)	C27—C28—C29—C30	-0.3 (2)
C27—N10—C11—C16	-159.47 (12)	C28—C29—C30—C31	0.5 (2)
C9—N10—C11—C16	-9.86 (15)	C29—C30—C31—C32	-0.7 (2)
N10—C11—C12—C13	175.39 (13)	C29—C30—C31—Cl31	176.95 (11)
C16—C11—C12—C13	-0.7 (2)	C30—C31—C32—C27	0.9 (2)
C11—C12—C13—C14	0.9 (2)	Cl31—C31—C32—C27	-176.85 (10)
C12—C13—C14—C15	-0.2 (2)	C28—C27—C32—C31	-0.70 (19)
C13—C14—C15—C16	-0.8 (2)	N10—C27—C32—C31	179.31 (11)

Hydrogen-bond geometry (Å, º) for **14c**

D—H···*A*	*D*—H	H···*A*	*D*···*A*	*D*—H···*A*
C12—H12···Cl31^{i}	0.95	2.95	3.8330 (15)	156
C20—H20*A*···N18	0.98	2.67	3.1973 (19)	114
C22—H22···N2^{ii}	0.95	2.67	3.5702 (18)	159

Symmetry codes: (i) *x*, -*y*+1/2, *z*+1/2; (ii) -*x*+1, -*y*+1, -*z*+1.

Crystal structure determination of carbonylbis(triphenylphosphine)(2-methyl-1-phenyl-1*H*-indazole-3-ylidene)rhodium(I) hexafluorophosphate (15e)

Crystal data for **15e**

$C_{51}H_{41}IN_2OP_2Rh\cdot F_6P\cdot 3(CH_2Cl_2)$	F(000) = 2768
M_r = 1389.35	D_x = 1.627 Mg m^{-3}
Monoclinic, $P2_1/n$ *(no.14)*	Mo $K\alpha$ radiation, λ = 0.71073 Å
a = 14.314 (1) Å	Cell parameters from 767 reflections
b = 26.691 (2) Å	θ = 2.5–25.0°
c = 15.045 (1) Å	μ = 1.27 mm^{-1}
β = 99.43 (1)°	T = 123 K
V = 5670.3 (7) Å^3	Blocks, yellow
Z = 4	0.35 × 0.25 × 0.15 mm

Data collection for **15e**

Bruker-Nonius KappaCCD diffractometer	11838 reflections with $I > 2\sigma(I)$
Radiation source: fine-focus sealed tube	R_{int} = 0.021
rotation in ϕ and ω, 1° scans	θ_{max} = 27.5°, θ_{min} = 2.6°
Absorption correction: multi-scan *SADABS* (Sheldrick, 2008)	h = -18→18
T_{min} = 0.727, T_{max} = 0.813	k = -34→34
91339 measured reflections	l = -19→19
12976 independent reflections	

Refinement for **15e**

Refinement on F^2	Primary atom site location: structure-invariant direct methods
Least-squares matrix: full	Secondary atom site location: difference Fourier map
$R[F^2 > 2\sigma(F^2)]$ = 0.032	Hydrogen site location: mixed
$wR(F^2)$ = 0.086	H-atom parameters constrained
S = 1.09	$w = 1/[\sigma^2(F_o^2) + (0.0367P)^2 + 12.4324P]$ where $P = (F_o^2 + 2F_c^2)/3$
12976 reflections	$(\Delta/\sigma)_{max}$ = 0.001
685 parameters	$\Delta\rangle_{max}$ = 1.47 e Å^{-3}
70 restraints	$\Delta\rangle_{min}$ = -1.38 e Å^{-3}

Special details for **15e**

Refinement: CH_2Cl_2 disordered, use of geoemtical restraints (SADI) and restraints for the displacement parameters (SIMU). Use of rigid bond refinement (RIGU).

Fractional atomic coordinates and isotropic or equivalent isotropic displacement parameters (Å^2) for **15e**

	x	*y*	*z*	U_{iso}*/U_{eq}	Occ. (<1)
Rh1	0.17605 (2)	0.39997 (2)	0.54858 (2)	0.01213 (5)	
C1	0.31186 (17)	0.38441 (9)	0.52799 (16)	0.0146 (4)	
N2	0.36383 (14)	0.34414 (8)	0.55417 (14)	0.0155 (4)	
N3	0.45326 (15)	0.34600 (8)	0.52874 (15)	0.0194 (4)	
C4	0.45614 (18)	0.38911 (10)	0.48070 (18)	0.0195 (5)	
C5	0.5278 (2)	0.40793 (12)	0.4369 (2)	0.0298 (6)	
H5	0.5855	0.3904	0.4369	0.036*	
C6	0.5100 (2)	0.45332 (13)	0.3938 (3)	0.0395 (8)	
H6	0.5570	0.4673	0.3633	0.047*	
C7	0.4248 (2)	0.47958 (12)	0.3934 (3)	0.0385 (8)	
H7	0.4157	0.5108	0.3630	0.046*	
C8	0.3545 (2)	0.46091 (11)	0.4361 (2)	0.0265 (6)	
H8	0.2970	0.4788	0.4358	0.032*	
C9	0.37010 (17)	0.41455 (10)	0.48006 (17)	0.0176 (5)	
C10	0.05502 (18)	0.42217 (10)	0.55928 (17)	0.0195 (5)	
O10	-0.01850 (14)	0.43816 (8)	0.56286 (15)	0.0303 (5)	
P1	0.23524 (4)	0.42962 (2)	0.69127 (4)	0.01327 (12)	
C11	0.15576 (17)	0.41683 (10)	0.77154 (17)	0.0174 (5)	
C12	0.10366 (18)	0.37259 (11)	0.76096 (19)	0.0225 (5)	
H12	0.1079	0.3510	0.7116	0.027*	
C13	0.0453 (2)	0.36016 (12)	0.8231 (2)	0.0306 (7)	
H13	0.0100	0.3299	0.8164	0.037*	
C14	0.0386 (2)	0.39169 (14)	0.8947 (2)	0.0352 (7)	
H14	-0.0016	0.3832	0.9368	0.042*	
C15	0.0901 (2)	0.43549 (13)	0.9050 (2)	0.0315 (7)	
H15	0.0855	0.4570	0.9544	0.038*	
C16	0.1488 (2)	0.44846 (11)	0.84357 (18)	0.0238 (5)	
H16	0.1840	0.4788	0.8508	0.029*	
C17	0.25500 (17)	0.49694 (9)	0.69613 (17)	0.0164 (5)	
C18	0.31519 (19)	0.51972 (10)	0.76716 (18)	0.0209 (5)	
H18	0.3480	0.4998	0.8146	0.025*	
C19	0.3272 (2)	0.57137 (10)	0.7686 (2)	0.0249 (6)	
H19	0.3686	0.5866	0.8169	0.030*	

C20	0.2792 (2)	0.60075 (10)	0.7003 (2)	0.0264 (6)	
H20	0.2880	0.6360	0.7016	0.032*	
C21	0.2184 (2)	0.57881 (10)	0.6301 (2)	0.0259 (6)	
H21	0.1846	0.5991	0.5838	0.031*	
C22	0.20681 (19)	0.52697 (10)	0.62744 (18)	0.0208 (5)	
H22	0.1659	0.5120	0.5786	0.025*	
C23	0.34784 (17)	0.40443 (9)	0.74924 (17)	0.0167 (5)	
C24	0.43167 (18)	0.41852 (10)	0.71970 (18)	0.0201 (5)	
H24	0.4294	0.4409	0.6703	0.024*	
C25	0.5182 (2)	0.40002 (11)	0.7621 (2)	0.0272 (6)	
H25	0.5749	0.4101	0.7420	0.033*	
C26	0.5221 (2)	0.36694 (12)	0.8336 (2)	0.0325 (7)	
H26	0.5815	0.3546	0.8629	0.039*	
C27	0.4396 (2)	0.35195 (12)	0.8623 (2)	0.0320 (7)	
H27	0.4423	0.3288	0.9106	0.038*	
C28	0.3522 (2)	0.37070 (11)	0.82056 (19)	0.0256 (6)	
H28	0.2956	0.3605	0.8408	0.031*	
P2	0.12011 (4)	0.36404 (2)	0.40994 (4)	0.01325 (12)	
C29	0.00598 (17)	0.33223 (9)	0.40004 (17)	0.0161 (5)	
C30	-0.04321 (19)	0.31842 (10)	0.31547 (18)	0.0220 (5)	
H30	-0.0188	0.3272	0.2625	0.026*	
C31	-0.1275 (2)	0.29196 (11)	0.3086 (2)	0.0261 (6)	
H31	-0.1608	0.2827	0.2511	0.031*	
C32	-0.16322 (19)	0.27900 (11)	0.3855 (2)	0.0271 (6)	
H32	-0.2212	0.2611	0.3806	0.032*	
C33	-0.1147 (2)	0.29201 (11)	0.4695 (2)	0.0259 (6)	
H33	-0.1390	0.2827	0.5222	0.031*	
C34	-0.03037 (18)	0.31870 (10)	0.47677 (18)	0.0194 (5)	
H34	0.0026	0.3277	0.5346	0.023*	
C35	0.10539 (17)	0.40849 (9)	0.31668 (17)	0.0172 (5)	
C36	0.10969 (19)	0.39377 (10)	0.22821 (18)	0.0207 (5)	
H36	0.1208	0.3597	0.2153	0.025*	
C37	0.0977 (2)	0.42899 (11)	0.15922 (19)	0.0267 (6)	
H37	0.1011	0.4189	0.0993	0.032*	
C38	0.0810 (2)	0.47860 (12)	0.1775 (2)	0.0315 (7)	
H38	0.0736	0.5026	0.1303	0.038*	
C39	0.0750 (2)	0.49330 (11)	0.2646 (2)	0.0324 (7)	
H39	0.0618	0.5273	0.2769	0.039*	
C40	0.0882 (2)	0.45861 (10)	0.33400 (19)	0.0230 (5)	
H40	0.0855	0.4691	0.3939	0.028*	
C41	0.19622 (17)	0.31474 (9)	0.37700 (17)	0.0164 (5)	

C42	0.27195 (18)	0.32606 (10)	0.33250 (18)	0.0204 (5)	
H42	0.2818	0.3596	0.3149	0.025*	
C43	0.33296 (19)	0.28829 (12)	0.31400 (19)	0.0270 (6)	
H43	0.3833	0.2958	0.2821	0.032*	
C44	0.3201 (2)	0.23966 (12)	0.3422 (2)	0.0311 (7)	
H44	0.3624	0.2140	0.3305	0.037*	
C45	0.2460 (2)	0.22843 (11)	0.3871 (2)	0.0291 (6)	
H45	0.2378	0.1951	0.4065	0.035*	
C46	0.18329 (18)	0.26559 (10)	0.40425 (19)	0.0217 (5)	
H46	0.1319	0.2576	0.4344	0.026*	
C47	0.33686 (19)	0.30124 (10)	0.60460 (19)	0.0213 (5)	
H47A	0.3738	0.3015	0.6655	0.032*	
H47B	0.3495	0.2702	0.5739	0.032*	
H47C	0.2692	0.3033	0.6082	0.032*	
C48	0.51952 (17)	0.30546 (10)	0.53832 (18)	0.0180 (5)	
C49	0.56074 (18)	0.28883 (10)	0.62328 (18)	0.0209 (5)	
H49	0.5434	0.3035	0.6757	0.025*	
C50	0.62763 (19)	0.25055 (10)	0.63109 (18)	0.0212 (5)	
H50	0.6556	0.2386	0.6888	0.025*	
C51	0.65317 (17)	0.22994 (9)	0.55332 (18)	0.0185 (5)	
I51	0.75570 (2)	0.17337 (2)	0.56257 (2)	0.02400 (5)	
C52	0.61226 (18)	0.24690 (10)	0.46895 (18)	0.0201 (5)	
H52	0.6300	0.2326	0.4163	0.024*	
C53	0.54518 (17)	0.28497 (10)	0.46149 (18)	0.0203 (5)	
H53	0.5171	0.2969	0.4038	0.024*	
P3	0.07747 (5)	0.22075 (3)	0.66487 (5)	0.02170 (14)	
F1	-0.00972 (13)	0.25546 (7)	0.67967 (14)	0.0366 (4)	
F2	0.16575 (13)	0.18652 (8)	0.65092 (14)	0.0385 (4)	
F3	0.12432 (13)	0.26752 (7)	0.62351 (15)	0.0415 (5)	
F4	0.03068 (18)	0.17413 (9)	0.7071 (2)	0.0639 (8)	
F5	0.02453 (16)	0.20630 (9)	0.56773 (15)	0.0518 (6)	
F6	0.13114 (18)	0.23533 (11)	0.76215 (14)	0.0630 (7)	
C1C	0.3481 (3)	0.11817 (13)	0.6090 (2)	0.0406 (8)	
H1C1	0.3872	0.1016	0.6611	0.049*	
H1C2	0.2917	0.1325	0.6299	0.049*	
Cl1	0.41389 (6)	0.16681 (3)	0.57047 (5)	0.03550 (17)	
Cl2	0.31153 (11)	0.07353 (4)	0.52540 (7)	0.0676 (3)	
C2C	0.3039 (3)	0.13076 (16)	0.1850 (2)	0.0492 (9)	
H2C1	0.2878	0.1669	0.1822	0.059*	
H2C2	0.3712	0.1274	0.1781	0.059*	
Cl3	0.28870 (6)	0.10686 (3)	0.29028 (6)	0.04066 (18)	

Cl4	0.23329 (9)	0.09966 (5)	0.09666 (8)	0.0673 (3)	
C3C	0.4220 (6)	0.4553 (4)	1.1367 (5)	0.109 (3)*	0.742 (3)
H3C1	0.4724	0.4803	1.1554	0.130*	0.742 (3)
H3C2	0.4328	0.4268	1.1792	0.130*	0.742 (3)
Cl5	0.3148 (2)	0.48155 (16)	1.1429 (3)	0.1510 (12)	0.742 (3)
Cl6	0.4299 (3)	0.43462 (18)	1.0312 (3)	0.1715 (14)	0.742 (3)
C3C'	0.4226 (12)	0.4601 (8)	1.103 (2)	0.109 (3)*	0.258 (3)
H3C3	0.4428	0.4855	1.1500	0.130*	0.258 (3)
H3C4	0.4732	0.4564	1.0658	0.130*	0.258 (3)
Cl5'	0.4017 (8)	0.4031 (5)	1.1522 (9)	0.1730 (19)	0.258 (3)
Cl6'	0.3181 (8)	0.4783 (5)	1.0363 (9)	0.1794 (19)	0.258 (3)

Atomic displacement parameters (Å^2) for **15e**

	U^{11}	U^{22}	U^{33}	U^{12}	U^{13}	U^{23}
Rh1	0.01143 (9)	0.01316 (9)	0.01219 (9)	0.00070 (6)	0.00308 (6)	-0.00061 (6)
C1	0.0153 (11)	0.0158 (11)	0.0131 (11)	-0.0008 (9)	0.0032 (9)	-0.0017 (9)
N2	0.0123 (9)	0.0178 (10)	0.0173 (10)	0.0003 (8)	0.0053 (8)	0.0012 (8)
N3	0.0140 (10)	0.0211 (11)	0.0247 (11)	0.0021 (8)	0.0083 (8)	0.0046 (9)
C4	0.0176 (12)	0.0199 (12)	0.0216 (13)	-0.0004 (9)	0.0051 (10)	0.0029 (10)
C5	0.0219 (13)	0.0319 (15)	0.0392 (17)	0.0016 (11)	0.0156 (12)	0.0075 (13)
C6	0.0339 (17)	0.0360 (17)	0.056 (2)	0.0002 (14)	0.0277 (16)	0.0168 (16)
C7	0.0390 (17)	0.0288 (16)	0.053 (2)	0.0055 (13)	0.0227 (16)	0.0211 (15)
C8	0.0243 (13)	0.0241 (14)	0.0335 (15)	0.0034 (11)	0.0123 (12)	0.0084 (12)
C9	0.0161 (11)	0.0205 (12)	0.0169 (12)	-0.0004 (9)	0.0043 (9)	0.0011 (9)
C10	0.0216 (12)	0.0223 (12)	0.0143 (11)	-0.0008 (10)	0.0023 (9)	-0.0039 (9)
O10	0.0184 (9)	0.0387 (12)	0.0339 (11)	0.0092 (8)	0.0050 (8)	-0.0105 (9)
P1	0.0139 (3)	0.0135 (3)	0.0129 (3)	-0.0007 (2)	0.0035 (2)	0.0004 (2)
C11	0.0160 (11)	0.0214 (12)	0.0150 (11)	0.0017 (9)	0.0036 (9)	0.0040 (9)
C12	0.0194 (12)	0.0261 (13)	0.0221 (13)	-0.0029 (10)	0.0040 (10)	0.0042 (10)
C13	0.0217 (13)	0.0384 (17)	0.0319 (16)	-0.0055 (12)	0.0053 (11)	0.0161 (13)
C14	0.0256 (14)	0.056 (2)	0.0267 (15)	0.0057 (14)	0.0137 (12)	0.0195 (14)
C15	0.0349 (16)	0.0439 (18)	0.0184 (13)	0.0107 (13)	0.0127 (12)	0.0062 (12)
C16	0.0277 (13)	0.0275 (14)	0.0174 (12)	0.0034 (11)	0.0074 (10)	0.0026 (10)
C17	0.0176 (11)	0.0138 (11)	0.0191 (12)	-0.0013 (9)	0.0066 (9)	0.0001 (9)
C18	0.0211 (12)	0.0198 (12)	0.0216 (13)	-0.0015 (10)	0.0029 (10)	-0.0021 (10)
C19	0.0251 (13)	0.0193 (13)	0.0305 (15)	-0.0037 (10)	0.0054 (11)	-0.0073 (11)
C20	0.0301 (14)	0.0143 (12)	0.0369 (16)	-0.0010 (10)	0.0116 (12)	-0.0027 (11)
C21	0.0310 (14)	0.0188 (13)	0.0290 (15)	0.0046 (11)	0.0081 (12)	0.0048 (11)

C22	0.0234 (12)	0.0182 (12)	0.0207 (13)	0.0000 (10)	0.0030 (10)	0.0012 (10)
C23	0.0176 (11)	0.0158 (11)	0.0160 (11)	0.0010 (9)	0.0010 (9)	-0.0017 (9)
C24	0.0190 (12)	0.0206 (12)	0.0206 (12)	0.0000 (10)	0.0024 (10)	0.0000 (10)
C25	0.0187 (13)	0.0298 (15)	0.0325 (15)	0.0012 (11)	0.0026 (11)	-0.0035 (12)
C26	0.0241 (14)	0.0356 (16)	0.0345 (16)	0.0089 (12)	-0.0047 (12)	0.0003 (13)
C27	0.0352 (16)	0.0331 (16)	0.0252 (15)	0.0087 (13)	-0.0023 (12)	0.0093 (12)
C28	0.0242 (13)	0.0274 (14)	0.0247 (14)	0.0028 (11)	0.0027 (11)	0.0067 (11)
P2	0.0132 (3)	0.0138 (3)	0.0129 (3)	0.0006 (2)	0.0028 (2)	-0.0009 (2)
C29	0.0140 (11)	0.0143 (11)	0.0199 (12)	0.0013 (8)	0.0025 (9)	0.0006 (9)
C30	0.0210 (12)	0.0256 (13)	0.0193 (12)	-0.0022 (10)	0.0033 (10)	-0.0021 (10)
C31	0.0212 (13)	0.0254 (14)	0.0295 (15)	-0.0030 (11)	-0.0026 (11)	-0.0038 (11)
C32	0.0171 (12)	0.0239 (13)	0.0389 (16)	-0.0046 (10)	0.0007 (11)	0.0030 (12)
C33	0.0234 (13)	0.0249 (14)	0.0304 (15)	-0.0023 (11)	0.0072 (11)	0.0069 (11)
C34	0.0194 (12)	0.0184 (12)	0.0196 (12)	-0.0001 (9)	0.0012 (10)	0.0043 (10)
C35	0.0163 (11)	0.0187 (12)	0.0168 (12)	0.0002 (9)	0.0037 (9)	0.0012 (9)
C36	0.0237 (13)	0.0207 (12)	0.0179 (12)	0.0014 (10)	0.0038 (10)	-0.0004 (10)
C37	0.0338 (15)	0.0294 (15)	0.0178 (13)	0.0035 (12)	0.0070 (11)	0.0036 (11)
C38	0.0438 (17)	0.0280 (15)	0.0241 (14)	0.0087 (13)	0.0101 (13)	0.0112 (12)
C39	0.0484 (18)	0.0207 (13)	0.0301 (16)	0.0091 (13)	0.0123 (14)	0.0050 (12)
C40	0.0295 (14)	0.0207 (13)	0.0201 (13)	0.0042 (10)	0.0074 (11)	-0.0004 (10)
C41	0.0149 (11)	0.0187 (11)	0.0153 (11)	0.0019 (9)	0.0013 (9)	-0.0049 (9)
C42	0.0162 (11)	0.0272 (13)	0.0176 (12)	0.0004 (10)	0.0018 (9)	-0.0063 (10)
C43	0.0145 (12)	0.0406 (16)	0.0249 (14)	0.0030 (11)	0.0001 (10)	-0.0129 (12)
C44	0.0197 (13)	0.0313 (15)	0.0395 (17)	0.0102 (11)	-0.0033 (12)	-0.0157 (13)
C45	0.0252 (14)	0.0203 (13)	0.0389 (17)	0.0055 (11)	-0.0032 (12)	-0.0064 (12)
C46	0.0187 (12)	0.0189 (12)	0.0266 (14)	0.0025 (10)	0.0011 (10)	-0.0026 (10)
C47	0.0214 (12)	0.0164 (12)	0.0280 (14)	0.0012 (9)	0.0094 (10)	0.0064 (10)
C48	0.0125 (10)	0.0195 (12)	0.0227 (13)	0.0019 (9)	0.0047 (9)	0.0012 (10)
C49	0.0201 (12)	0.0251 (13)	0.0181 (12)	0.0025 (10)	0.0053 (10)	-0.0009 (10)
C50	0.0213 (12)	0.0230 (13)	0.0188 (12)	0.0019 (10)	0.0017 (10)	0.0029 (10)
C51	0.0129 (11)	0.0177 (11)	0.0254 (13)	0.0006 (9)	0.0045 (9)	0.0011 (10)
I51	0.02094 (9)	0.02196 (9)	0.03022 (10)	0.00576 (6)	0.00747 (7)	0.00444 (7)
C52	0.0168 (11)	0.0244 (13)	0.0198 (12)	0.0004 (10)	0.0049 (9)	-0.0027 (10)
C53	0.0153 (11)	0.0271 (13)	0.0181 (12)	0.0005 (10)	0.0015 (9)	0.0008 (10)
P3	0.0202 (3)	0.0240 (3)	0.0213 (3)	0.0017 (3)	0.0043 (3)	0.0051 (3)
F1	0.0279 (9)	0.0362 (10)	0.0485 (11)	0.0097 (8)	0.0147 (8)	0.0072 (9)
F2	0.0314 (9)	0.0417 (11)	0.0442 (11)	0.0161 (8)	0.0115 (8)	0.0069 (9)
F3	0.0314 (10)	0.0353 (10)	0.0613 (13)	-0.0035 (8)	0.0177 (9)	0.0143 (9)
F4	0.0553 (14)	0.0414 (13)	0.105 (2)	0.0088 (10)	0.0423 (14)	0.0380 (13)
F5	0.0472 (12)	0.0544 (13)	0.0459 (12)	0.0062 (10)	-0.0163 (10)	-0.0175 (10)
F6	0.0619 (15)	0.095 (2)	0.0264 (11)	0.0256 (14)	-0.0104 (10)	-0.0120 (12)

C1C	0.047 (2)	0.046 (2)	0.0323 (17)	-0.0003 (16)	0.0159 (15)	-0.0008 (15)
Cl1	0.0455 (4)	0.0323 (4)	0.0306 (4)	0.0084 (3)	0.0118 (3)	0.0036 (3)
Cl2	0.1118 (10)	0.0503 (6)	0.0415 (5)	-0.0253 (6)	0.0154 (6)	-0.0041 (4)
C2C	0.049 (2)	0.055 (2)	0.041 (2)	-0.0100 (18)	0.0001 (17)	-0.0057 (18)
Cl3	0.0384 (4)	0.0412 (4)	0.0424 (5)	0.0031 (3)	0.0065 (3)	-0.0044 (4)
Cl4	0.0656 (7)	0.0881 (9)	0.0447 (6)	-0.0102 (6)	-0.0016 (5)	-0.0210 (6)
Cl5	0.1042 (19)	0.180 (3)	0.165 (3)	0.0196 (19)	0.0098 (18)	-0.052 (2)
Cl6	0.140 (2)	0.204 (3)	0.172 (3)	0.022 (2)	0.028 (2)	-0.086 (2)
Cl5'	0.130 (3)	0.197 (4)	0.188 (4)	0.016 (3)	0.011 (3)	-0.047 (3)
Cl6'	0.144 (3)	0.204 (4)	0.186 (4)	0.018 (3)	0.012 (3)	-0.053 (3)

Geometric parameters (Å, º) for **15e**

Rh1—C10	1.863 (3)	C31—H31	0.9500
Rh1—C1	2.060 (2)	C32—C33	1.383 (4)
Rh1—P1	2.3144 (7)	C32—H32	0.9500
Rh1—P2	2.3161 (6)	C33—C34	1.390 (4)
C1—N2	1.330 (3)	C33—H33	0.9500
C1—C9	1.435 (3)	C34—H34	0.9500
N2—N3	1.396 (3)	C35—C40	1.393 (4)
N2—C47	1.460 (3)	C35—C36	1.399 (4)
N3—C4	1.363 (3)	C36—C37	1.390 (4)
N3—C48	1.431 (3)	C36—H36	0.9500
C4—C5	1.400 (4)	C37—C38	1.381 (4)
C4—C9	1.405 (3)	C37—H37	0.9500
C5—C6	1.378 (4)	C38—C39	1.384 (4)
C5—H5	0.9500	C38—H38	0.9500
C6—C7	1.406 (5)	C39—C40	1.385 (4)
C6—H6	0.9500	C39—H39	0.9500
C7—C8	1.374 (4)	C40—H40	0.9500
C7—H7	0.9500	C41—C46	1.396 (4)
C8—C9	1.404 (4)	C41—C42	1.397 (4)
C8—H8	0.9500	C42—C43	1.391 (4)
C10—O10	1.145 (3)	C42—H42	0.9500
P1—C17	1.819 (3)	C43—C44	1.387 (5)
P1—C11	1.822 (3)	C43—H43	0.9500
P1—C23	1.830 (3)	C44—C45	1.381 (5)

C11—C16	1.390 (4)	C44—H44	0.9500
C11—C12	1.392 (4)	C45—C46	1.390 (4)
C12—C13	1.392 (4)	C45—H45	0.9500
C12—H12	0.9500	C46—H46	0.9500
C13—C14	1.382 (5)	C47—H47A	0.9800
C13—H13	0.9500	C47—H47B	0.9800
C14—C15	1.378 (5)	C47—H47C	0.9800
C14—H14	0.9500	C48—C53	1.382 (4)
C15—C16	1.391 (4)	C48—C49	1.390 (4)
C15—H15	0.9500	C49—C50	1.392 (4)
C16—H16	0.9500	C49—H49	0.9500
C17—C18	1.397 (4)	C50—C51	1.395 (4)
C17—C22	1.397 (4)	C50—H50	0.9500
C18—C19	1.389 (4)	C51—C52	1.384 (4)
C18—H18	0.9500	C51—I51	2.094 (2)
C19—C20	1.382 (4)	C52—C53	1.390 (4)
C19—H19	0.9500	C52—H52	0.9500
C20—C21	1.384 (4)	C53—H53	0.9500
C20—H20	0.9500	P3—F5	1.580 (2)
C21—C22	1.393 (4)	P3—F6	1.586 (2)
C21—H21	0.9500	P3—F3	1.5911 (19)
C22—H22	0.9500	P3—F4	1.594 (2)
C23—C28	1.394 (4)	P3—F1	1.5990 (18)
C23—C24	1.398 (4)	P3—F2	1.6006 (19)
C24—C25	1.387 (4)	C1C—Cl2	1.749 (3)
C24—H24	0.9500	C1C—Cl1	1.757 (3)
C25—C26	1.386 (4)	C1C—H1C1	0.9900
C25—H25	0.9500	C1C—H1C2	0.9900
C26—C27	1.382 (5)	C2C—Cl4	1.743 (3)
C26—H26	0.9500	C2C—Cl3	1.754 (4)
C27—C28	1.398 (4)	C2C—H2C1	0.9900
C27—H27	0.9500	C2C—H2C2	0.9900
C28—H28	0.9500	C3C—Cl6	1.702 (6)
P2—C35	1.823 (3)	C3C—Cl5	1.703 (6)
P2—C29	1.825 (2)	C3C—H3C1	0.9900
P2—C41	1.828 (2)	C3C—H3C2	0.9900
C29—C34	1.389 (4)	C3C'—Cl6'	1.728 (8)
C29—C30	1.399 (4)	C3C'—Cl5'	1.740 (8)
C30—C31	1.388 (4)	C3C'—H3C3	0.9900
C30—H30	0.9500	C3C'—H3C4	0.9900
C31—C32	1.382 (4)		

C10—Rh1—C1	172.27 (11)	C31—C32—C33	120.1 (3)
C10—Rh1—P1	90.88 (8)	C31—C32—H32	119.9
C1—Rh1—P1	89.85 (7)	C33—C32—H32	119.9
C10—Rh1—P2	91.17 (8)	C32—C33—C34	120.0 (3)
C1—Rh1—P2	88.66 (7)	C32—C33—H33	120.0
P1—Rh1—P2	175.50 (2)	C34—C33—H33	120.0
N2—C1—C9	105.0 (2)	C29—C34—C33	120.4 (3)
N2—C1—Rh1	127.90 (18)	C29—C34—H34	119.8
C9—C1—Rh1	127.07 (18)	C33—C34—H34	119.8
C1—N2—N3	112.6 (2)	C40—C35—C36	119.0 (2)
C1—N2—C47	127.2 (2)	C40—C35—P2	118.9 (2)
N3—N2—C47	120.2 (2)	C36—C35—P2	122.1 (2)
C4—N3—N2	106.5 (2)	C37—C36—C35	120.1 (3)
C4—N3—C48	127.9 (2)	C37—C36—H36	120.0
N2—N3—C48	124.6 (2)	C35—C36—H36	120.0
N3—C4—C5	129.8 (2)	C38—C37—C36	120.2 (3)
N3—C4—C9	108.0 (2)	C38—C37—H37	119.9
C5—C4—C9	122.1 (2)	C36—C37—H37	119.9
C6—C5—C4	116.4 (3)	C37—C38—C39	120.0 (3)
C6—C5—H5	121.8	C37—C38—H38	120.0
C4—C5—H5	121.8	C39—C38—H38	120.0
C5—C6—C7	122.2 (3)	C38—C39—C40	120.1 (3)
C5—C6—H6	118.9	C38—C39—H39	119.9
C7—C6—H6	118.9	C40—C39—H39	119.9
C8—C7—C6	121.3 (3)	C39—C40—C35	120.5 (3)
C8—C7—H7	119.4	C39—C40—H40	119.7
C6—C7—H7	119.4	C35—C40—H40	119.7
C7—C8—C9	117.9 (3)	C46—C41—C42	119.6 (2)
C7—C8—H8	121.1	C46—C41—P2	118.90 (19)
C9—C8—H8	121.1	C42—C41—P2	121.2 (2)
C8—C9—C4	120.1 (2)	C43—C42—C41	120.1 (3)
C8—C9—C1	132.1 (2)	C43—C42—H42	120.0
C4—C9—C1	107.8 (2)	C41—C42—H42	120.0
O10—C10—Rh1	176.0 (3)	C44—C43—C42	119.9 (3)
C17—P1—C11	105.56 (12)	C44—C43—H43	120.1
C17—P1—C23	102.98 (11)	C42—C43—H43	120.1
C11—P1—C23	102.24 (11)	C45—C44—C43	120.2 (3)
C17—P1—Rh1	113.96 (9)	C45—C44—H44	119.9
C11—P1—Rh1	112.17 (9)	C43—C44—H44	119.9
C23—P1—Rh1	118.45 (8)	C44—C45—C46	120.5 (3)

C16—C11—C12	119.9 (2)	C44—C45—H45	119.8
C16—C11—P1	122.4 (2)	C46—C45—H45	119.8
C12—C11—P1	117.7 (2)	C45—C46—C41	119.7 (3)
C11—C12—C13	119.6 (3)	C45—C46—H46	120.1
C11—C12—H12	120.2	C41—C46—H46	120.1
C13—C12—H12	120.2	N2—C47—H47A	109.5
C14—C13—C12	120.3 (3)	N2—C47—H47B	109.5
C14—C13—H13	119.9	H47A—C47—H47B	109.5
C12—C13—H13	119.9	N2—C47—H47C	109.5
C15—C14—C13	120.0 (3)	H47A—C47—H47C	109.5
C15—C14—H14	120.0	H47B—C47—H47C	109.5
C13—C14—H14	120.0	C53—C48—C49	120.8 (2)
C14—C15—C16	120.5 (3)	C53—C48—N3	118.5 (2)
C14—C15—H15	119.8	C49—C48—N3	120.6 (2)
C16—C15—H15	119.8	C48—C49—C50	119.6 (2)
C11—C16—C15	119.7 (3)	C48—C49—H49	120.2
C11—C16—H16	120.2	C50—C49—H49	120.2
C15—C16—H16	120.2	C49—C50—C51	119.3 (2)
C18—C17—C22	118.9 (2)	C49—C50—H50	120.3
C18—C17—P1	122.2 (2)	C51—C50—H50	120.3
C22—C17—P1	118.84 (19)	C52—C51—C50	120.7 (2)
C19—C18—C17	120.2 (3)	C52—C51—I51	118.90 (19)
C19—C18—H18	119.9	C50—C51—I51	120.38 (19)
C17—C18—H18	119.9	C51—C52—C53	119.7 (2)
C20—C19—C18	120.4 (3)	C51—C52—H52	120.1
C20—C19—H19	119.8	C53—C52—H52	120.1
C18—C19—H19	119.8	C48—C53—C52	119.8 (2)
C19—C20—C21	120.1 (3)	C48—C53—H53	120.1
C19—C20—H20	120.0	C52—C53—H53	120.1
C21—C20—H20	120.0	F5—P3—F6	179.68 (17)
C20—C21—C22	120.0 (3)	F5—P3—F3	89.93 (13)
C20—C21—H21	120.0	F6—P3—F3	89.83 (15)
C22—C21—H21	120.0	F5—P3—F4	90.53 (16)
C21—C22—C17	120.4 (3)	F6—P3—F4	89.71 (17)
C21—C22—H22	119.8	F3—P3—F4	179.50 (15)
C17—C22—H22	119.8	F5—P3—F1	90.19 (12)
C28—C23—C24	119.1 (2)	F6—P3—F1	90.02 (12)
C28—C23—P1	121.9 (2)	F3—P3—F1	89.34 (10)
C24—C23—P1	118.98 (19)	F4—P3—F1	90.46 (11)
C25—C24—C23	120.4 (3)	F5—P3—F2	90.50 (12)
C25—C24—H24	119.8	F6—P3—F2	89.29 (12)

C23—C24—H24	119.8	F3—P3—F2	90.24 (11)
C26—C25—C24	120.2 (3)	F4—P3—F2	89.95 (12)
C26—C25—H25	119.9	F1—P3—F2	179.19 (13)
C24—C25—H25	119.9	Cl2—C1C—Cl1	112.31 (18)
C27—C26—C25	119.9 (3)	Cl2—C1C—H1C1	109.1
C27—C26—H26	120.0	Cl1—C1C—H1C1	109.1
C25—C26—H26	120.0	Cl2—C1C—H1C2	109.1
C26—C27—C28	120.3 (3)	Cl1—C1C—H1C2	109.1
C26—C27—H27	119.9	H1C1—C1C—H1C2	107.9
C28—C27—H27	119.9	Cl4—C2C—Cl3	111.9 (2)
C23—C28—C27	120.0 (3)	Cl4—C2C—H2C1	109.2
C23—C28—H28	120.0	Cl3—C2C—H2C1	109.2
C27—C28—H28	120.0	Cl4—C2C—H2C2	109.2
C35—P2—C29	104.52 (11)	Cl3—C2C—H2C2	109.2
C35—P2—C41	104.99 (12)	H2C1—C2C—H2C2	107.9
C29—P2—C41	102.02 (11)	Cl6—C3C—Cl5	112.5 (5)
C35—P2—Rh1	113.76 (9)	Cl6—C3C—H3C1	109.1
C29—P2—Rh1	116.03 (9)	Cl5—C3C—H3C1	109.1
C41—P2—Rh1	114.14 (8)	Cl6—C3C—H3C2	109.1
C34—C29—C30	119.1 (2)	Cl5—C3C—H3C2	109.1
C34—C29—P2	120.33 (19)	H3C1—C3C—H3C2	107.8
C30—C29—P2	120.5 (2)	Cl6'—C3C'—Cl5'	107.5 (9)
C31—C30—C29	120.2 (3)	Cl6'—C3C'—H3C3	110.2
C31—C30—H30	119.9	Cl5'—C3C'—H3C3	110.2
C29—C30—H30	119.9	Cl6'—C3C'—H3C4	110.2
C32—C31—C30	120.1 (3)	Cl5'—C3C'—H3C4	110.2
C32—C31—H31	119.9	H3C3—C3C'—H3C4	108.5
C30—C31—H31	119.9		
C9—C1—N2—N3	1.4 (3)	C23—C24—C25—C26	0.7 (4)
Rh1—C1—N2—N3	-179.92 (17)	C24—C25—C26—C27	0.6 (5)
C9—C1—N2—C47	-179.1 (2)	C25—C26—C27—C28	-1.2 (5)
Rh1—C1—N2—C47	-0.4 (4)	C24—C23—C28—C27	0.9 (4)
C1—N2—N3—C4	-2.5 (3)	P1—C23—C28—C27	179.3 (2)
C47—N2—N3—C4	178.0 (2)	C26—C27—C28—C23	0.4 (5)
C1—N2—N3—C48	-172.2 (2)	C35—P2—C29—C34	-143.3 (2)
C47—N2—N3—C48	8.3 (4)	C41—P2—C29—C34	107.5 (2)
N2—N3—C4—C5	-177.2 (3)	Rh1—P2—C29—C34	-17.2 (2)
C48—N3—C4—C5	-7.9 (5)	C35—P2—C29—C30	40.6 (2)
N2—N3—C4—C9	2.4 (3)	C41—P2—C29—C30	-68.5 (2)
C48—N3—C4—C9	171.7 (2)	Rh1—P2—C29—C30	166.77 (18)

N3—C4—C5—C6	-179.6 (3)	C34—C29—C30—C31	0.5 (4)
C9—C4—C5—C6	0.8 (5)	P2—C29—C30—C31	176.6 (2)
C4—C5—C6—C7	0.0 (6)	C29—C30—C31—C32	-0.2 (4)
C5—C6—C7—C8	-0.3 (6)	C30—C31—C32—C33	-0.5 (4)
C6—C7—C8—C9	-0.2 (5)	C31—C32—C33—C34	0.8 (4)
C7—C8—C9—C4	1.0 (4)	C30—C29—C34—C33	-0.3 (4)
C7—C8—C9—C1	-178.1 (3)	P2—C29—C34—C33	-176.4 (2)
N3—C4—C9—C8	179.0 (3)	C32—C33—C34—C29	-0.4 (4)
C5—C4—C9—C8	-1.3 (4)	C29—P2—C35—C40	100.7 (2)
N3—C4—C9—C1	-1.7 (3)	C41—P2—C35—C40	-152.3 (2)
C5—C4—C9—C1	178.0 (3)	Rh1—P2—C35—C40	-26.8 (2)
N2—C1—C9—C8	179.4 (3)	C29—P2—C35—C36	-78.5 (2)
Rh1—C1—C9—C8	0.7 (4)	C41—P2—C35—C36	28.5 (2)
N2—C1—C9—C4	0.2 (3)	Rh1—P2—C35—C36	153.96 (19)
Rh1—C1—C9—C4	-178.52 (18)	C40—C35—C36—C37	0.6 (4)
C17—P1—C11—C16	-23.8 (2)	P2—C35—C36—C37	179.8 (2)
C23—P1—C11—C16	83.6 (2)	C35—C36—C37—C38	-0.4 (4)
Rh1—P1—C11—C16	-148.5 (2)	C36—C37—C38—C39	-0.7 (5)
C17—P1—C11—C12	158.8 (2)	C37—C38—C39—C40	1.7 (5)
C23—P1—C11—C12	-93.8 (2)	C38—C39—C40—C35	-1.5 (5)
Rh1—P1—C11—C12	34.1 (2)	C36—C35—C40—C39	0.4 (4)
C16—C11—C12—C13	-0.5 (4)	P2—C35—C40—C39	-178.9 (2)
P1—C11—C12—C13	177.1 (2)	C35—P2—C41—C46	-146.9 (2)
C11—C12—C13—C14	0.4 (4)	C29—P2—C41—C46	-38.1 (2)
C12—C13—C14—C15	-0.4 (5)	Rh1—P2—C41—C46	87.8 (2)
C13—C14—C15—C16	0.3 (5)	C35—P2—C41—C42	39.0 (2)
C12—C11—C16—C15	0.4 (4)	C29—P2—C41—C42	147.8 (2)
P1—C11—C16—C15	-177.0 (2)	Rh1—P2—C41—C42	-86.3 (2)
C14—C15—C16—C11	-0.3 (4)	C46—C41—C42—C43	1.2 (4)
C11—P1—C17—C18	76.2 (2)	P2—C41—C42—C43	175.3 (2)
C23—P1—C17—C18	-30.7 (2)	C41—C42—C43—C44	-1.9 (4)
Rh1—P1—C17—C18	-160.29 (19)	C42—C43—C44—C45	1.1 (4)
C11—P1—C17—C22	-102.5 (2)	C43—C44—C45—C46	0.3 (4)
C23—P1—C17—C22	150.7 (2)	C44—C45—C46—C41	-1.0 (4)
Rh1—P1—C17—C22	21.1 (2)	C42—C41—C46—C45	0.2 (4)
C22—C17—C18—C19	-0.5 (4)	P2—C41—C46—C45	-174.0 (2)
P1—C17—C18—C19	-179.2 (2)	C4—N3—C48—C53	-50.7 (4)
C17—C18—C19—C20	0.5 (4)	N2—N3—C48—C53	116.7 (3)
C18—C19—C20—C21	0.4 (4)	C4—N3—C48—C49	126.0 (3)
C19—C20—C21—C22	-1.2 (4)	N2—N3—C48—C49	-66.5 (3)
C20—C21—C22—C17	1.2 (4)	C53—C48—C49—C50	-1.0 (4)

C18—C17—C22—C21	-0.3 (4)	N3—C48—C49—C50	-177.7 (2)
P1—C17—C22—C21	178.4 (2)	C48—C49—C50—C51	0.9 (4)
C17—P1—C23—C28	127.3 (2)	C49—C50—C51—C52	-0.5 (4)
C11—P1—C23—C28	17.9 (3)	C49—C50—C51—I51	178.7 (2)
Rh1—P1—C23—C28	-105.9 (2)	C50—C51—C52—C53	0.2 (4)
C17—P1—C23—C24	-54.3 (2)	I51—C51—C52—C53	-178.99 (19)
C11—P1—C23—C24	-163.6 (2)	C49—C48—C53—C52	0.7 (4)
Rh1—P1—C23—C24	72.5 (2)	N3—C48—C53—C52	177.4 (2)
C28—C23—C24—C25	-1.4 (4)	C51—C52—C53—C48	-0.3 (4)
P1—C23—C24—C25	-179.9 (2)		

Crystal structure determination of 2-chloro-*N*-(2-((1-(2-chlorophenyl)-1,2-dihydroquinazolidin-4-yl)(methylimino)methyl)phenyl)aniline (16b)

Crystal data for **16b**

$C_{28}H_{22}Cl_2N_4$	F(000) = 1008
M_r = 485.39	D_x = 1.364 Mg m^{-3}
Monoclinic, $P2_1/n$ *(no.14)*	Mo $K\alpha$ radiation, λ = 0.71073 Å
a = 14.690 (1) Å	Cell parameters from 1000 reflections
b = 7.488 (1) Å	θ = 2.5–25.0°
c = 21.484 (2) Å	μ = 0.30 mm^{-1}
β = 90.75 (1)°	T = 123 K
V = 2363.0 (4) Å^3	Blocks, colourless
Z = 4	0.30 × 0.24 × 0.06 mm

Data collection for **16b**

Bruker-Nonius KappaCCD diffractometer	4414 reflections with $I > 2\sigma(I)$
Radiation source: fine-focus sealed tube	R_{int} = 0.034
rotation in ϕ and ω, 1° scans	θ_{max} = 27.5°, θ_{min} = 2.8°
Absorption correction: multi-scan *SADABS* (Sheldrick,2008)	h = -19→18
T_{min} = 0.890, T_{max} = 0.980	k = -9→9
36379 measured reflections	l = -27→27
5408 independent reflections	

Refinement for **16b**

Refinement on F^2	Primary atom site location: structure-invariant direct methods
Least-squares matrix: full	Secondary atom site location: difference Fourier map
$R[F^2 > 2\sigma(F^2)] = 0.040$	Hydrogen site location: difference Fourier map
$wR(F^2) = 0.093$	H atoms treated by a mixture of independent and constrained refinement
$S = 1.05$	$w = 1/[\sigma^2(F_o^2) + (0.034P)^2 + 1.5695P]$ where $P = (F_o^2 + 2F_c^2)/3$
5408 reflections	$(\Delta/\sigma)_{max} < 0.001$
311 parameters	$\Delta\rangle_{max} = 0.33$ e Å^{-3}
1 restraint	$\Delta\rangle_{min} = -0.28$ e Å^{-3}

Fractional atomic coordinates and isotropic or equivalent isotropic displacement parameters (Å^2) for **16b**

	x	*y*	*z*	U_{iso}*/U_{eq}
N1	0.63119 (9)	0.58952 (18)	0.25227 (6)	0.0214 (3)
C2	0.71075 (11)	0.6380 (2)	0.21592 (8)	0.0239 (3)
H2A	0.7402	0.5281	0.2004	0.029*
H2B	0.7552	0.7012	0.2430	0.029*
N3	0.68613 (9)	0.75256 (18)	0.16316 (6)	0.0207 (3)
C4	0.63655 (10)	0.9043 (2)	0.17807 (7)	0.0181 (3)
C5	0.63965 (11)	1.0639 (2)	0.14454 (7)	0.0234 (3)
H5	0.6779	1.0745	0.1094	0.028*
C6	0.58648 (12)	1.2066 (2)	0.16300 (8)	0.0302 (4)
H6	0.5894	1.3157	0.1406	0.036*
C7	0.52899 (12)	1.1934 (2)	0.21365 (9)	0.0304 (4)
H7	0.4935	1.2930	0.2260	0.036*
C8	0.52389 (11)	1.0342 (2)	0.24587 (8)	0.0248 (3)
H8	0.4836	1.0237	0.2799	0.030*
C9	0.57747 (10)	0.8890 (2)	0.22888 (7)	0.0190 (3)
C10	0.57150 (10)	0.7128 (2)	0.25818 (7)	0.0191 (3)
C11	0.48892 (10)	0.6683 (2)	0.29679 (7)	0.0195 (3)
C12	0.40897 (10)	0.59824 (19)	0.26219 (7)	0.0179 (3)
C13	0.40998 (11)	0.6008 (2)	0.19675 (7)	0.0201 (3)
H13	0.4615	0.6492	0.1765	0.024*
C14	0.33881 (11)	0.5356 (2)	0.16095 (7)	0.0235 (3)
H14	0.3408	0.5410	0.1168	0.028*
C15	0.26401 (11)	0.4619 (2)	0.19056 (8)	0.0254 (3)
H15	0.2149	0.4153	0.1663	0.031*

C16	0.26023 (11)	0.4555 (2)	0.25452 (8)	0.0244 (3)
H16	0.2089	0.4031	0.2738	0.029*
C17	0.33108 (11)	0.5253 (2)	0.29172 (7)	0.0201 (3)
N18	0.32800 (9)	0.5194 (2)	0.35575 (6)	0.0252 (3)
H18	0.3771 (11)	0.553 (3)	0.3748 (8)	0.030*
C19	0.25501 (11)	0.4604 (2)	0.39205 (7)	0.0216 (3)
C20	0.16707 (11)	0.5281 (2)	0.38486 (8)	0.0274 (4)
H20	0.1543	0.6115	0.3526	0.033*
C21	0.09813 (12)	0.4753 (3)	0.42416 (9)	0.0325 (4)
H21	0.0382	0.5203	0.4179	0.039*
C22	0.11581 (12)	0.3575 (3)	0.47243 (8)	0.0322 (4)
H22	0.0686	0.3243	0.4999	0.039*
C23	0.20243 (12)	0.2881 (2)	0.48050 (8)	0.0277 (4)
H23	0.2153	0.2077	0.5137	0.033*
C24	0.26995 (11)	0.3371 (2)	0.43975 (7)	0.0229 (3)
Cl24	0.37621 (3)	0.23662 (7)	0.44731 (2)	0.03576 (12)
N25	0.48930 (9)	0.6890 (2)	0.35613 (6)	0.0260 (3)
C26	0.56886 (12)	0.7683 (3)	0.38736 (8)	0.0348 (4)
H26A	0.6160	0.7927	0.3566	0.052*
H26B	0.5927	0.6851	0.4188	0.052*
H26C	0.5511	0.8801	0.4075	0.052*
C27	0.74494 (10)	0.7531 (2)	0.11119 (7)	0.0208 (3)
C28	0.83548 (12)	0.8060 (3)	0.11748 (9)	0.0322 (4)
H28	0.8586	0.8424	0.1570	0.039*
C29	0.89198 (13)	0.8059 (3)	0.06642 (10)	0.0395 (5)
H29	0.9534	0.8440	0.0710	0.047*
C30	0.85980 (13)	0.7508 (3)	0.00893 (9)	0.0361 (4)
H30	0.8994	0.7489	-0.0257	0.043*
C31	0.77014 (13)	0.6984 (2)	0.00167 (8)	0.0304 (4)
H31	0.7476	0.6615	-0.0379	0.037*
C32	0.71344 (11)	0.7001 (2)	0.05268 (7)	0.0229 (3)
Cl32	0.60044 (3)	0.63461 (7)	0.04329 (2)	0.03596 (12)

Atomic displacement parameters (Å^2) for **16b**

	U^{11}	U^{22}	U^{33}	U^{12}	U^{13}	U^{23}
N1	0.0216 (6)	0.0219 (7)	0.0208 (7)	0.0021 (5)	0.0021 (5)	0.0027 (5)
C2	0.0213 (8)	0.0245 (8)	0.0260 (8)	0.0066 (6)	0.0038 (6)	0.0054 (7)
N3	0.0204 (6)	0.0222 (7)	0.0196 (6)	0.0046 (5)	0.0048 (5)	0.0022 (5)
C4	0.0162 (7)	0.0193 (7)	0.0187 (7)	0.0000 (6)	-0.0010 (6)	-0.0017 (6)
C5	0.0238 (8)	0.0235 (8)	0.0228 (8)	-0.0022 (6)	0.0002 (6)	0.0026 (6)
C6	0.0366 (10)	0.0190 (8)	0.0350 (10)	0.0002 (7)	-0.0044 (8)	0.0041 (7)
C7	0.0323 (9)	0.0200 (8)	0.0389 (10)	0.0064 (7)	0.0011 (8)	-0.0050 (7)
C8	0.0234 (8)	0.0241 (8)	0.0270 (9)	0.0025 (6)	0.0036 (7)	-0.0044 (7)
C9	0.0191 (7)	0.0175 (7)	0.0205 (8)	-0.0006 (6)	-0.0004 (6)	-0.0014 (6)
C10	0.0194 (7)	0.0210 (8)	0.0168 (7)	-0.0010 (6)	0.0004 (6)	-0.0014 (6)
C11	0.0211 (7)	0.0171 (7)	0.0205 (8)	0.0010 (6)	0.0032 (6)	0.0010 (6)
C12	0.0203 (7)	0.0141 (7)	0.0195 (7)	0.0018 (6)	0.0016 (6)	0.0009 (6)
C13	0.0226 (7)	0.0169 (7)	0.0208 (8)	0.0020 (6)	0.0032 (6)	0.0006 (6)
C14	0.0300 (9)	0.0222 (8)	0.0183 (8)	0.0024 (7)	-0.0014 (6)	0.0001 (6)
C15	0.0273 (8)	0.0228 (8)	0.0261 (8)	-0.0032 (7)	-0.0054 (7)	-0.0027 (6)
C16	0.0244 (8)	0.0225 (8)	0.0264 (8)	-0.0044 (6)	0.0016 (6)	0.0009 (6)
C17	0.0233 (8)	0.0171 (7)	0.0198 (7)	0.0002 (6)	0.0017 (6)	0.0016 (6)
N18	0.0217 (7)	0.0345 (8)	0.0194 (7)	-0.0084 (6)	0.0013 (5)	0.0031 (6)
C19	0.0221 (8)	0.0241 (8)	0.0188 (8)	-0.0054 (6)	0.0034 (6)	-0.0021 (6)
C20	0.0262 (8)	0.0274 (9)	0.0285 (9)	-0.0006 (7)	0.0008 (7)	-0.0007 (7)
C21	0.0216 (8)	0.0401 (10)	0.0360 (10)	0.0020 (7)	0.0047 (7)	-0.0091 (8)
C22	0.0278 (9)	0.0430 (11)	0.0261 (9)	-0.0108 (8)	0.0105 (7)	-0.0085 (8)
C23	0.0321 (9)	0.0325 (9)	0.0185 (8)	-0.0095 (7)	0.0038 (7)	-0.0004 (7)
C24	0.0210 (8)	0.0271 (8)	0.0205 (8)	-0.0038 (6)	0.0004 (6)	-0.0033 (6)
Cl24	0.0256 (2)	0.0478 (3)	0.0338 (2)	0.00270 (19)	-0.00103 (17)	0.0119 (2)
N25	0.0233 (7)	0.0349 (8)	0.0198 (7)	-0.0043 (6)	0.0017 (5)	-0.0017 (6)
C26	0.0296 (9)	0.0527 (12)	0.0220 (8)	-0.0103 (8)	0.0010 (7)	-0.0068 (8)
C27	0.0186 (7)	0.0209 (8)	0.0228 (8)	0.0018 (6)	0.0047 (6)	0.0001 (6)
C28	0.0221 (8)	0.0422 (11)	0.0325 (9)	-0.0026 (8)	0.0019 (7)	-0.0050 (8)
C29	0.0209 (9)	0.0483 (12)	0.0494 (12)	-0.0029 (8)	0.0112 (8)	-0.0014 (9)
C30	0.0379 (10)	0.0345 (10)	0.0365 (10)	0.0059 (8)	0.0215 (8)	0.0027 (8)
C31	0.0429 (10)	0.0267 (9)	0.0219 (8)	0.0034 (8)	0.0069 (7)	0.0000 (7)
C32	0.0229 (8)	0.0207 (8)	0.0250 (8)	0.0007 (6)	0.0006 (6)	0.0014 (6)
Cl32	0.0280 (2)	0.0449 (3)	0.0349 (2)	-0.00758 (19)	-0.00601 (18)	-0.0022 (2)

Geometric parameters (Å, º) for **16b**

N1—C10	1.281 (2)	C16—H16	0.9500
N1—C2	1.460 (2)	C17—N18	1.378 (2)
C2—N3	1.4629 (19)	N18—C19	1.405 (2)
C2—H2A	0.9900	N18—H18	0.862 (14)
C2—H2B	0.9900	C19—C24	1.394 (2)
N3—C4	1.3891 (19)	C19—C20	1.394 (2)
N3—C27	1.4206 (19)	C20—C21	1.385 (2)
C4—C5	1.397 (2)	C20—H20	0.9500
C4—C9	1.408 (2)	C21—C22	1.383 (3)
C5—C6	1.384 (2)	C21—H21	0.9500
C5—H5	0.9500	C22—C23	1.383 (3)
C6—C7	1.390 (3)	C22—H22	0.9500
C6—H6	0.9500	C23—C24	1.381 (2)
C7—C8	1.381 (2)	C23—H23	0.9500
C7—H7	0.9500	C24—Cl24	1.7386 (17)
C8—C9	1.394 (2)	N25—C26	1.466 (2)
C8—H8	0.9500	C26—H26A	0.9800
C9—C10	1.465 (2)	C26—H26B	0.9800
C10—C11	1.516 (2)	C26—H26C	0.9800
C11—N25	1.284 (2)	C27—C32	1.392 (2)
C11—C12	1.478 (2)	C27—C28	1.393 (2)
C12—C13	1.406 (2)	C28—C29	1.384 (3)
C12—C17	1.424 (2)	C28—H28	0.9500
C13—C14	1.379 (2)	C29—C30	1.380 (3)
C13—H13	0.9500	C29—H29	0.9500
C14—C15	1.391 (2)	C30—C31	1.381 (3)
C14—H14	0.9500	C30—H30	0.9500
C15—C16	1.377 (2)	C31—C32	1.385 (2)
C15—H15	0.9500	C31—H31	0.9500
C16—C17	1.405 (2)	C32—Cl32	1.7400 (17)
C10—N1—C2	115.31 (13)	N18—C17—C16	121.48 (14)
N1—C2—N3	111.64 (12)	N18—C17—C12	119.62 (14)
N1—C2—H2A	109.3	C16—C17—C12	118.86 (14)
N3—C2—H2A	109.3	C17—N18—C19	126.83 (14)
N1—C2—H2B	109.3	C17—N18—H18	115.2 (13)
N3—C2—H2B	109.3	C19—N18—H18	117.9 (13)
H2A—C2—H2B	108.0	C24—C19—C20	117.30 (14)
C4—N3—C27	120.33 (13)	C24—C19—N18	120.15 (14)

C4—N3—C2	115.20 (12)	C20—C19—N18	122.45 (15)
C27—N3—C2	117.63 (12)	C21—C20—C19	120.83 (17)
N3—C4—C5	124.08 (14)	C21—C20—H20	119.6
N3—C4—C9	116.27 (13)	C19—C20—H20	119.6
C5—C4—C9	119.60 (14)	C22—C21—C20	120.50 (17)
C6—C5—C4	119.36 (15)	C22—C21—H21	119.8
C6—C5—H5	120.3	C20—C21—H21	119.8
C4—C5—H5	120.3	C23—C22—C21	119.79 (16)
C5—C6—C7	121.37 (16)	C23—C22—H22	120.1
C5—C6—H6	119.3	C21—C22—H22	120.1
C7—C6—H6	119.3	C24—C23—C22	119.19 (16)
C8—C7—C6	119.43 (16)	C24—C23—H23	120.4
C8—C7—H7	120.3	C22—C23—H23	120.4
C6—C7—H7	120.3	C23—C24—C19	122.31 (16)
C7—C8—C9	120.52 (15)	C23—C24—Cl24	118.49 (13)
C7—C8—H8	119.7	C19—C24—Cl24	119.18 (12)
C9—C8—H8	119.7	C11—N25—C26	119.76 (14)
C8—C9—C4	119.69 (14)	N25—C26—H26A	109.5
C8—C9—C10	123.57 (14)	N25—C26—H26B	109.5
C4—C9—C10	116.58 (13)	H26A—C26—H26B	109.5
N1—C10—C9	124.17 (14)	N25—C26—H26C	109.5
N1—C10—C11	116.72 (13)	H26A—C26—H26C	109.5
C9—C10—C11	119.09 (13)	H26B—C26—H26C	109.5
N25—C11—C12	122.35 (14)	C32—C27—C28	118.34 (15)
N25—C11—C10	121.58 (14)	C32—C27—N3	120.71 (14)
C12—C11—C10	116.06 (13)	C28—C27—N3	120.95 (15)
C13—C12—C17	118.01 (14)	C29—C28—C27	120.31 (17)
C13—C12—C11	118.64 (13)	C29—C28—H28	119.8
C17—C12—C11	123.35 (13)	C27—C28—H28	119.8
C14—C13—C12	122.33 (15)	C30—C29—C28	120.50 (18)
C14—C13—H13	118.8	C30—C29—H29	119.7
C12—C13—H13	118.8	C28—C29—H29	119.7
C13—C14—C15	118.89 (15)	C29—C30—C31	120.06 (16)
C13—C14—H14	120.6	C29—C30—H30	120.0
C15—C14—H14	120.6	C31—C30—H30	120.0
C16—C15—C14	120.84 (15)	C30—C31—C32	119.38 (17)
C16—C15—H15	119.6	C30—C31—H31	120.3
C14—C15—H15	119.6	C32—C31—H31	120.3
C15—C16—C17	121.03 (15)	C31—C32—C27	121.39 (16)
C15—C16—H16	119.5	C31—C32—Cl32	119.23 (13)
C17—C16—H16	119.5	C27—C32—Cl32	119.38 (12)

C10—N1—C2—N3	-37.58 (19)	C15—C16—C17—C12	-2.0 (2)
N1—C2—N3—C4	53.30 (18)	C13—C12—C17—N18	179.53 (14)
N1—C2—N3—C27	-155.49 (13)	C11—C12—C17—N18	0.5 (2)
C27—N3—C4—C5	0.3 (2)	C13—C12—C17—C16	1.6 (2)
C2—N3—C4—C5	150.66 (15)	C11—C12—C17—C16	-177.41 (14)
C27—N3—C4—C9	177.63 (14)	C16—C17—N18—C19	-5.5 (3)
C2—N3—C4—C9	-32.00 (19)	C12—C17—N18—C19	176.65 (15)
N3—C4—C5—C6	179.19 (15)	C17—N18—C19—C24	130.25 (18)
C9—C4—C5—C6	1.9 (2)	C17—N18—C19—C20	-53.5 (2)
C4—C5—C6—C7	-1.0 (3)	C24—C19—C20—C21	0.6 (2)
C5—C6—C7—C8	-0.7 (3)	N18—C19—C20—C21	-175.75 (16)
C6—C7—C8—C9	1.6 (3)	C19—C20—C21—C22	1.7 (3)
C7—C8—C9—C4	-0.6 (2)	C20—C21—C22—C23	-1.8 (3)
C7—C8—C9—C10	-175.90 (15)	C21—C22—C23—C24	-0.4 (3)
N3—C4—C9—C8	-178.59 (14)	C22—C23—C24—C19	2.8 (3)
C5—C4—C9—C8	-1.1 (2)	C22—C23—C24—Cl24	-175.56 (13)
N3—C4—C9—C10	-3.0 (2)	C20—C19—C24—C23	-2.8 (2)
C5—C4—C9—C10	174.46 (14)	N18—C19—C24—C23	173.60 (15)
C2—N1—C10—C9	3.0 (2)	C20—C19—C24—Cl24	175.49 (12)
C2—N1—C10—C11	-178.75 (13)	N18—C19—C24—Cl24	-8.1 (2)
C8—C9—C10—N1	-165.54 (16)	C12—C11—N25—C26	-176.64 (15)
C4—C9—C10—N1	19.1 (2)	C10—C11—N25—C26	4.1 (2)
C8—C9—C10—C11	16.2 (2)	C4—N3—C27—C32	-91.12 (19)
C4—C9—C10—C11	-159.19 (14)	C2—N3—C27—C32	119.20 (17)
N1—C10—C11—N25	86.67 (19)	C4—N3—C27—C28	89.07 (19)
C9—C10—C11—N25	-94.95 (19)	C2—N3—C27—C28	-60.6 (2)
N1—C10—C11—C12	-92.65 (17)	C32—C27—C28—C29	0.2 (3)
C9—C10—C11—C12	85.74 (17)	N3—C27—C28—C29	-179.98 (17)
N25—C11—C12—C13	172.11 (15)	C27—C28—C29—C30	-1.0 (3)
C10—C11—C12—C13	-8.6 (2)	C28—C29—C30—C31	1.2 (3)
N25—C11—C12—C17	-8.9 (2)	C29—C30—C31—C32	-0.6 (3)
C10—C11—C12—C17	170.43 (14)	C30—C31—C32—C27	-0.2 (3)
C17—C12—C13—C14	0.0 (2)	C30—C31—C32—Cl32	179.79 (14)
C11—C12—C13—C14	179.03 (14)	C28—C27—C32—C31	0.4 (2)
C12—C13—C14—C15	-1.2 (2)	N3—C27—C32—C31	-179.44 (15)
C13—C14—C15—C16	0.7 (2)	C28—C27—C32—Cl32	-179.58 (13)
C14—C15—C16—C17	0.9 (3)	N3—C27—C32—Cl32	0.6 (2)
C15—C16—C17—N18	-179.92 (16)		

Hydrogen-bond geometry (Å, º) for **16b**

D—H···*A*	*D*—H	H···*A*	*D*···*A*	*D*—H···*A*
N18—H18···N25	0.86 (1)	1.98 (2)	2.6882 (19)	138 (2)
C26—H26*B*···Cl24^{i}	0.98	2.97	3.6324 (18)	126
C28—H28···N1^{ii}	0.95	2.69	3.541 (2)	149
C23—H23···Cl32^{iii}	0.95	3.14	3.7597 (18)	125
C26—H26*A*···N1	0.98	2.72	3.335 (2)	121

Symmetry codes: (i) -*x*+1, -*y*+1, -*z*+1; (ii) -*x*+3/2, *y*+1/2, -*z*+1/2; (iii) *x*-1/2, -*y*+1/2, *z*+1/2.

Computing details for the structure elucidations

Data collection: Collect; cell refinement: *EVALCCD*; data reduction: *EVALCCD*; program(s) used to solve structure: *SHELXS*; program(s) used to refine structure: *SHELXL2013* (Sheldrick, 2013); molecular graphics: *SHELXTL-Plus*; software used to prepare material for publication: *publCIF*.

NMR Spectra

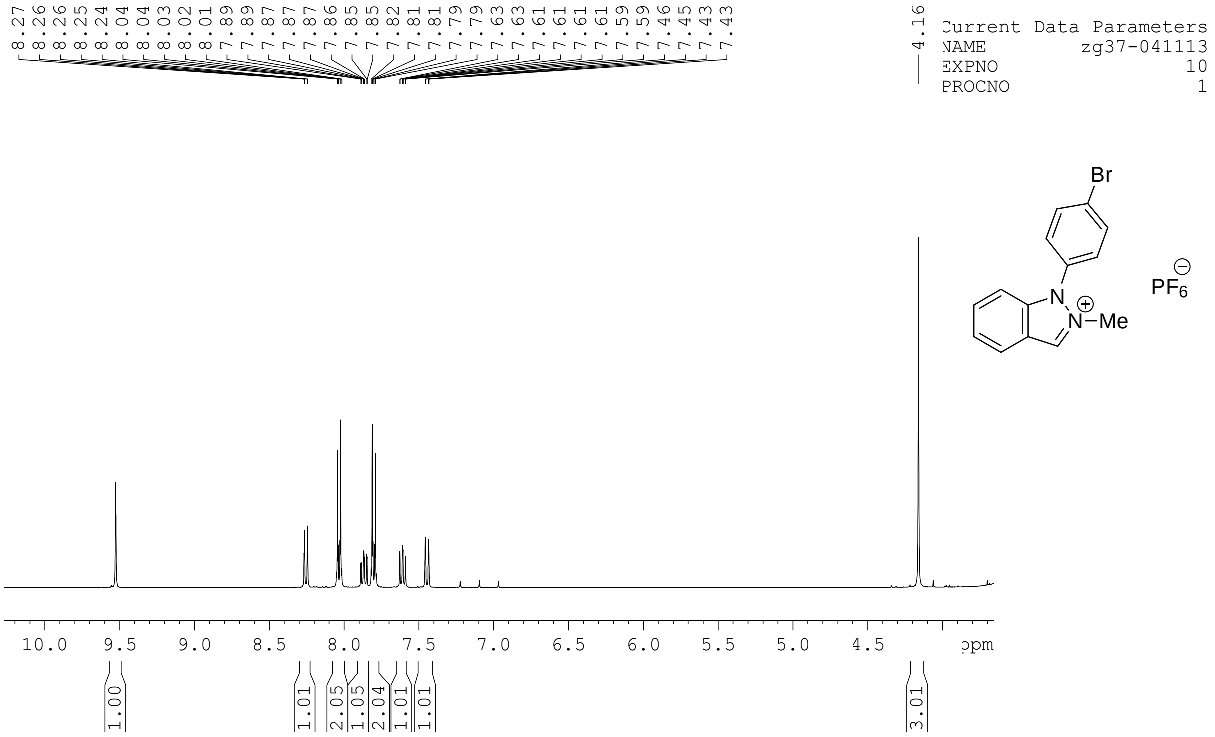

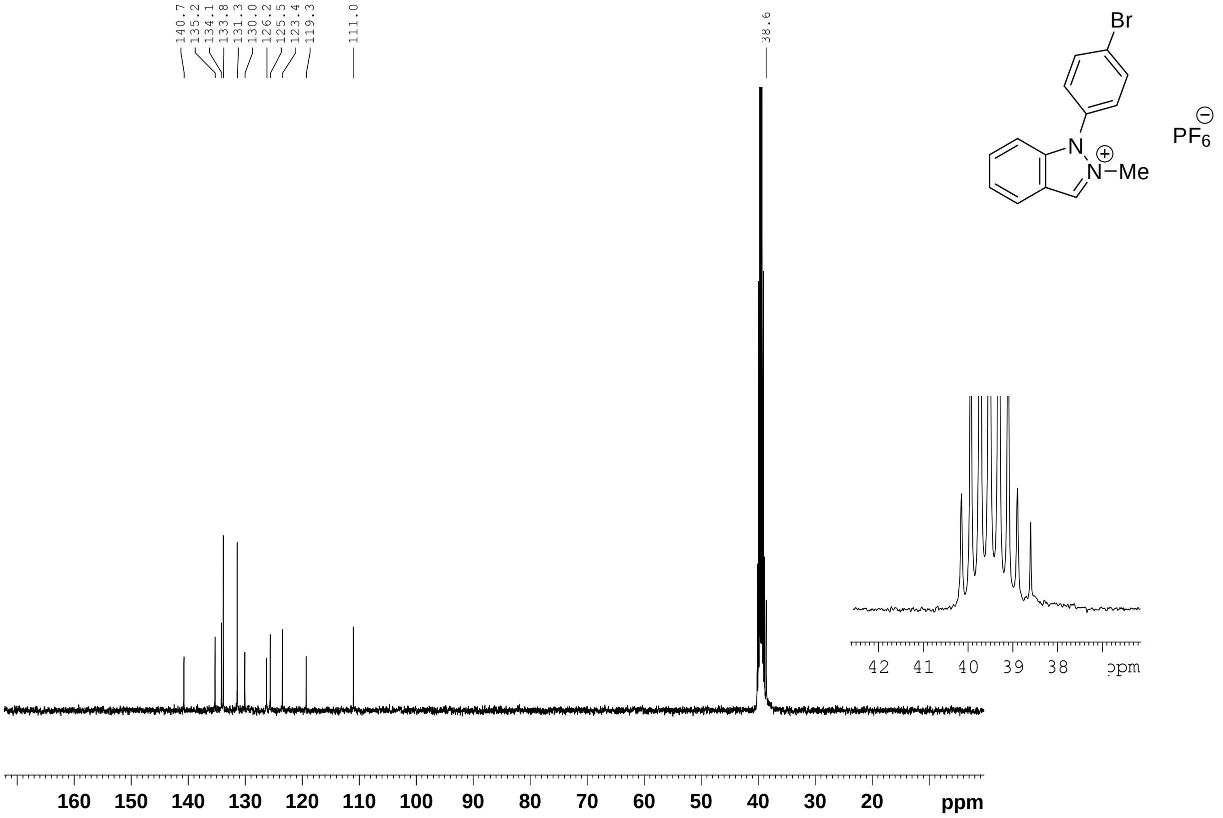
140.7
135.2
134.1
133.8
131.3
130.0
126.2
125.5
123.4
119.3
111.0
38.6
42 41 40 39 38 ppm
160 150 140 130 120 110 100 90 80 70 60 50 40 30 20 ppm
Br
N
N
Me
PF6

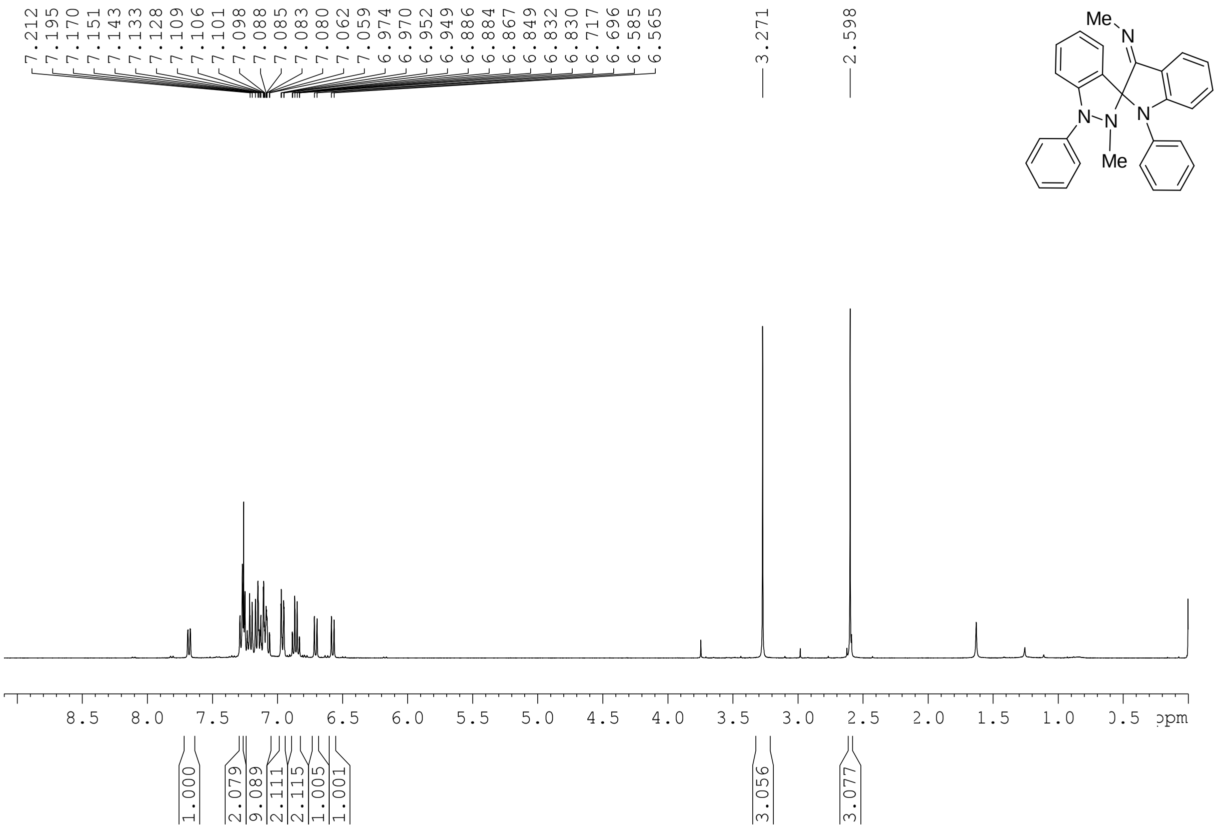
7.212
7.195
7.170
7.151
7.143
7.133
7.128
7.109
7.106
7.101
7.098
7.088
7.085
7.083
7.080
7.062
7.059
6.974
6.970
6.952
6.949
6.886
6.884
6.867
6.849
6.832
6.830
6.717
6.696
6.585
6.565
3.271
2.598
Me
N
N
N
N
Me
8.5 8.0 7.5 7.0 6.5 6.0 5.5 5.0 4.5 4.0 3.5 3.0 2.5 2.0 1.5 1.0 0.5 ppm
1.000
2.079
9.089
2.111
2.115
1.005
1.001
3.056
3.077

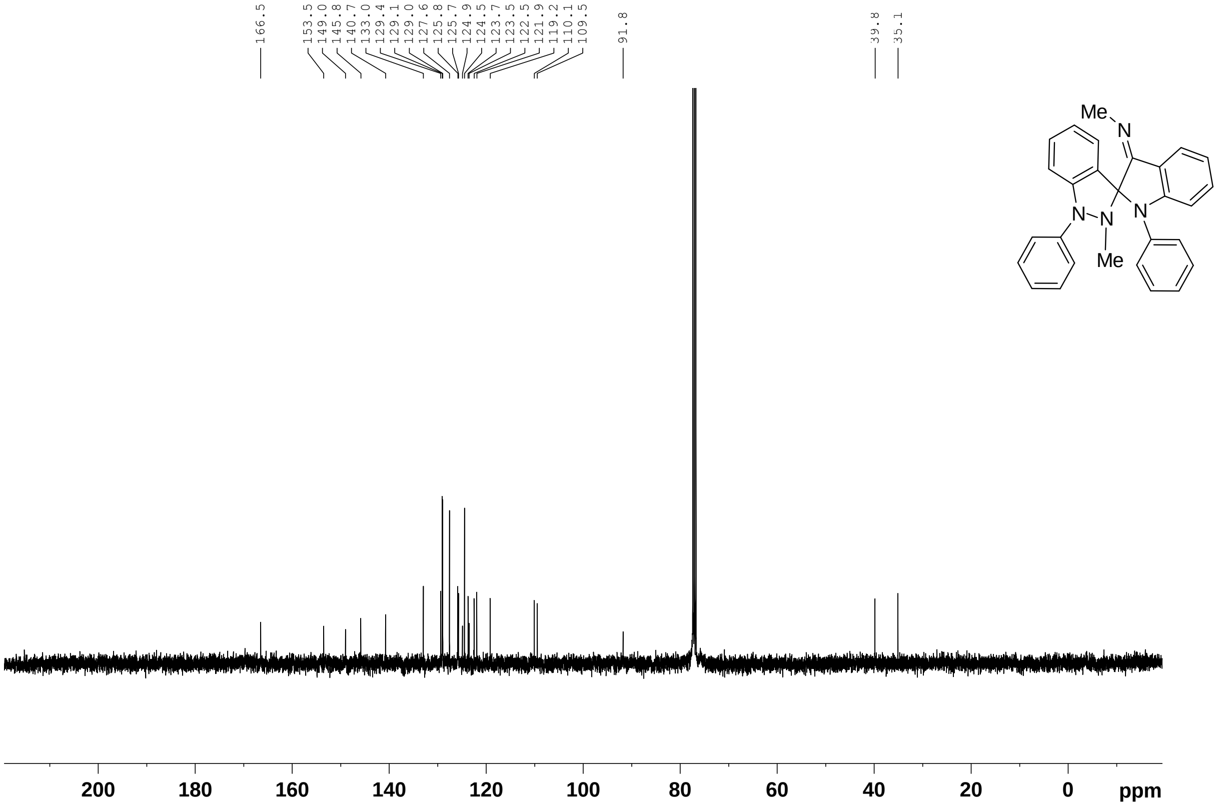

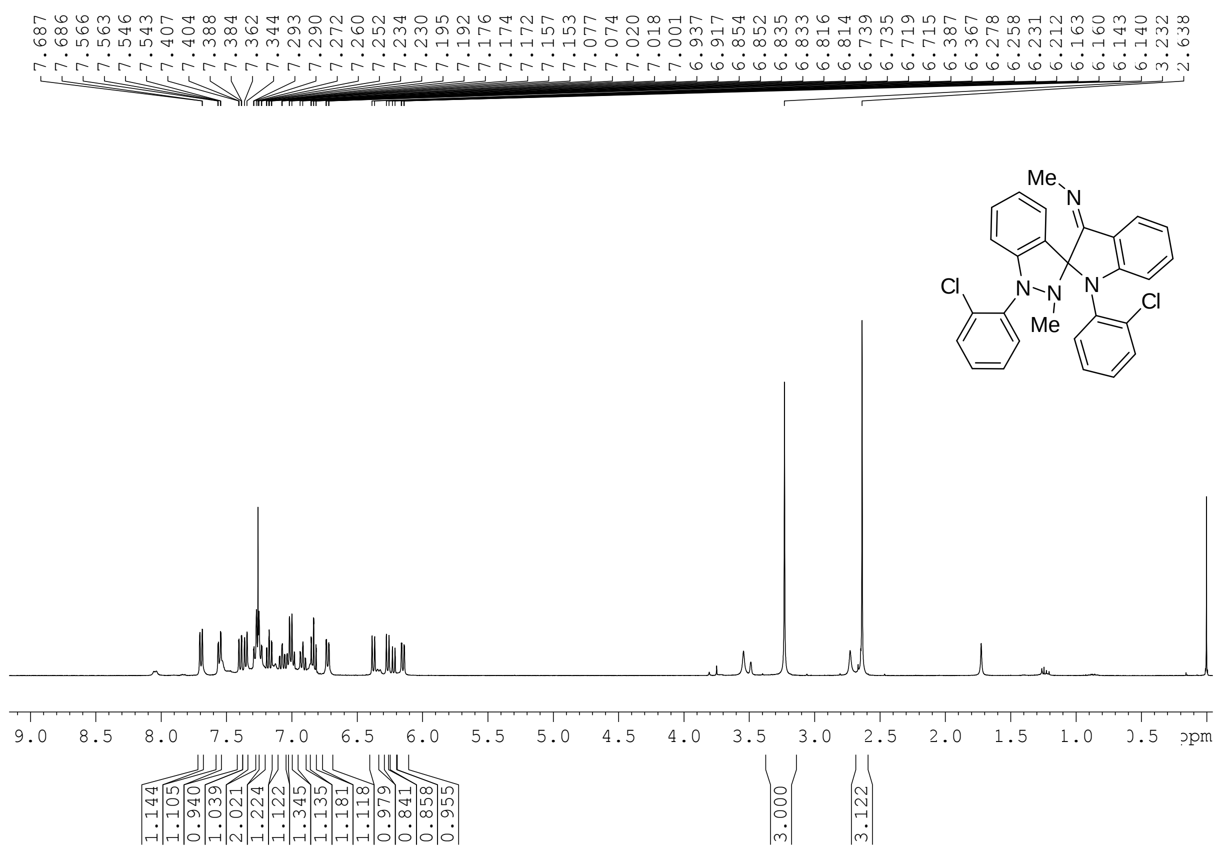

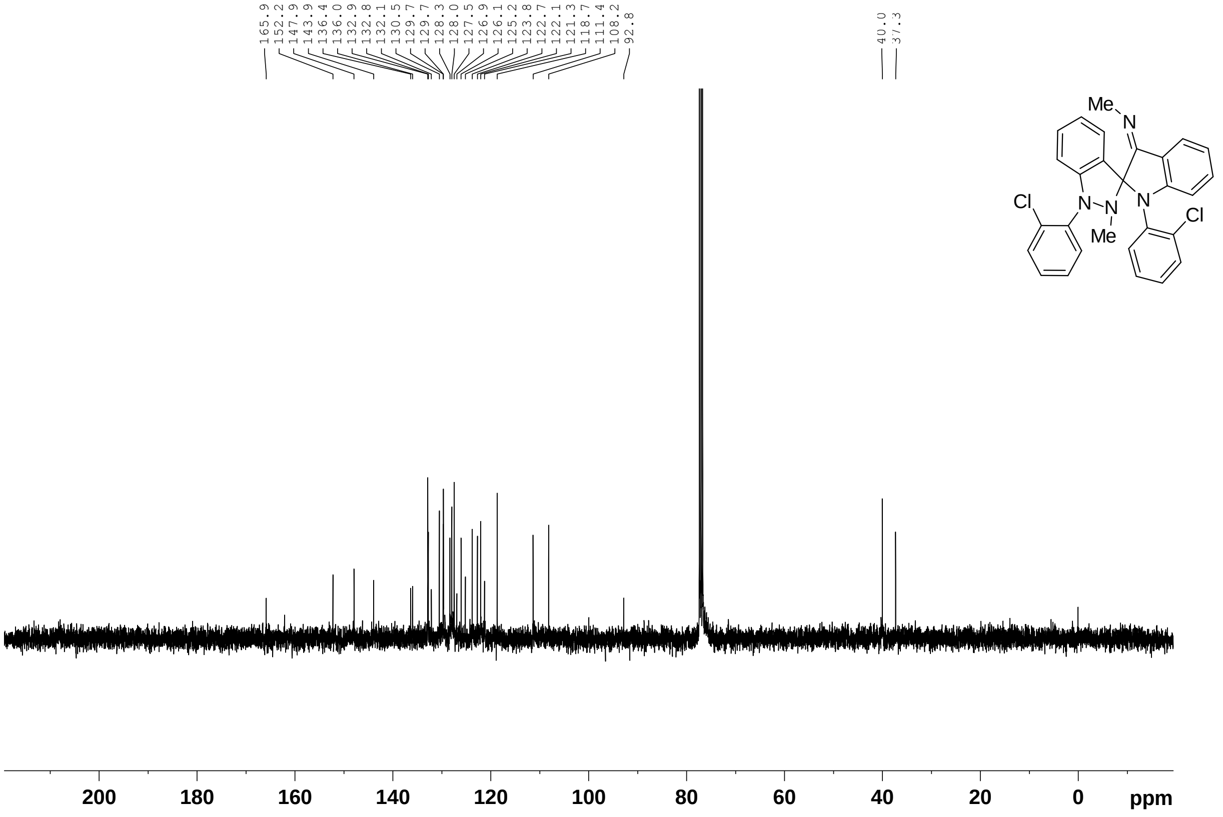
165.9
152.2
147.9
143.9
136.4
136.0
132.9
132.8
132.1
130.5
129.7
129.7
128.3
128.0
127.5
126.9
126.1
125.2
123.8
122.7
122.1
121.3
118.7
111.4
108.2
92.8
40.0
37.3
Me
N
Cl
N
N
N
Me
Cl
200
180
160
140
120
100
80
60
40
20
0
ppm

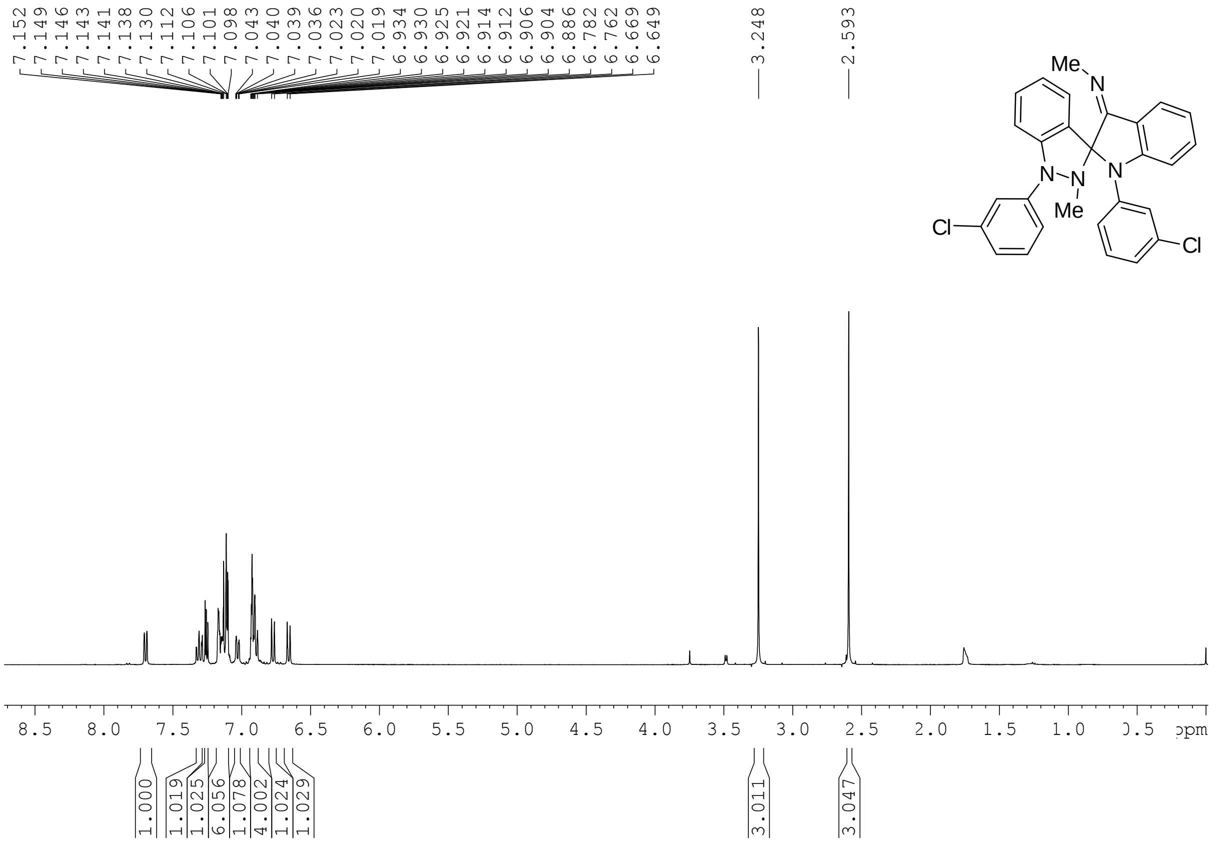
7.152
7.149
7.146
7.143
7.141
7.138
7.130
7.112
7.106
7.101
7.098
7.043
7.040
7.039
7.036
7.023
7.020
7.019
6.934
6.930
6.925
6.921
6.914
6.912
6.906
6.904
6.886
6.782
6.762
6.669
6.649
3.248
2.593
Me
N
N
N
N
Me
Cl
Cl
8.5
8.0
7.5
7.0
6.5
6.0
5.5
5.0
4.5
4.0
3.5
3.0
2.5
2.0
1.5
1.0
1.000
1.019
1.025
6.056
1.078
4.002
1.024
1.029
3.011
3.047

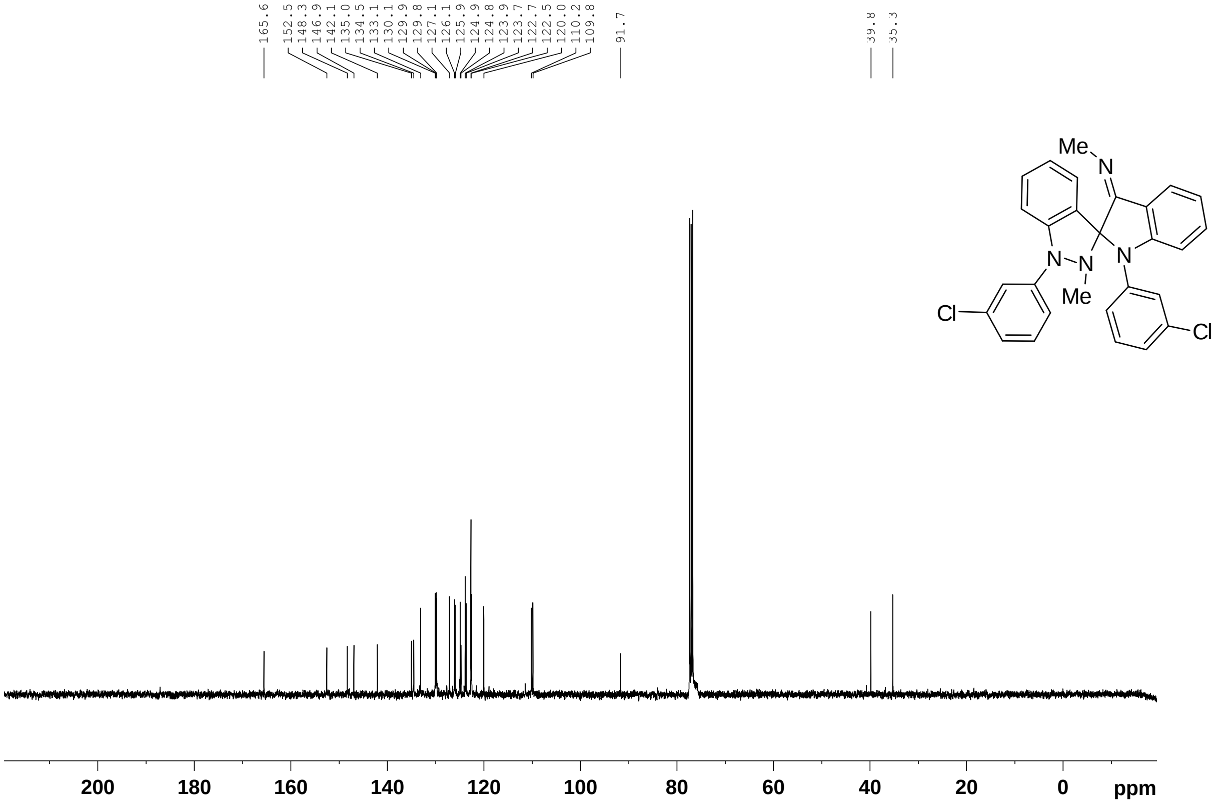
165.6
152.5
148.3
146.9
142.1
135.0
134.5
133.1
130.1
129.9
129.8
127.1
126.1
125.9
124.9
124.8
123.9
123.7
122.7
122.5
120.0
110.2
109.8
91.7
39.8
35.3
Me
N
N
N
Me
N
Cl
Cl
200
180
160
140
120
100
80
60
40
20
0
ppm

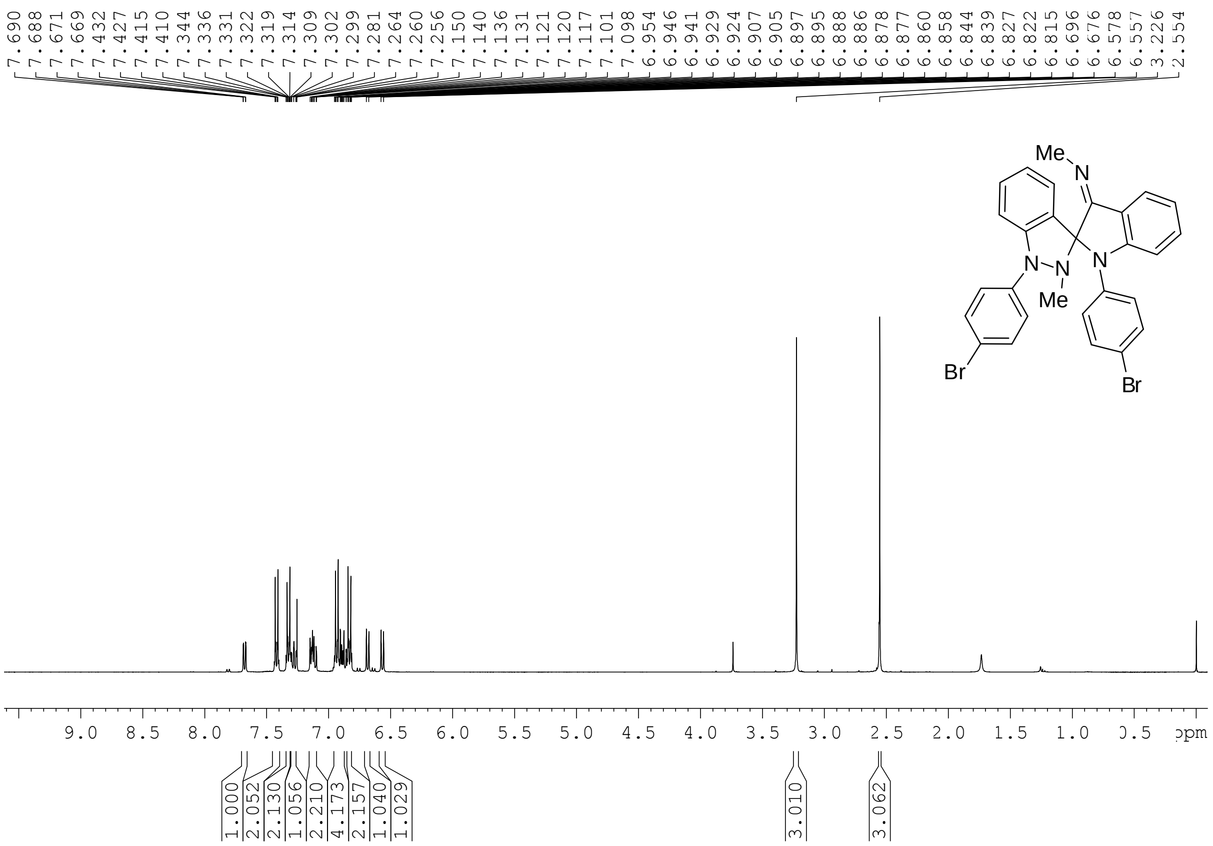
7.690
7.688
7.671
7.669
7.432
7.427
7.415
7.410
7.344
7.336
7.331
7.322
7.319
7.314
7.309
7.302
7.299
7.281
7.264
7.260
7.256
7.150
7.140
7.136
7.131
7.121
7.120
7.117
7.101
7.098
6.954
6.946
6.941
6.929
6.924
6.907
6.905
6.897
6.895
6.888
6.886
6.878
6.877
6.860
6.858
6.844
6.839
6.827
6.822
6.815
6.696
6.676
6.578
6.557
3.226
2.554
Me
N
N
N
Me
N
Br
Br
9.0
8.5
8.0
7.5
7.0
6.5
6.0
5.5
5.0
4.5
4.0
3.5
3.0
2.5
2.0
1.5
1.0
0.5
ppm
1.000
2.052
2.130
1.056
2.210
4.173
2.157
1.040
1.029
3.010
3.062

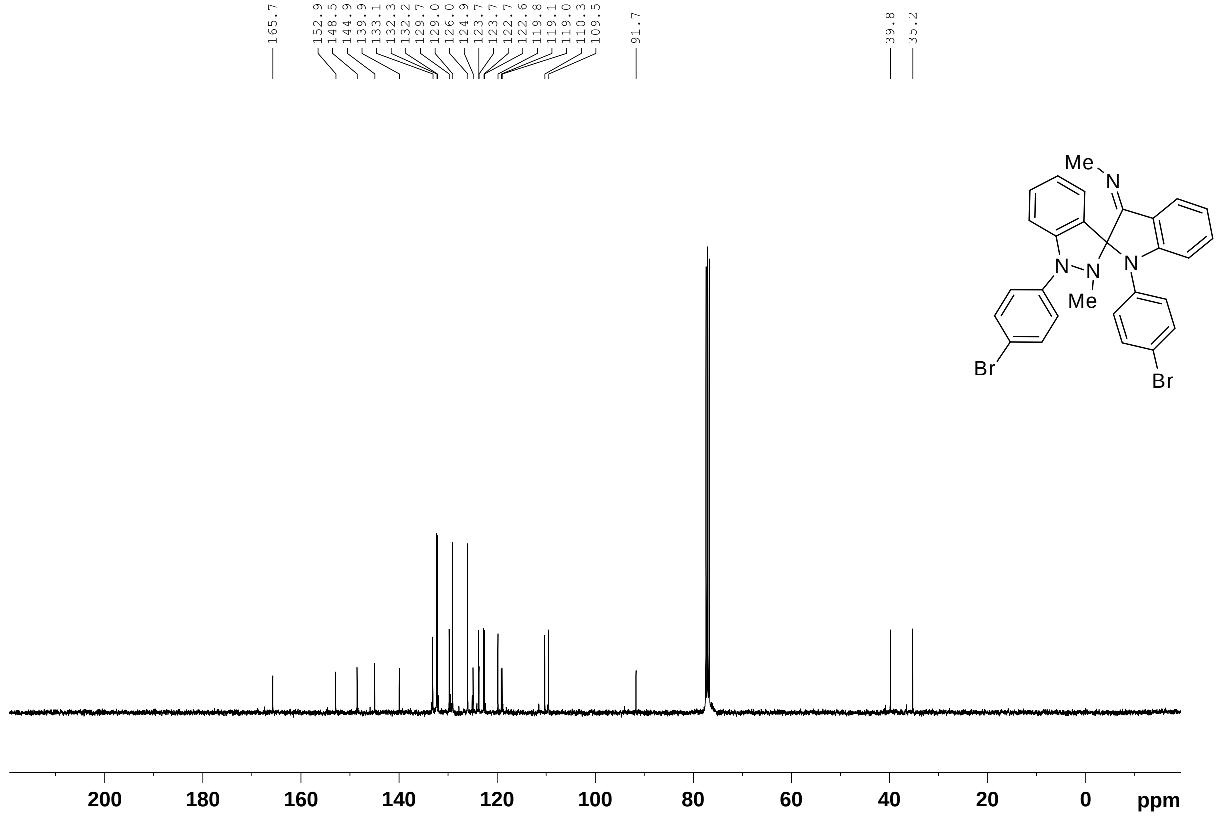
165.7
152.9
148.5
144.9
139.9
133.1
132.3
132.2
129.7
129.0
126.0
124.9
123.7
123.7
122.7
122.6
119.8
119.1
119.0
110.3
109.5
91.7
39.8
35.2
Me
N
N
N
N
Me
Br
Br
200
180
160
140
120
100
80
60
40
20
0
ppm

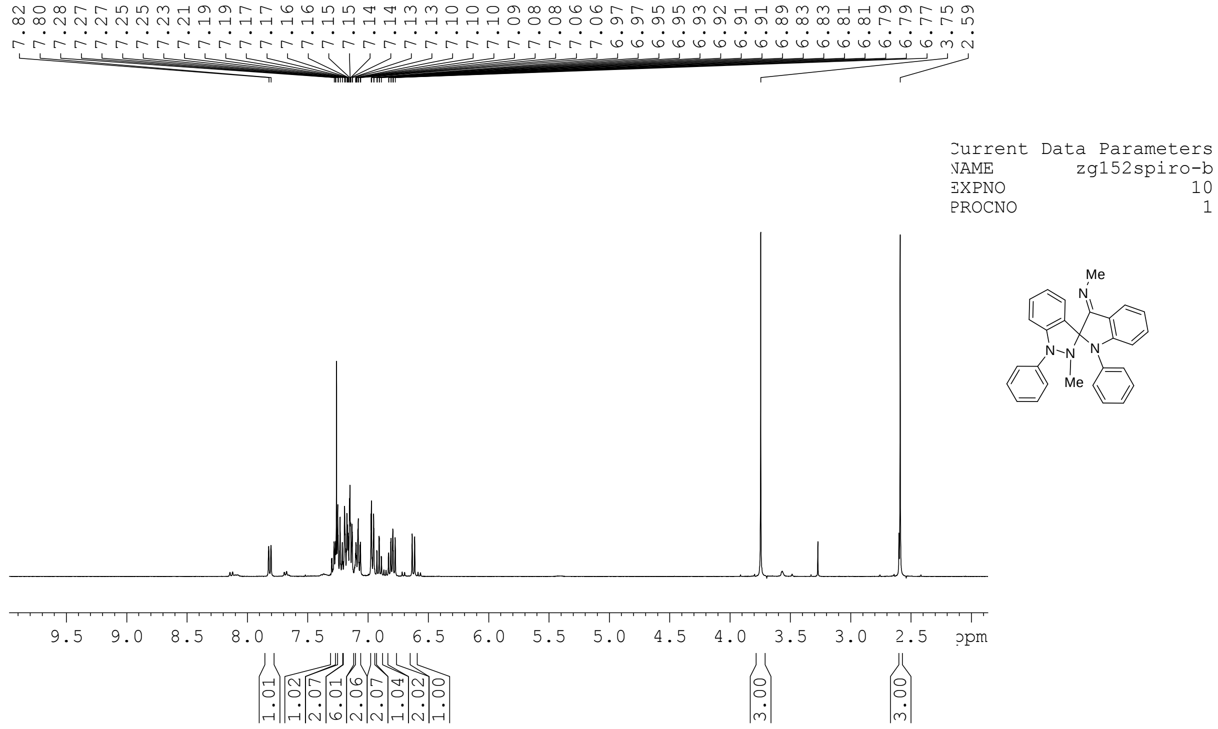
7.82
7.80
7.28
7.27
7.27
7.25
7.25
7.23
7.21
7.19
7.19
7.17
7.17
7.16
7.16
7.15
7.15
7.14
7.14
7.13
7.13
7.10
7.10
7.09
7.08
7.08
7.06
7.06
6.97
6.95
6.95
6.93
6.92
6.91
6.91
6.89
6.83
6.81
6.81
6.79
6.77
3.75
2.59
Current Data Parameters
NAME zg152spiro-b
EXPNO 10
PROCNO 1
Me
N
N
N
N
Me
9.5
9.0
8.5
8.0
7.5
7.0
6.5
6.0
5.5
5.0
4.5
4.0
3.5
3.0
2.5
ppm
1.01
1.02
2.07
6.01
2.06
2.07
1.04
2.02
1.00
3.00
3.00

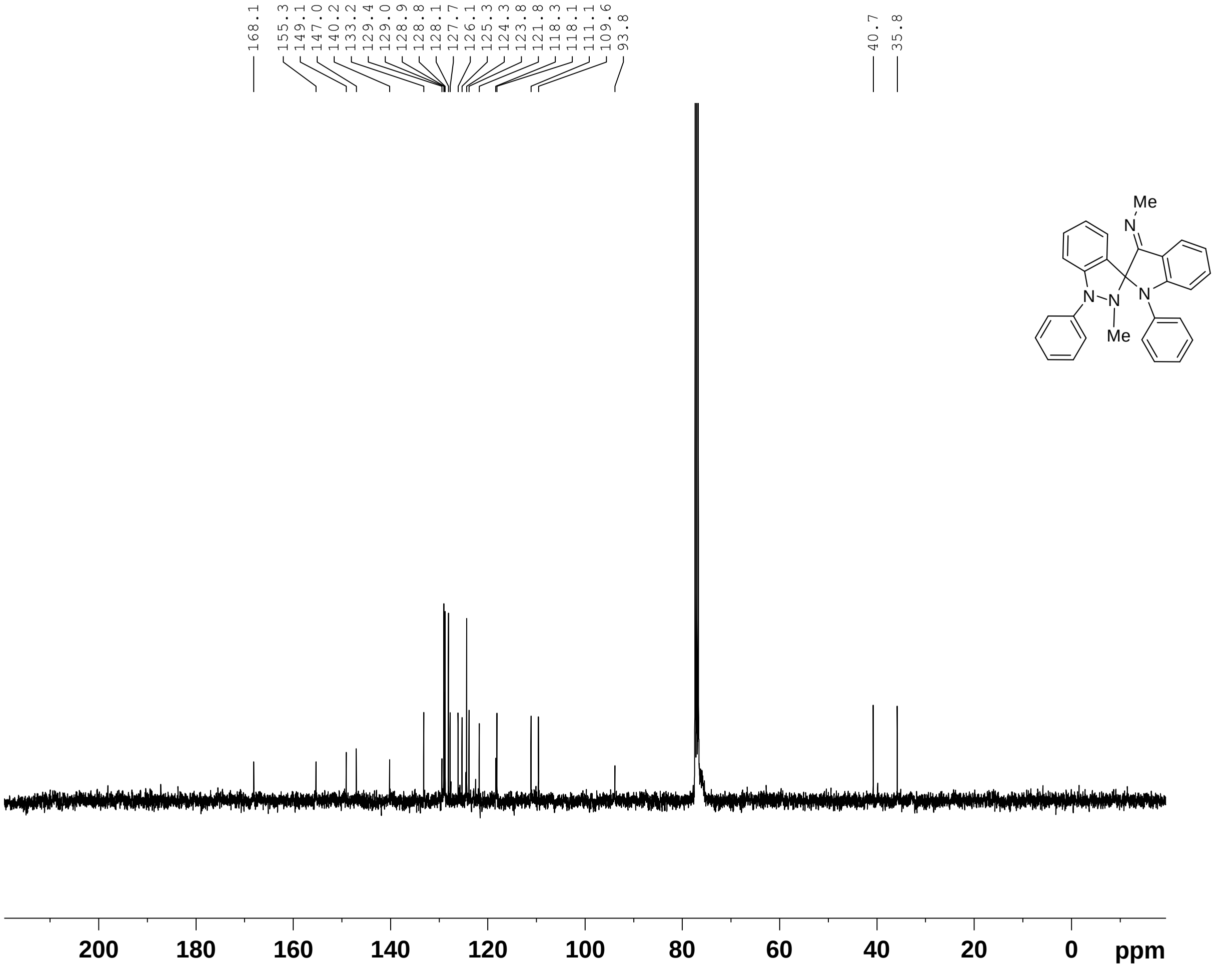
168.1
155.3
149.1
147.0
140.2
133.2
129.4
129.0
128.9
128.8
128.1
127.7
126.1
125.3
124.3
123.8
121.8
118.3
118.1
111.1
109.6
93.8
40.7
35.8
Me
N
N
N
N
Me
200
180
160
140
120
100
80
60
40
20
0
ppm

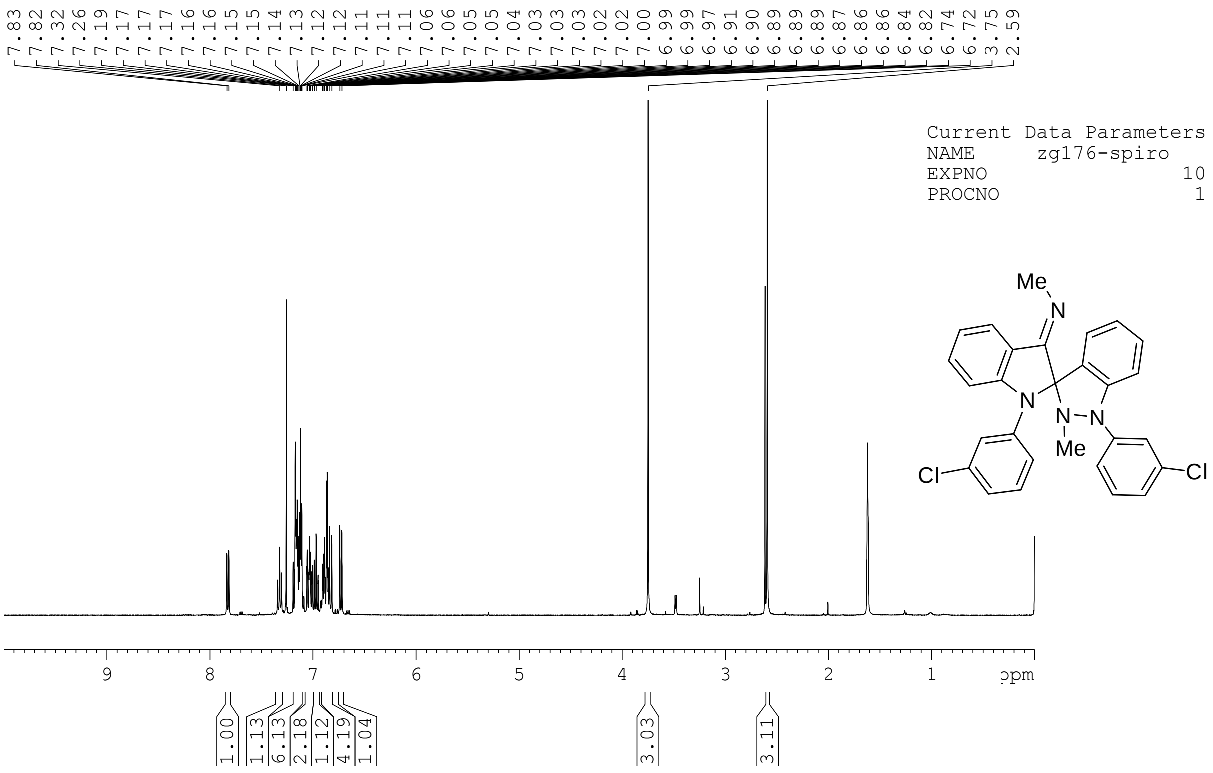

7.83 7.82 7.32 7.26 7.19 7.17 7.17 7.17 7.16 7.16 7.15 7.15 7.14 7.13 7.12 7.12 7.11 7.11 7.11 7.06 7.06 7.05 7.05 7.04 7.03 7.03 7.03 7.02 7.02 7.00 6.99 6.99 6.97 6.91 6.90 6.89 6.89 6.89 6.87 6.86 6.86 6.84 6.82 6.74 6.72 3.75 2.59
Current Data Parameters
NAME zg176-spiro
EXPNO 10
PROCNO 1
Me
N
N
N
N
Me
Cl
Cl
9 8 7 6 5 4 3 2 1 ppm
1.00 1.13 6.13 2.18 1.12 4.19 1.04 3.03 3.11

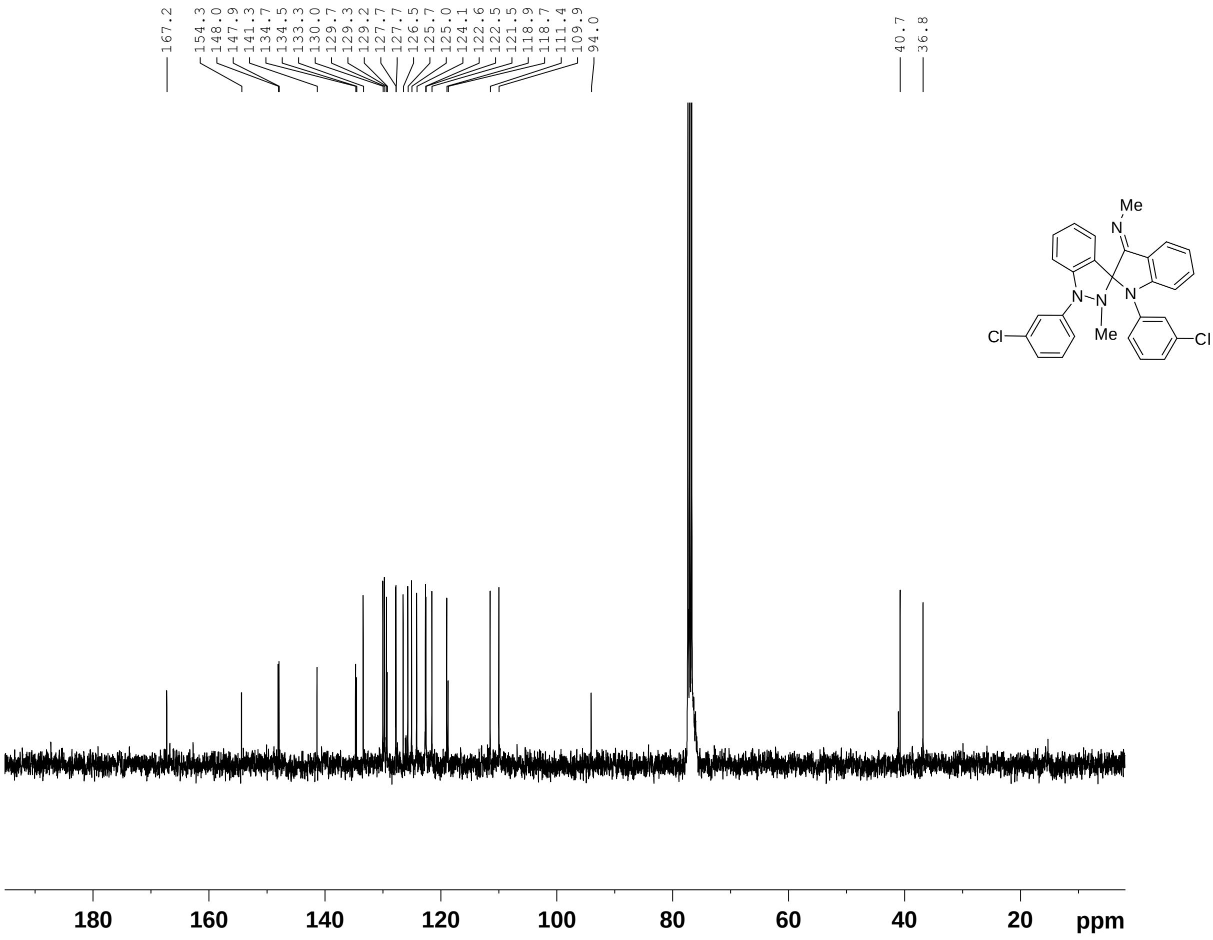

167.2 154.3 148.0 147.9 141.3 134.7 134.5 133.3 130.0 129.7 129.3 129.2 127.7 127.7 126.5 125.7 125.0 124.1 122.6 122.5 121.5 118.9 118.7 111.4 109.9 94.0 40.7 36.8
Me
N
N
N
N
Me
Cl
Cl
180 160 140 120 100 80 60 40 20 ppm

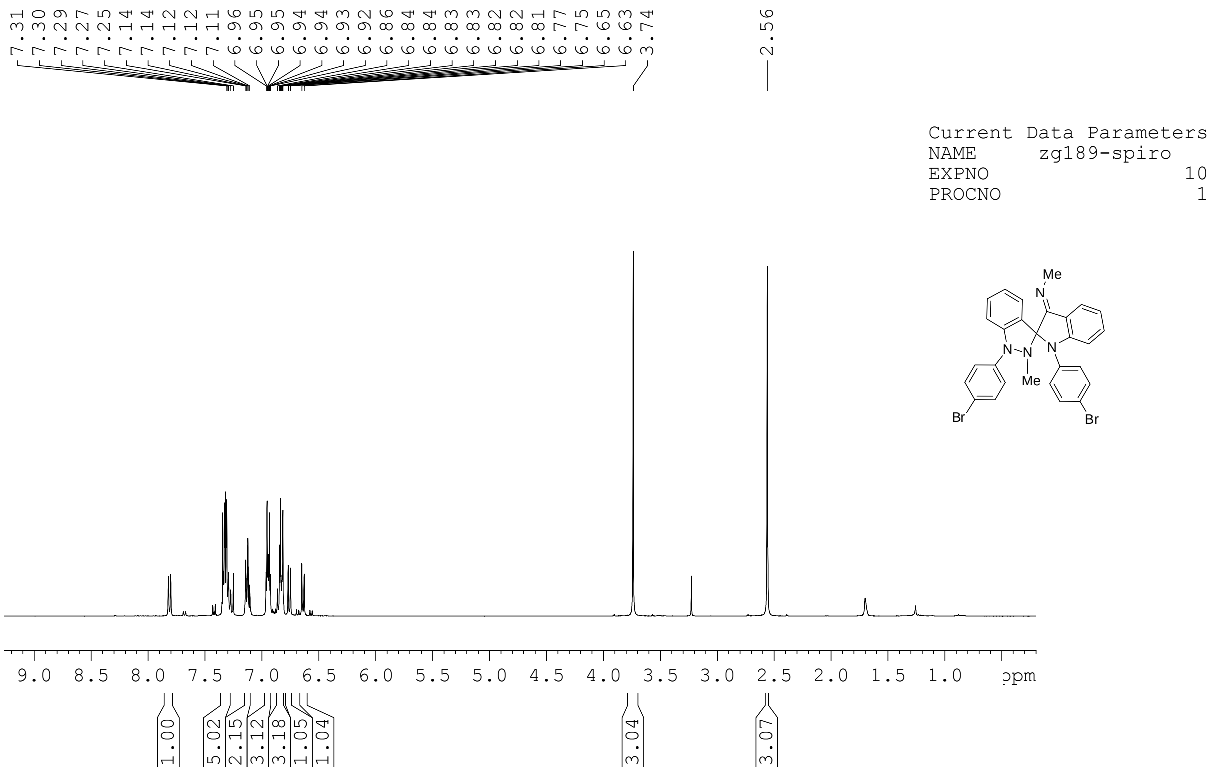
7.31 7.30 7.29 7.27 7.25 7.14 7.14 7.12 7.12 7.11 6.96 6.95 6.95 6.94 6.94 6.93 6.92 6.86 6.84 6.84 6.83 6.83 6.82 6.82 6.81 6.77 6.75 6.65 6.63 3.74
2.56
Current Data Parameters
NAME zg189-spiro
EXPNO 10
PROCNO 1
9.0 8.5 8.0 7.5 7.0 6.5 6.0 5.5 5.0 4.5 4.0 3.5 3.0 2.5 2.0 1.5 1.0 ppm
1.00 5.02 2.15 3.12 3.18 1.05 1.04 3.04 3.07

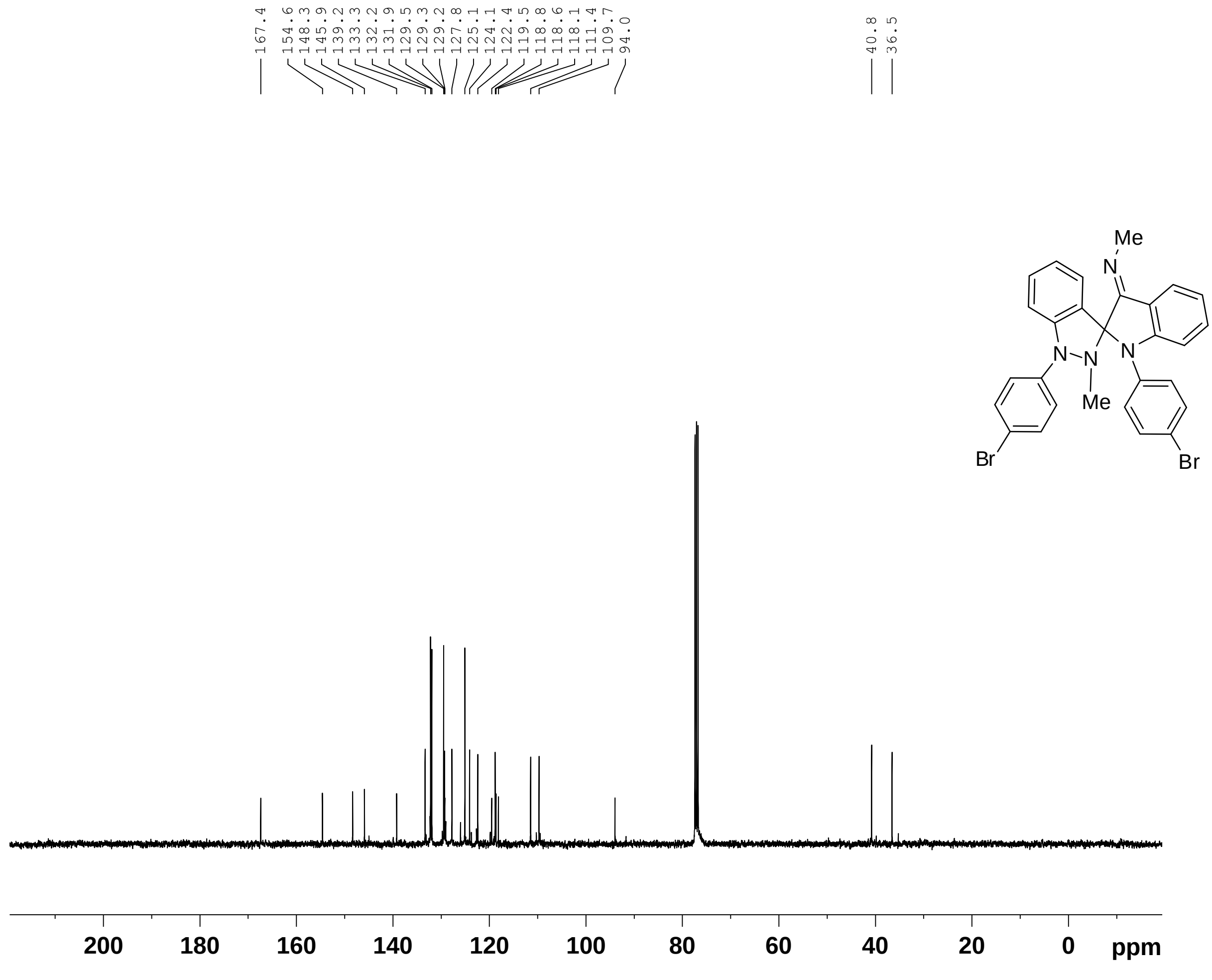
167.4
154.6 148.3 145.9 139.2 133.3 132.2 131.9 129.5 129.3 129.2 127.8 125.1 124.1 122.4 119.5 118.8 118.6 118.1 111.4 109.7 94.0
40.8 36.5
200 180 160 140 120 100 80 60 40 20 0 ppm

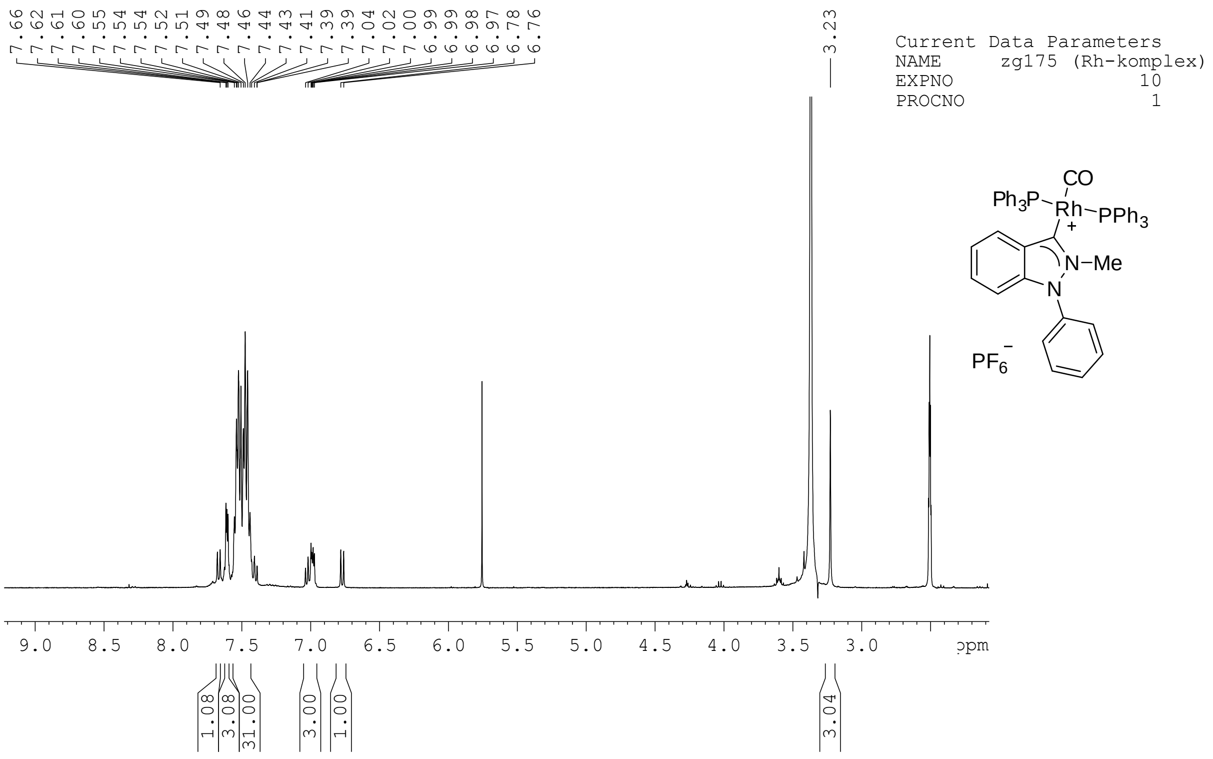
7.66 7.62 7.61 7.60 7.55 7.54 7.54 7.52 7.51 7.49 7.48 7.46 7.44 7.43 7.41 7.39 7.39 7.04 7.02 7.00 6.99 6.99 6.98 6.97 6.78 6.76
3.23
Current Data Parameters
NAME zg175 (Rh-komplex)
EXPNO 10
PROCNO 1
CO
Ph3P
Rh
PPh3
N–Me
N
PF6
9.0 8.5 8.0 7.5 7.0 6.5 6.0 5.5 5.0 4.5 4.0 3.5 3.0 ppm
1.08 3.08 31.00 3.00 1.00 3.04

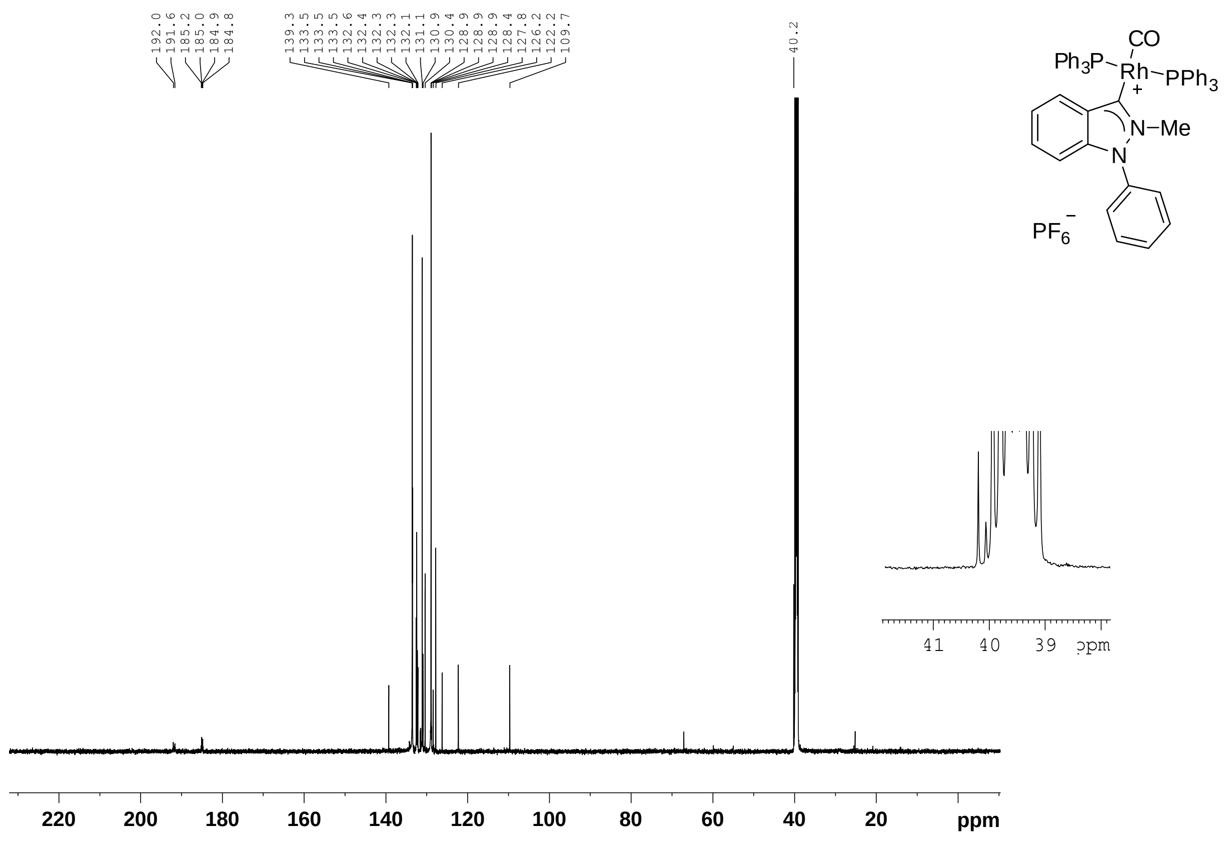
192.0 191.6 185.2 185.0 184.9 184.8
139.3 133.5 133.5 132.6 132.4 132.3 132.1 131.1 130.9 130.4 128.9 128.9 128.4 127.8 126.2 122.2 109.7
40.2
CO
Ph3P
Rh
PPh3
N–Me
N
PF6
41 40 39 ppm
220 200 180 160 140 120 100 80 60 40 20 ppm

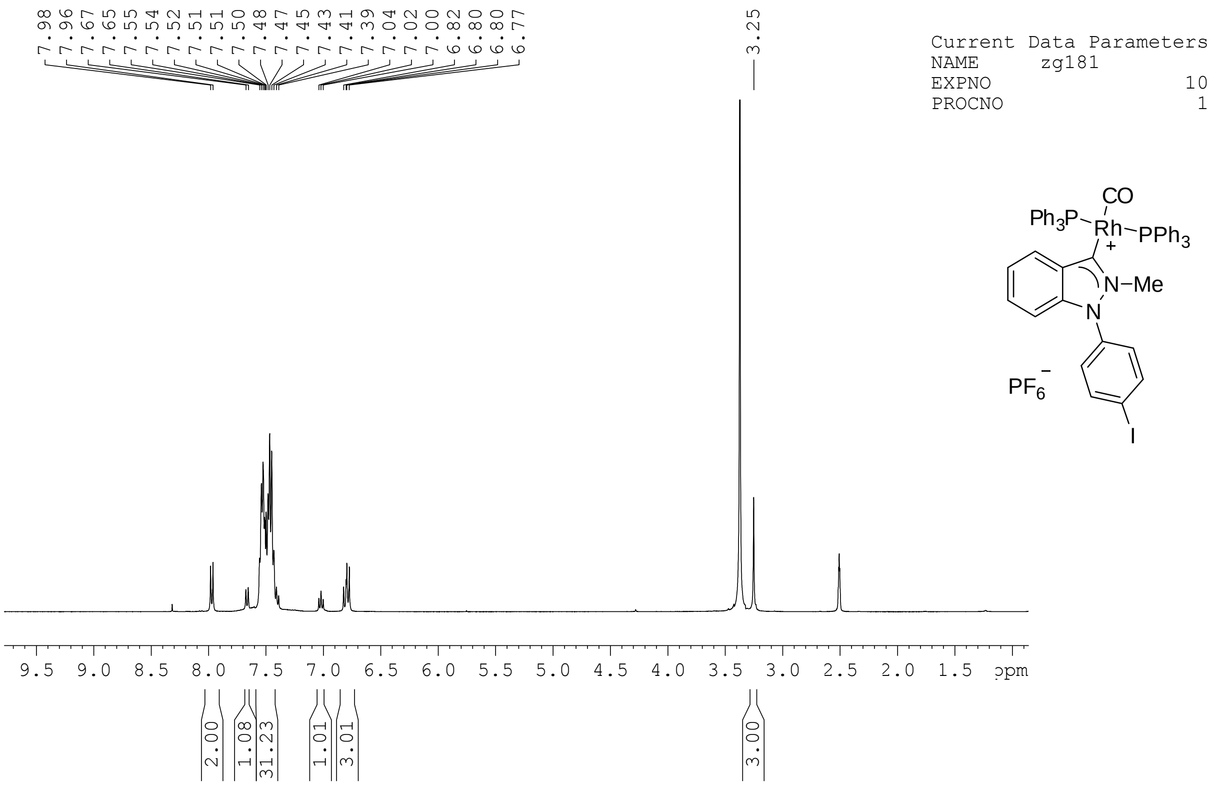

7.98
7.96
7.67
7.65
7.55
7.54
7.52
7.51
7.51
7.50
7.48
7.47
7.45
7.43
7.41
7.39
7.04
7.02
7.00
6.82
6.80
6.80
6.77
3.25
Current Data Parameters
NAME zg181
EXPNO 10
PROCNO 1
CO
Ph3P
Rh
PPh3
+
N–Me
N
PF6
I
9.5 9.0 8.5 8.0 7.5 7.0 6.5 6.0 5.5 5.0 4.5 4.0 3.5 3.0 2.5 2.0 1.5 ppm
2.00
1.08
31.23
1.01
3.01
3.00

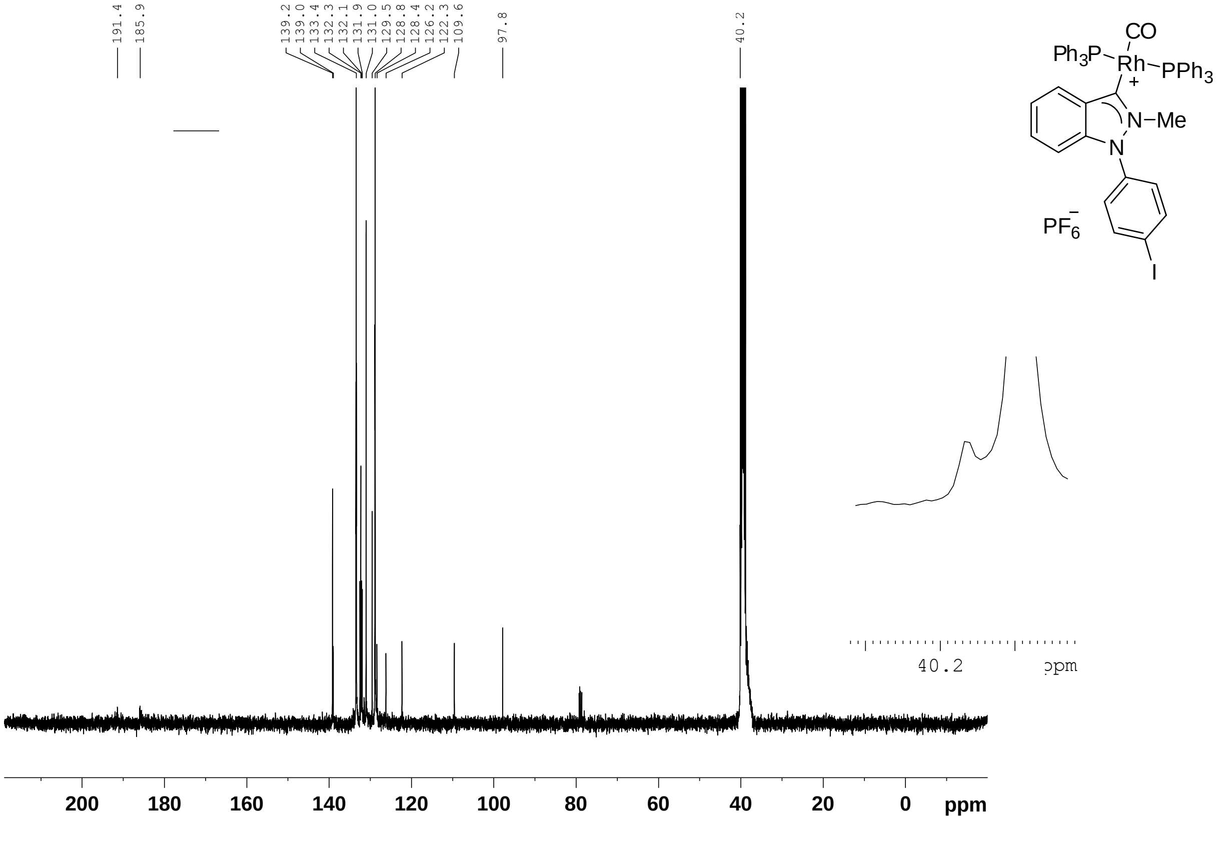

191.4
185.9
139.2
139.0
133.4
132.3
132.1
131.9
131.0
129.5
128.8
128.4
126.2
122.3
109.6
97.8
40.2
CO
Ph3P
Rh
PPh3
+
N–Me
N
PF6
I
40.2
ppm
200 180 160 140 120 100 80 60 40 20 0 ppm

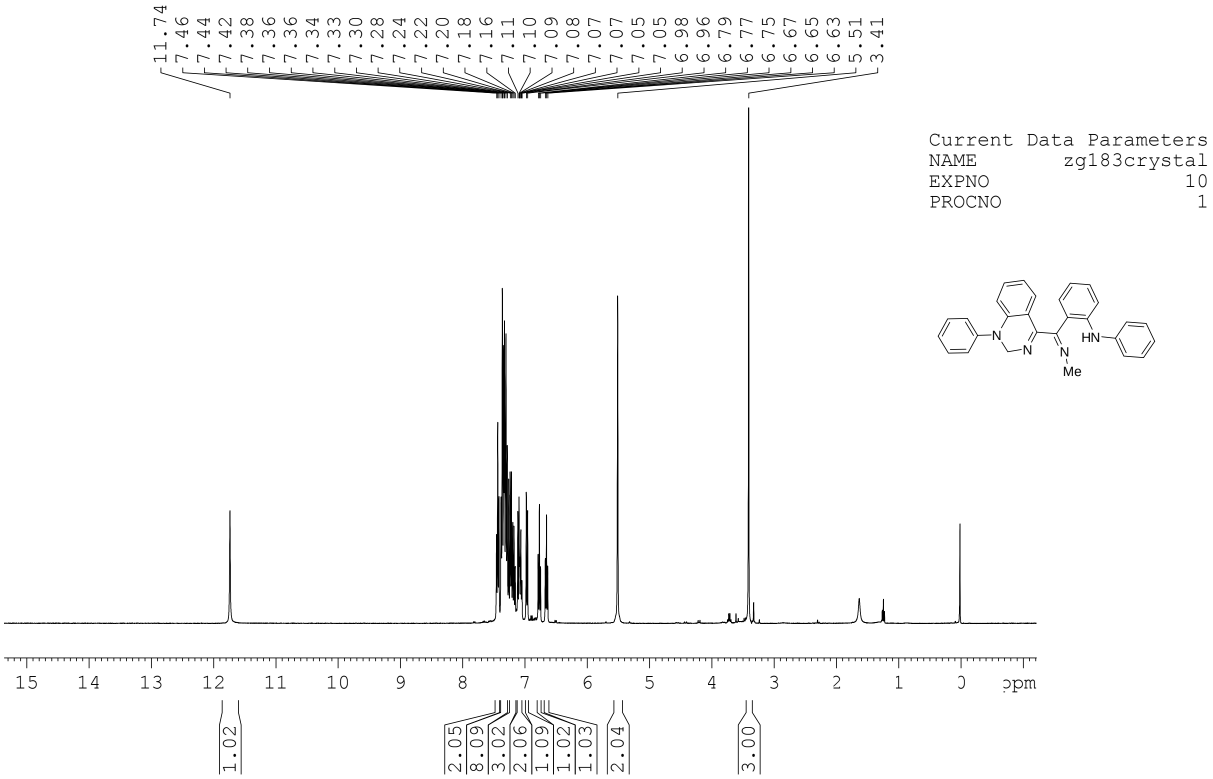

11.74
7.46
7.44
7.42
7.38
7.36
7.36
7.34
7.33
7.30
7.28
7.24
7.22
7.20
7.18
7.16
7.11
7.10
7.09
7.08
7.07
7.07
7.05
6.98
6.96
6.79
6.77
6.75
6.67
6.65
6.63
5.51
3.41
Current Data Parameters
NAME zg183crystal
EXPNO 10
PROCNO 1
N
N
HN
N
Me
15 14 13 12 11 10 9 8 7 6 5 4 3 2 1 0 ppm
1.02
2.05
8.09
3.02
2.06
1.09
1.02
1.03
2.04
3.00

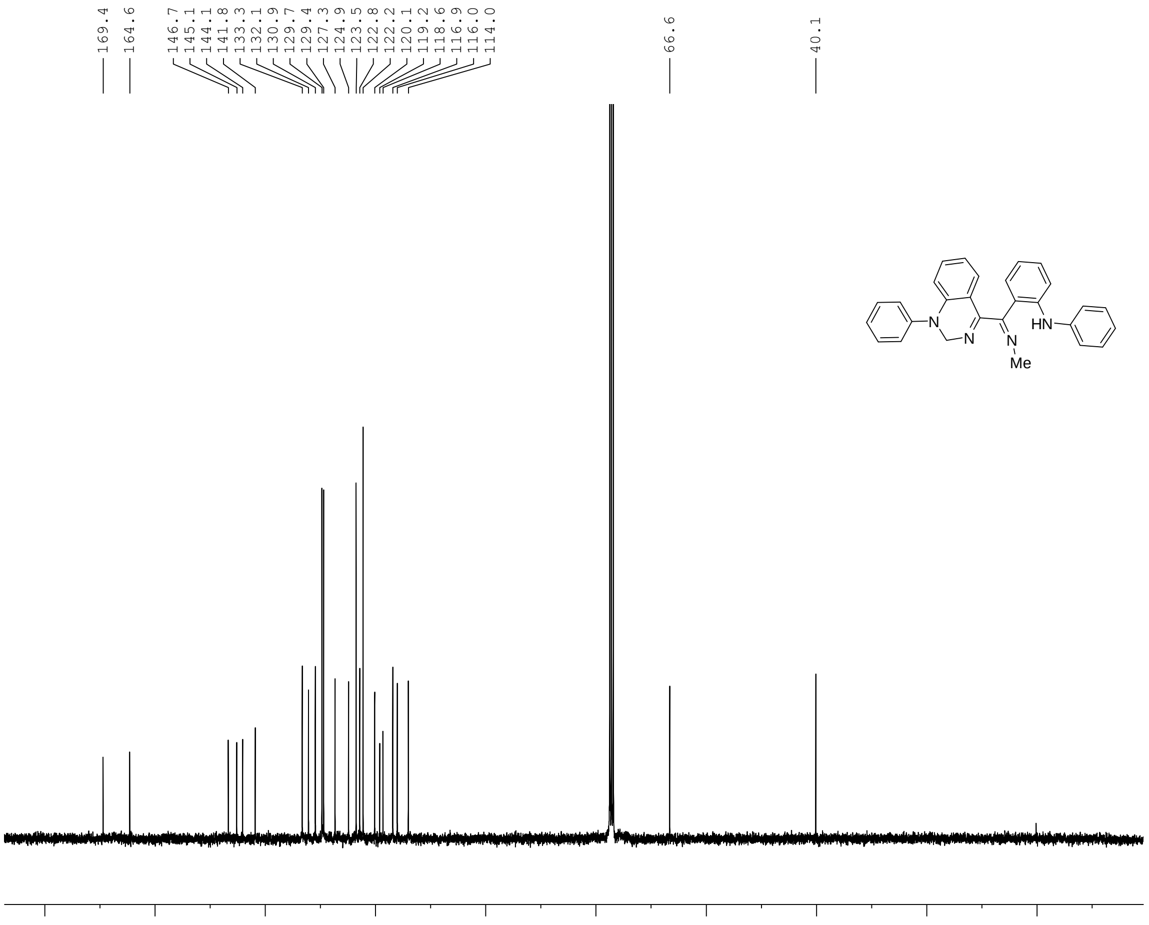

169.4
164.6
146.7
145.1
144.1
141.8
133.3
132.1
130.9
129.7
129.4
127.3
124.9
123.5
122.8
122.2
120.1
119.2
118.6
116.9
116.0
114.0
66.6
40.1
N
N
HN
N
Me
180 160 140 120 100 80 60 40 20 0 ppm

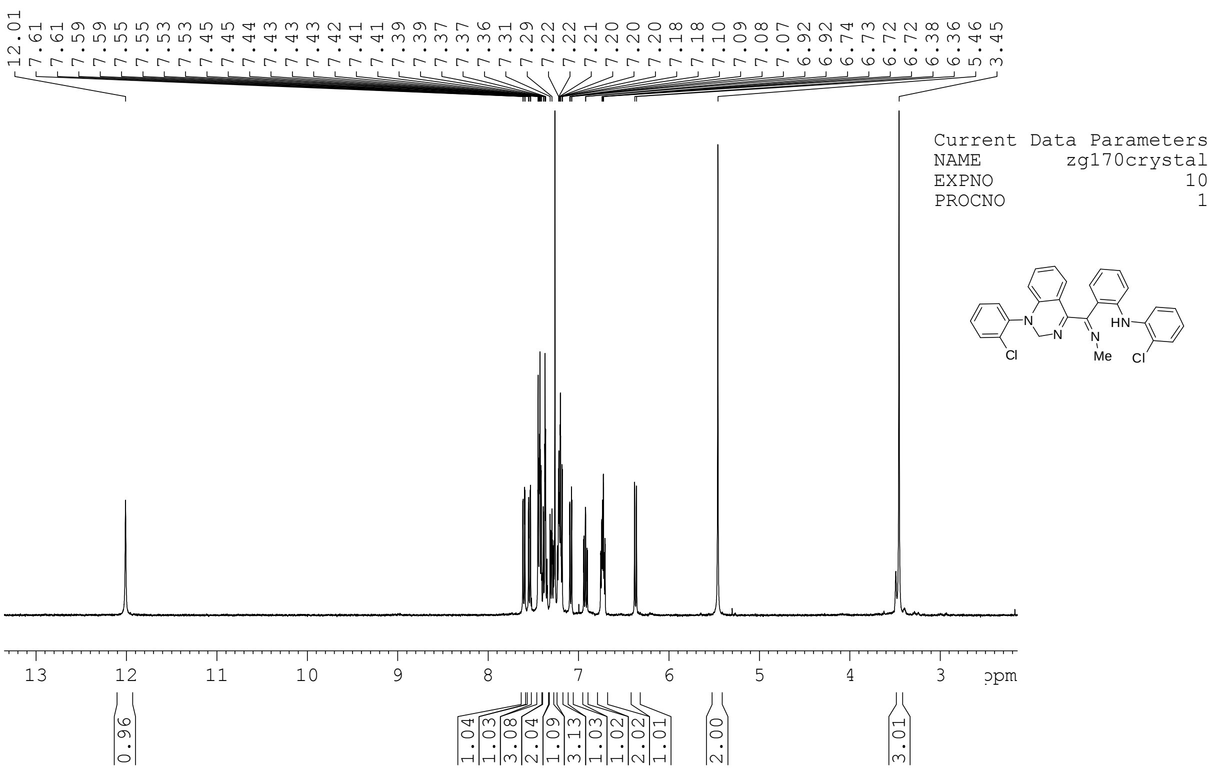
Current Data Parameters
NAME zg170crystal
EXPNO 10
PROCNO 1
ppm

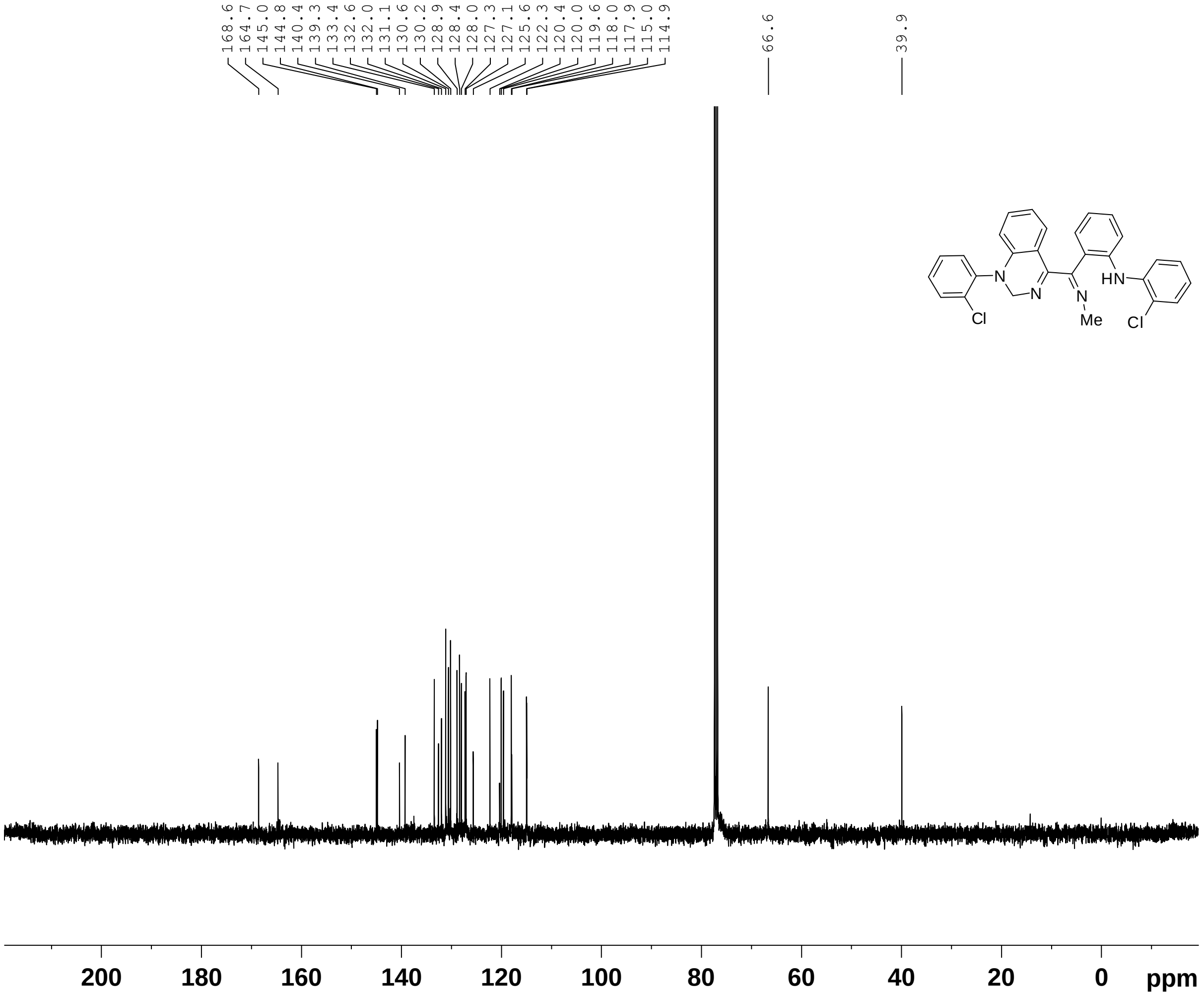
ppm

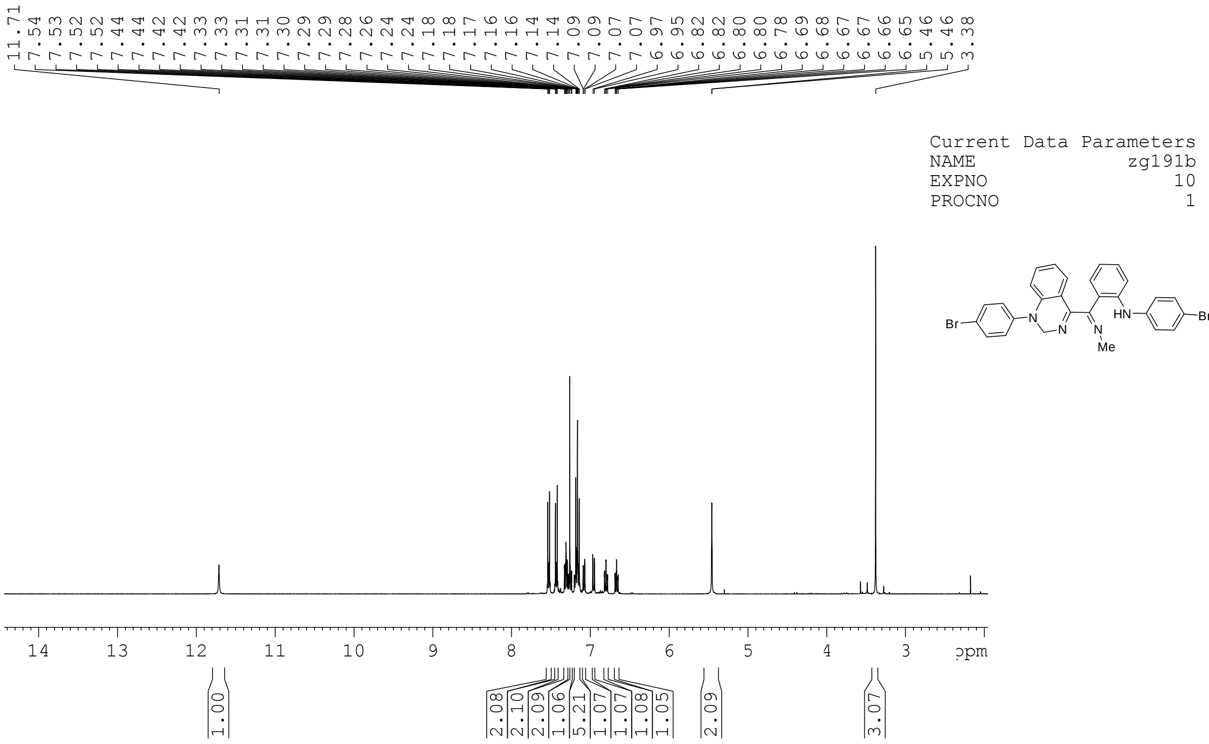
Current Data Parameters
NAME zg191b
EXPNO 10
PROCNO 1
Br
N
N
HN
Br
N
Me
ppm

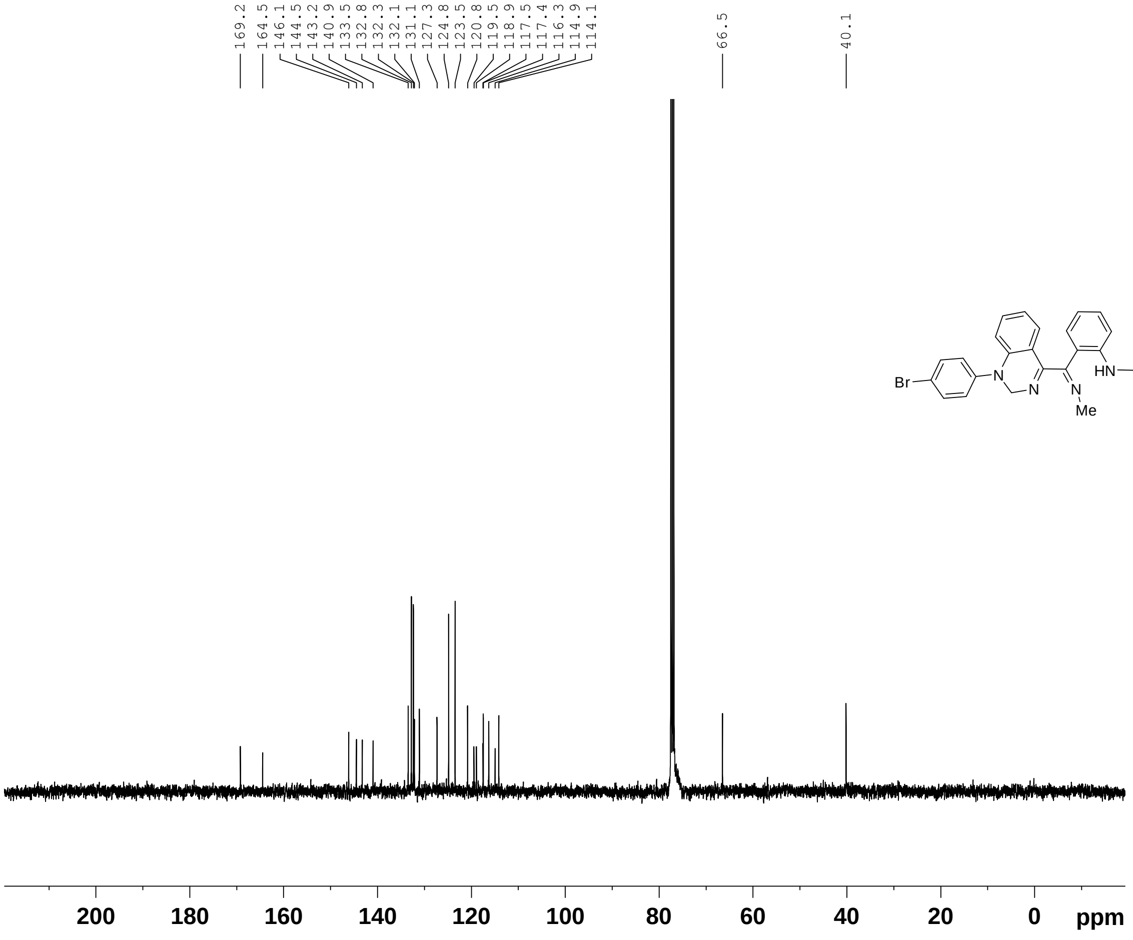
169.2
164.5
146.1
144.5
143.2
140.9
133.5
132.8
132.3
132.1
131.1
127.3
124.8
123.5
120.8
119.5
118.9
117.5
117.4
116.3
114.9
114.1
66.5
40.1
Br
N
N
HN
Br
N
Me
ppm

DFT calculations

13a

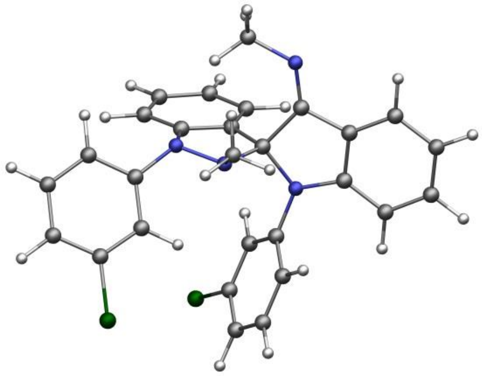

E = -2219.540102 Hartree

G = -2219.163455 Hartree

N1	8.3290087605	6.5090242772	8.9539300885
N2	8.1304025415	6.3762944264	7.5183705395
C3	9.2828113635	6.8969885342	6.8998320052
C4	9.6633331024	6.8265966492	5.5570890522
H5	9.0745691981	6.2749653670	4.8326979514
C6	10.8333195302	7.4846647227	5.1759624839
H7	11.1453478972	7.4424398790	4.1357673992
C8	11.6097529578	8.1939268847	6.1009782348
H9	12.5130854630	8.7009399354	5.7751862499
C10	11.2289882200	8.2363696030	7.4441353267
H11	11.8324169022	8.7622957936	8.1792469344
C12	10.0715241918	7.5701224926	7.8323378984
C13	9.4616255103	7.4279634143	9.2035961673
N14	10.3909256602	6.8376667372	10.2179489725
C15	10.4075806564	7.6015886797	11.3835480786
C16	11.0829616030	7.3493590493	12.5829695234
H17	11.6651296112	6.4443228335	12.7198845713
C18	10.9907590651	8.3029852314	13.5986471223
H19	11.5087816462	8.1187423598	14.5367002311
C20	10.2556287698	9.4863226454	13.4389978262
H21	10.2073402291	10.2072552498	14.2497131768
C22	9.5935449673	9.7337478603	12.2367445479
H23	9.0200653184	10.6415230929	12.0743239329
C24	9.6725272048	8.7871525483	11.2182116693
C25	9.0910581637	8.8067659625	9.8754284179
N26	8.4478679553	9.8034554828	9.4114175840
C27	7.8842162136	9.8477689990	8.0753550588
H28	8.5829315454	10.3721345647	7.4092221123
H29	6.9668714750	10.4468091591	8.1095061419
H30	7.6485735036	8.8771353595	7.6233471392
C31	7.0690413578	6.7676807168	9.6435458409
H32	6.5590015775	7.6808378379	9.3094855185
H33	7.2774526312	6.8451133522	10.7140081388
H34	6.4027741490	5.9138358059	9.4904742098
C35	7.6115634761	5.1097158866	7.1051032614
C36	6.8746629129	5.0579002496	5.9155297501
H37	6.6922223671	5.9737208405	5.3621274267
C38	6.3617506331	3.8412693763	5.4699012491
H39	5.7949194332	3.8030155872	4.5437606337
C40	6.5408076347	2.6757118037	6.2151591873

H41	6.1267052298	1.7291749144	5.8853691239
C42	7.2545429056	2.7545307249	7.4103307554
Cl43	7.4802137376	1.2932243733	8.3676115974
C44	7.8013381413	3.9500509121	7.8669518669
H45	8.3641460349	3.9950369271	8.7911729272
C46	10.7544563787	5.4658638205	10.1731656517
C47	10.2544580572	4.5523970509	11.1126153477
H48	9.5773490465	4.8993381348	11.8867646281
C49	10.6190771324	3.2087952451	11.0450688644
H50	10.2237961674	2.5056594210	11.7728881730
C51	11.4677112615	2.7470274086	10.0374416141
H52	11.7425835922	1.7000246757	9.9707005501
C53	11.9551413828	3.6637198554	9.1087053484
Cl54	13.0379280180	3.1075496519	7.8372295958
C55	11.6220352194	5.0145875773	9.1683571014
H56	12.0319743144	5.7122789073	8.4491114804

Transition state between 13a and 14a

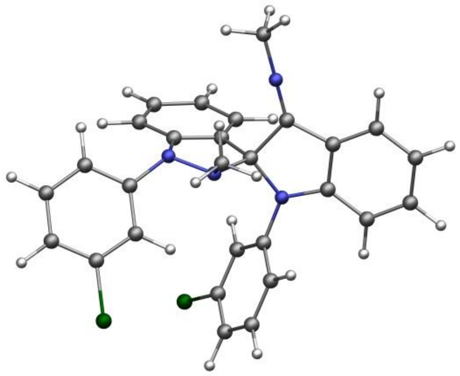

E = -2219.506116 Hartree

G = -2219.132645 Hartree

N1	8.3393439739	6.5096155174	8.9586363923
N2	8.1502475403	6.3834444824	7.5244575362
C3	9.2662782296	6.9679750705	6.9013060111
C4	9.6229689504	6.9621667145	5.5490107620
H5	9.0287647255	6.4371036209	4.8101304867
C6	10.7811390835	7.6446845226	5.1754737114
H7	11.0723874649	7.6505648056	4.1282791968
C8	11.5716905732	8.3163955935	6.1156143834
H9	12.4663109767	8.8425418407	5.7958015776
C10	11.2148789352	8.2962739285	7.4658328931
H11	11.8297501839	8.7924613191	8.2123281229
C12	10.0699252882	7.6052317199	7.8473994884
C13	9.4821334307	7.4151150430	9.2186395237
N14	10.4041229885	6.8300513603	10.2309990511
C15	10.4253188770	7.6067678812	11.3887598598
C16	11.1121948876	7.3611564700	12.5843290454
H17	11.6979813710	6.4580383682	12.7184469216
C18	11.0278785208	8.3155948517	13.5992710095
H19	11.5560392244	8.1335014516	14.5320773664
C20	10.2888121154	9.4975713391	13.4471863717
H21	10.2474783314	10.2197878411	14.2571520449
C22	9.6158698021	9.7387342219	12.2498778065
H23	9.0395075138	10.6477088304	12.0962611706
C24	9.6857226088	8.7928262692	11.2308719407

C25	9.0832026786	8.8089781133	9.8844869082
N26	8.4140512203	9.6807913553	9.3132790043
C27	7.6587856713	10.6647995495	8.6685615295
H28	7.0323376666	11.2538304503	9.3599197154
H29	7.0060948690	10.2696374776	7.8700941298
H30	8.3484629439	11.3740401160	8.1880984900
C31	7.0664448837	6.8090317345	9.6103342814
H32	6.5840436654	7.7185991294	9.2299527986
H33	7.2466121237	6.9162404764	10.6835889735
H34	6.3910005879	5.9606976894	9.4646608187
C35	7.6442880346	5.1232977759	7.0930032843
C36	6.9608583398	5.0612386928	5.8703643992
H37	6.8061288909	5.9701923909	5.2982613943
C38	6.4561196971	3.8446896400	5.4167102706
H39	5.9322759546	3.8017062696	4.4656423184
C40	6.5858376208	2.6856662310	6.1823977032
H41	6.1769159249	1.7396062753	5.8450022687
C42	7.2422388694	2.7745040968	7.4089753261
Cl43	7.4059140119	1.3233793351	8.3961143913
C44	7.7802333248	3.9693340581	7.8767737625
H45	8.3013134529	4.0190116093	8.8246897308
C46	10.7671473998	5.4584111384	10.1960919368
C47	10.2851371777	4.5544469208	11.1539680154
H48	9.6275923892	4.9100252400	11.9409203661
C49	10.6386163867	3.2080322031	11.0851647796
H50	10.2560800731	2.5120570409	11.8265899846
C51	11.4574741428	2.7339821094	10.0589656043
H52	11.7226318502	1.6845844285	9.9911999924
C53	11.9257781075	3.6415841500	9.1114728494
Cl54	12.9697036053	3.0691080771	7.8145572264
C55	11.6031997545	4.9947825323	9.1703037498
H56	11.9937248235	5.6851572655	8.4331098658

14a

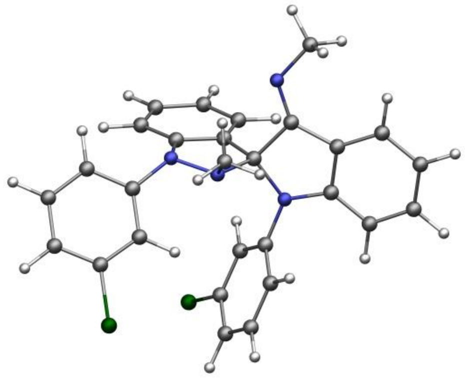

E = -2219.540051 Hartree

G = -2219.163633 Hartree

N1	8.2987214263	6.5527682021	8.9742666014
N2	8.1311957251	6.3852252224	7.5423701618
C3	9.2471220184	6.9589024649	6.9127824944
C4	9.6160068570	6.9190828302	5.5637760826
H5	9.0290274413	6.3747309512	4.8334433738
C6	10.7737585063	7.5964928710	5.1824641633
H7	11.0734122719	7.5771902266	4.1378036312
C8	11.5536365590	8.2967747981	6.1110517284

H9	12.4476691153	8.8199337285	5.7847812381
C10	11.1852344467	8.3103818621	7.4575031417
H11	11.7899675009	8.8316612429	8.1950608068
C12	10.0400825579	7.6246878606	7.8459471142
C13	9.4545740850	7.4488400526	9.2231496538
N14	10.3911023416	6.8353957072	10.2148305408
C15	10.4232294635	7.5812432296	11.3826720480
C16	11.1143113413	7.2901548244	12.5646227159
H17	11.6970803753	6.3802095788	12.6574492405
C18	11.0337325428	8.1994506608	13.6180295945
H19	11.5644269905	7.9805015422	14.5412780775
C20	10.2877400154	9.3802018396	13.5152241721
H21	10.2377112778	10.0670623379	14.3545509607
C22	9.6117128983	9.6740893881	12.3316275814
H23	9.0327743817	10.5863522035	12.2565395277
C24	9.6803913590	8.7800298715	11.2555937146
C25	9.1070584360	8.8070361393	9.9055825378
N26	8.4546891338	9.6677300642	9.2267082985
C27	8.1293083241	10.9633629913	9.7939896701
H28	7.3869779069	10.8789017785	10.6015464926
H29	7.6991804079	11.5964130532	9.0121751916
H30	9.0121922359	11.4757481093	10.2026731923
C31	7.0169387397	6.9037253022	9.5863408240
H32	6.5885339697	7.8276529468	9.1804078244
H33	7.1680579784	7.0124591248	10.6644040226
H34	6.3197702534	6.0760099327	9.4257407565
C35	7.6249730784	5.1176403793	7.1379589582
C36	6.9540884604	5.0281195806	5.9098406340
H37	6.8063964621	5.9239500282	5.3158100527
C38	6.4515993473	3.8022624003	5.4795686286
H39	5.9374325673	3.7381847294	4.5244070392
C40	6.5705873729	2.6609634665	6.2733673235
H41	6.1635882684	1.7078794694	5.9538292913
C42	7.2133134908	2.7778898808	7.5046805730
Cl43	7.3632667594	1.3494620764	8.5275709112
C44	7.7487039277	3.9821759912	7.9502854461
H45	8.2592462528	4.0523377826	8.9024197797
C46	10.7655683726	5.4676891865	10.1418184025
C47	10.2619878712	4.5315926428	11.0561329521
H48	9.5741307365	4.8576211749	11.8300097028
C49	10.6346440611	3.1921445585	10.9611589693
H50	10.2367144079	2.4699494068	11.6684719983
C51	11.4940854033	2.7594245795	9.9500181827
H52	11.7757589838	1.7158070725	9.8618783992
C53	11.9820496924	3.6987092226	9.0446042926
Cl54	13.0758862132	3.1764438659	7.7689497757
C55	11.6400549284	5.0461039038	9.1312184278
H56	12.0453669655	5.7624915857	8.4275234541

14a inverted at indazole ring

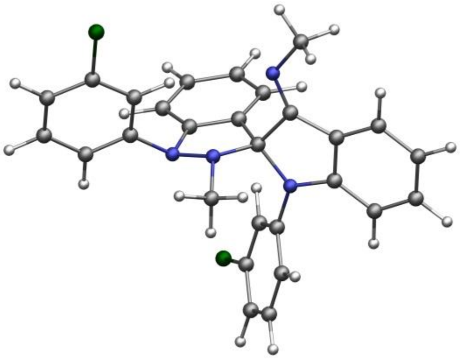

E = -2219.536186 Hartree

G = -2219.160154 Hartree

C1	0.0965568426	-0.0944918883	0.0424928874
C2	0.0294843126	-0.2023006686	1.5956982153
N3	1.1326470841	-0.1651204247	2.2332103911
C4	1.1614811876	-0.2618001786	3.6809832814
H5	3.3973005026	-0.7308361909	-2.9867402309
C6	2.7236876097	-2.6161656463	-2.1611614460
H7	3.4112909143	-3.2058508245	-2.7615027400
C8	1.8625812216	-3.2615346469	-1.2633415003
H9	1.8896284308	-4.3432979421	-1.1710300580
C10	0.9760941719	-2.5165624091	-0.4831782711
H11	0.3078714070	-3.0066790490	0.2198192246
C12	0.9686422048	-1.1324335136	-0.6201149003
N13	0.7693223951	1.1419210184	-0.3494013025
N14	-1.3550126047	-0.2046397102	-0.3429316577
C15	-2.1516271776	-0.3293951401	0.7876694952
C16	-3.5424205098	-0.4847490182	0.8472656073
H17	-4.1397781969	-0.5112766265	-0.0569026573
C18	-4.1436881784	-0.6138507122	2.0984853291
H19	-5.2229749460	-0.7341317818	2.1503863773
C20	-3.3945819937	-0.5951978786	3.2808958519
H21	-3.8900186253	-0.6943892545	4.2419006509
C22	-2.0087152166	-0.4596761245	3.2197579893
H23	-1.4285983448	-0.4567189284	4.1340721089
C24	-1.3781969848	-0.3320993580	1.9754563130
N25	1.6382985901	0.9031464999	-1.4949914047
C26	1.8237793540	-0.4981782947	-1.5184526501
C27	2.7212529433	-1.2284979412	-2.2992072666
H28	0.6263049878	0.5712094069	4.1601165733
H29	2.2013318653	-0.2318784061	4.0201495194
H30	0.7145106379	-1.1995840878	4.0424657606
C31	0.0044497478	2.3693061404	-0.4749125589
H32	-0.6326354455	2.4039856509	-1.3679976624
H33	0.6980111737	3.2158559053	-0.5049530361
H34	-0.6160502164	2.4784094348	0.4194578314
C35	2.8580129641	1.6725888908	-1.3921137933
C36	3.4010057389	2.2171851803	-2.5552541388
H37	2.8762558335	2.0863760438	-3.4967185788
C38	4.6009013027	2.9293647482	-2.4871810446
H39	5.0297013815	3.3547616142	-3.3902598215
C40	5.2452946925	3.1183123971	-1.2661974788
H41	6.1700457056	3.6818559928	-1.2021346581
C42	4.6747472145	2.5734333077	-0.1135116301
Cl43	5.4896680620	2.8058494789	1.4320273699

C44	3.4912132112	1.8467089341	-0.1544547670
H45	3.0490669353	1.4137111151	0.7373298615
C46	-1.8643756812	-0.0836254271	-1.6624761501
C47	-2.8386311735	0.8886453259	-1.9486750044
H48	-3.1779825112	1.5497284142	-1.1577729606
C49	-3.3542578474	1.0137375891	-3.2354816822
H50	-4.1060795914	1.7705123637	-3.4420596815
C51	-2.9001835829	0.1943092537	-4.2700692680
H52	-3.2862303217	0.2946664613	-5.2785539979
C53	-1.9328733790	-0.7631997377	-3.9767713186
Cl54	-1.3561961852	-1.8200786257	-5.2612991854
C55	-1.4199922606	-0.9274839540	-2.6917099642
H56	-0.6955274504	-1.7053518904	-2.4934011914

Reference

[1] Lebedev, A. Y; Khartulyari, A.S.; Voskoboynikov, A. Z. *J. Org. Chem*, **2005**, *70*, 596.
